# Methyltransferase Domain‐Focused Genome Mining for Fungal Polyketide Synthases

**DOI:** 10.1002/smtd.202400107

**Published:** 2024-04-21

**Authors:** Dexiu Yan, Yudai Matsuda

**Affiliations:** ^1^ Department of Chemistry City University of Hong Kong Tat Chee Avenue Kowloon Hong Kong SAR China

**Keywords:** biosynthesis, methyltransferase domains, natural products, polyketide synthases

## Abstract

A comparison of substrate‐binding site amino acid residues in the *C*‐methyltransferase (MT) domains of fungal nonreducing polyketide synthases (NR‐PKSs) suggests that these residues are correlated with the methylation modes used by the PKSs. A PKS, designated as AsbPKS, with substrate‐binding site residues distinct from those of other known PKSs is focused on. The characterization of AsbPKS revealed that it yields an isocoumarin derivative, anhydrosclerotinin B (**1**), the biosynthesis of which involves a previously unreported methylation pattern. This study demonstrates the utility of MT domain‐focused genome mining for the discovery of PKSs with new functions.

## Graphical abstract

Genome mining is performed with a focus on the substrate‐binding amino acid residues in the *C*‐methyltransferase domains of fungal nonreducing polyketide synthases (NR‐PKSs). An NR‐PKS designated as AsbPKS is discovered and found to be responsible for the formation of anhydrosclerotinin B (**1**), the biosynthesis of which adopts a previously unreported methylation pattern.

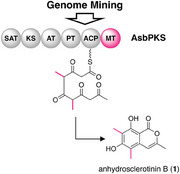

## 1 Introduction

Polyketides are a diverse group of natural products that exhibit a wide range of structural and functional properties. They have significant implications for pharmaceutical applications, as they include antibiotics (e.g., tetracycline^[^ ^1^ ^]^ and erythromycin^[^ ^2^ ^]^), anticancer drugs (e.g., epothilone^[^ ^3^ ^]^ and doxorubicin^[^ ^4^ ^]^), cholesterol‐lowering agents (e.g., lovastatin^[^ ^5^ ^]^), immunosuppressants (e.g., rapamycin^[^ ^6^ ^]^), and environmental carcinogenic factors (e.g., aflatoxin B1^[^ ^7^ ^]^). The biosynthesis of polyketides is governed by a family of multifunctional enzymes known as polyketide synthases (PKSs), which are classified into three main types, namely, types I,^[^ ^8^, ^9^, ^10^, ^11^ ^]^ II,^[^ ^12^, ^13^, ^14^ ^]^ and III.^[^ ^15^, ^16^ ^]^ Type I PKSs are further divided into modular PKSs, which consist of multiple modules, each of which is used only once during polyketide chain synthesis, and iterative PKSs (iPKSs), which are composed of a single module that can be repeatedly used for polyketide chain synthesis and processing. In fungi, the majority of polyketides are synthesized by type I iPKSs, which are categorized into three groups: nonreducing (NR)‐PKSs, partially reducing (PR)‐PKSs, and highly reducing (HR)‐PKSs.^[^ ^11^ ^]^ All type I iPKSs include three essential domains for chain elongation: acyltransferase (AT), ketosynthase (KS), and acyl carrier protein (ACP) domains. In addition, several optional domains, such as *C*‐methyltransferase (MT), ketoreductase (KR), dehydratase, and enoylreductase domains, can be embedded in type I iPKSs. Importantly, these optional domains do not function in every elongation cycle. Thus, the programming rules of fungal type I PKSs, which control the number of chain elongations and the timing of the reactions catalyzed by the optional domains, have been intensively studied for more than a decade.^[^ ^17^, ^18^, ^19^, ^20^, ^21^ ^]^ However, it is still challenging to predict the product of an uncharacterized fungal type I iPKS, and further studies on fungal PKSs are required for an in‐depth understanding of their programming rules.

MT domains, which are found in HR‐ and NR‐PKSs, catalyze the transfer of a methyl group from *S*‐adenosylmethionine (SAM) to the α‐carbon of β‐ketoacyl substrates bound to ACP during the catalytic cycle of the polyketide chain synthesis. MT domains are in competition with other PKS domains, such as KS and KR domains, and methylation only occurs when an MT domain functions before the β‐ketoacyl substrate is accepted by these other domains, resulting in the selective installation of methyl groups at specific positions.^[^ ^22^, ^23^, ^24^ ^]^ Structural biology^[^ ^25^, ^26^, ^27^ ^]^ and domain swapping^[^ ^23^ ^]^ studies have been performed to understand the catalytic mechanism and the programming rules of MT domains in fungal PKSs. Notably, a structural biology study of the MT domain of PksCT (CitS), which is responsible for the biosynthesis of citrinin, revealed the first 3D structure of the MT domain of a fungal PKS, providing insights into the binding mode of the substrate and cofactor.^[^ ^25^ ^]^ Nevertheless, the underlying basis and mechanisms governing methylation programming remain inadequately understood.

In this study, we focused on the amino acid residues at the substrate‐binding sites of MT domains in fungal type I NR‐PKSs and investigated the relationship between these residues and methylation programming rules. Our findings suggest that substrate‐binding site residues are key determinants of methylation patterns. Furthermore, genome mining targeting PKSs with substrate‐binding site residues distinct from those of other known PKSs led to the identification and characterization of the first anhydrosclerotinin B synthase.

## 2 Results and Discussion

### 2.1 Comparison of the Amino Acid Residues at the Active Sites of MT Domains in Fungal Type I NR‐PKSs

**Figure 1:**
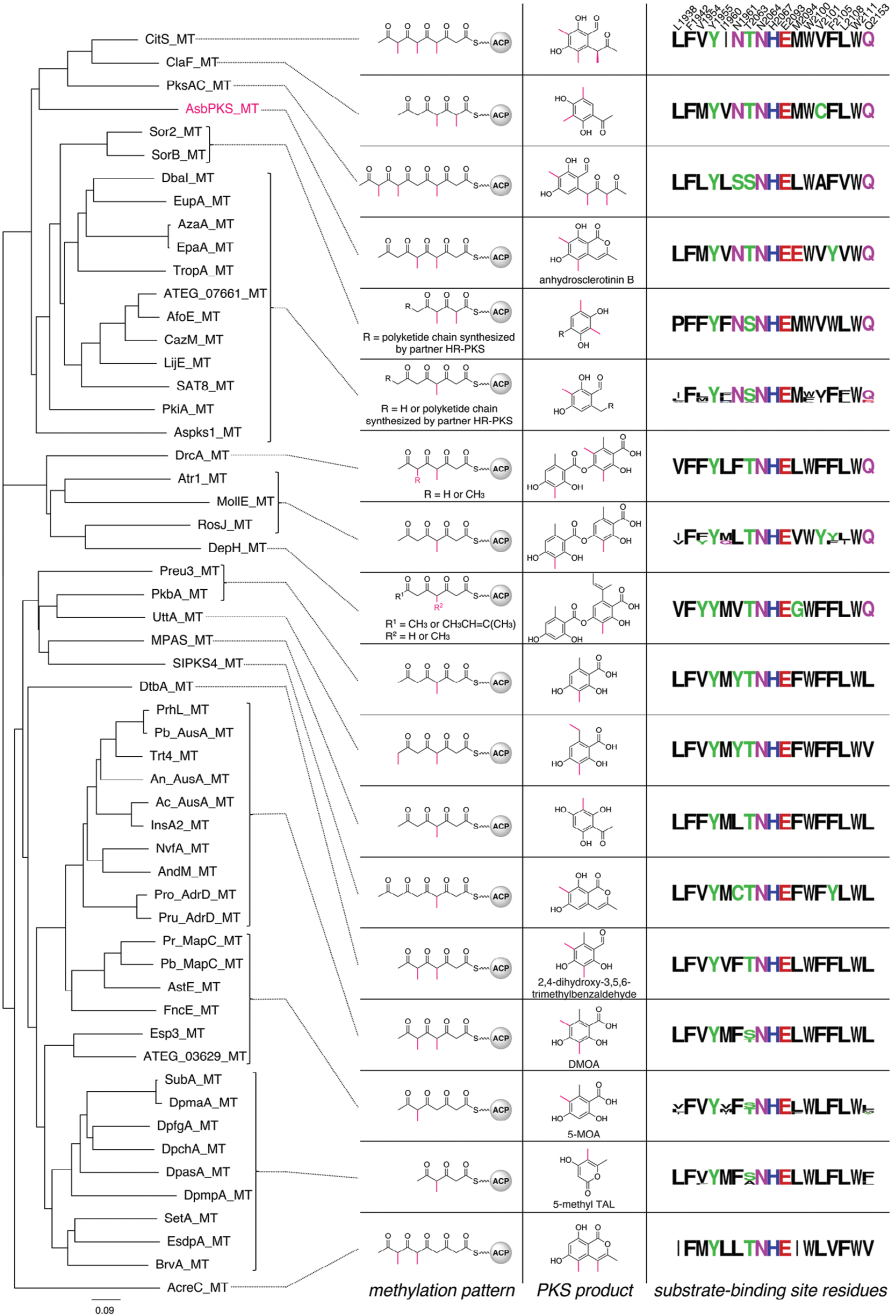
Phylogenetic analysis of the MT domains in known fungal nonreducing polyketide synthases (NR‐PKSs) and their methylation patterns, products, and substrate‐binding site residues. The sequence logos were created using WebLogo.^[^ ^40^ ^]^ The PKS sequences are provided in the Supporting Information.

The crystal structure of the MT domain of CitS revealed several amino acid residues that make up the substrate‐binding pocket, including Leu1938, Phe1942, Val1954, Tyr1955, Ile1960, Asn1961, Thr2063, Asn2064, His2067, Glu2093, Met2094, Trp2100, Val2101, Phe2105, Leu2108, Trp2111, and Gln2153 (Figure S1, Supporting Information), of which His2067 and Glu2093 serve as a catalytic dyad.^[^ ^25^ ^]^ We hypothesized that the substrate‐binding site residues of the MT domains of fungal PKSs are correlated with their methylation programming. To investigate this hypothesis, we compared the 17 substrate‐binding site residues of known fungal NR‐PKSs harboring an MT domain (**Figure** **1**). This comparison revealed that MT domains with identical or similar programming have resemblant substrate‐binding site residues regardless of their phylogenetic distances (Figure 1). For instance, PKSs that yield 3,5‐dimethylorsellinic acid (DMOA), such as AusA,^[^ ^28^ ^]^ Trt4,^[^ ^29^ ^]^ and AndM,^[^ ^30^ ^]^ all have highly conserved substrate‐binding site residues. Intriguingly, DtbA,^[^ ^31^ ^]^ which forms the aldehyde analog of DMOA, 2,4‐dihydroxy‐3,5,6‐trimethylbenzaldehyde, also possesses similar substrate‐binding site residues to those of the DMOA synthases, although DtbA is not phylogenetically closely related to DMOA‐synthesizing PKSs. The MT domains of PKSs for 5‐methylorsellinic acid (5‐MOA)^[^ ^32^, ^33^, ^34^, ^35^, ^36^ ^]^ also resemble those of DMOA synthases; however, one clear distinction is that the amino acid residues corresponding to Val2101 of CitS are conserved as phenylalanine in DMOA synthases and DtbA, whereas leucine is found at the corresponding sites of 5‐MOA synthases. Notably, the substrate‐binding residues of PKSs that generate 5‐methyl triacetic acid lactone (5‐methyl‐TAL)^[^ ^37^, ^38^, ^39^ ^]^ are also similar to those of 5‐MOA synthases, and they harbor a conserved leucine residue, as seen in 5‐MOA synthases. This observation is consistent with the fact that methylation only occurs during the first‐round of chain elongation in both 5‐MOA and 5‐methyl‐TAL synthases. Collectively, these data suggest correlations between the substrate‐binding site residues of MT domains and their functions, at least to some extent.

### 2.2 Investigation of the Importance of the Substrate‐Binding Residues of MT domains in Methylation Programming

**Figure 2:**
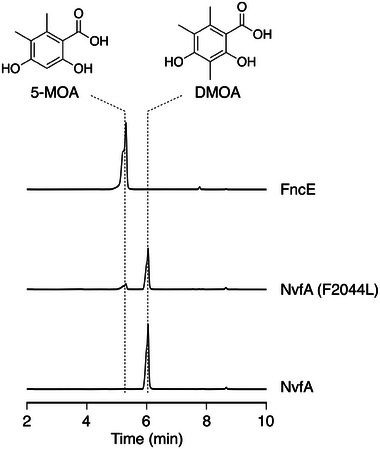
HPLC profiles of metabolites from *A. oryzae* transformants expressing NvfA, NvfA (F2044L), or FncE. The chromatograms were monitored at 265 nm.

To investigate whether differences in the substrate‐binding residues of MT domains contribute to the methylation programming of fungal PKSs, we next sought to introduce a point mutation into a PKS to achieve functional conversion. To this end, we focused on NvfA,^[^ ^41^ ^]^ the DMOA synthase from the novofumigatonin pathway, and aimed to transform it into a 5‐MOA synthase. Thus, an NvfA variant in which Phe2044 (corresponding to Val2101 of CitS) was replaced with leucine was heterologously expressed in *Aspergillus oryzae* NSAR1.^[^ ^42^ ^]^ The metabolites of the *A. oryzae* transformant were analyzed by high‐performance liquid chromatography (HPLC) and compared with those of transformants containing wild‐type NvfA or the 5‐MOA synthase FncE^[^ ^35^ ^]^ (**Figure** **2**; Figure S2, Supporting Information). The F2044L variant of NvfA yielded 5‐MOA, although DMOA was still produced as a major product. This observation indicated that the KS domain, which competes with the MT domain, is also a key factor that determines the methylation pattern. Our recent study on the depside‐forming PKS ThiA also demonstrated that the MT domain is not the sole contributor to methylation programming.^[^ ^43^ ^]^ Indeed, the phylogenetic tree of the KS domains exhibited a similar pattern to that of the MT domains (Figure S3, Supporting Information), perhaps suggesting the co‐evolution of KS and MT domains. Nevertheless, the partial functional conversion of NvfA by a single mutation in the MT domain supports our hypothesis that substrate‐binding residues of MT domains can dictate the methylation programming of fungal PKSs.

### 2.3 Genome Mining and Functional Analysis of a PKS with an Unreported Methylation Pattern

**Figure 3:**
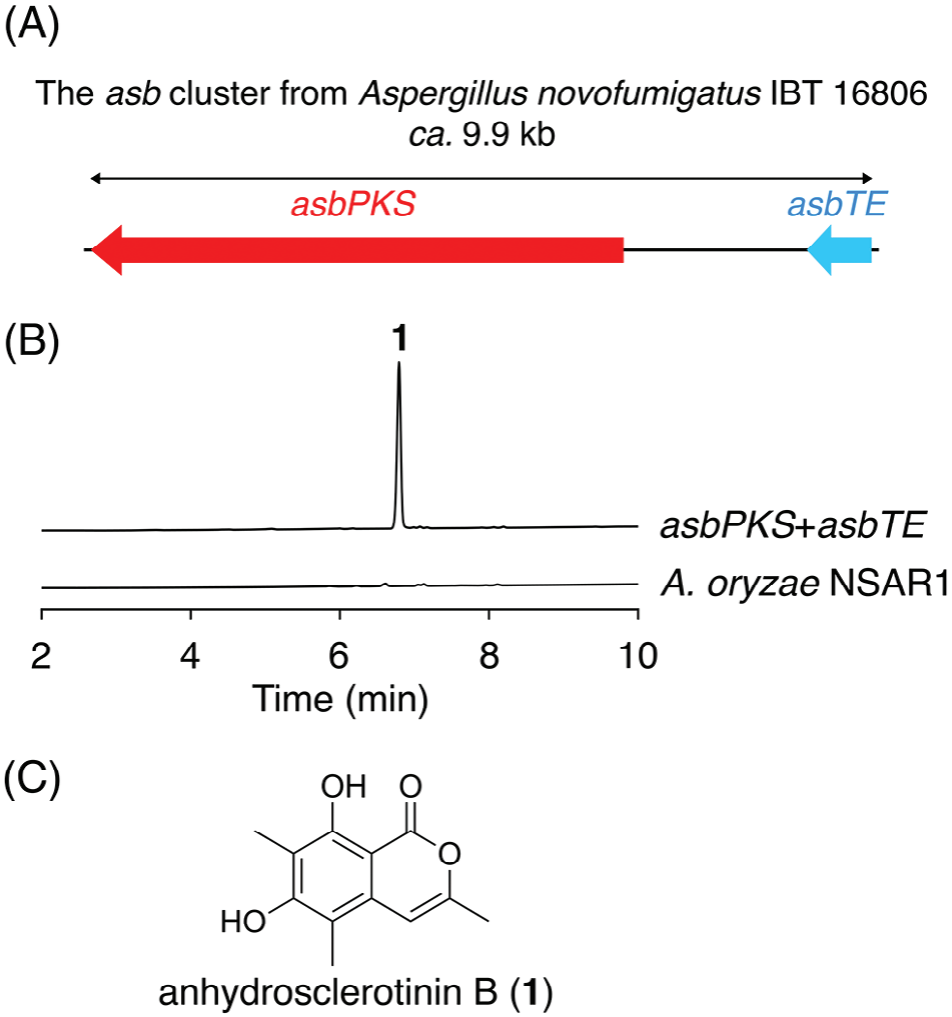
Functional analysis of AsbPKS. A) Schematic representation of the *asb* cluster. B) HPLC profile of the metabolites from the *A. oryzae* transformant expressing AsbPKS and AsbTE. The chromatograms were monitored at 240 nm. C) The structure of anhydrosclerotinin B (**1**).

On the basis of the bioinformatic analysis and the mutational experiment described above, we expected that functional analysis of an uncharacterized PKS with an MT domain harboring substrate‐binding residues distinct from those of known PKSs would lead to the discovery of a PKS with an unreported methylation pattern. Thus, we examined the substrate‐binding residues of fungal NR‐PKSs available in the National Center for Biotechnology Information database and focused on one PKS encoded by *Aspergillus novofumigatus* IBT 16806 (GenBank: XP_024688196.1; Table S1, Supporting Information), which was designated as AsbPKS and possesses a domain organization of starter‐unit acyltransferase (SAT)–KS–AT–product template (PT)–ACP–MT. The substrate‐binding residues of the MT domain of AsbPKS include a glutamate residue at the corresponding site of Met2094 in CitS, at which nonpolar amino acid residues are found in characterized NR‐PKSs (Figure 1). Thus, it was expected that AsbPKS exhibit a methylation pattern different from known PKSs. Meanwhile, the flanking region of the PKS gene encodes a putative thioesterase named AsbTE (GenBank: XP_024688197.1) (**Figure** **3**A), which displays 47% amino acid sequence identity with the esterase CitA involved in polyketide chain release from CitS during citrinin biosynthesis.^[^ ^44^ ^]^ Thus, AsbTE should work together with AsbPKS, especially given that AsbPKS lacks a domain for chain release.

**Figure 4:**
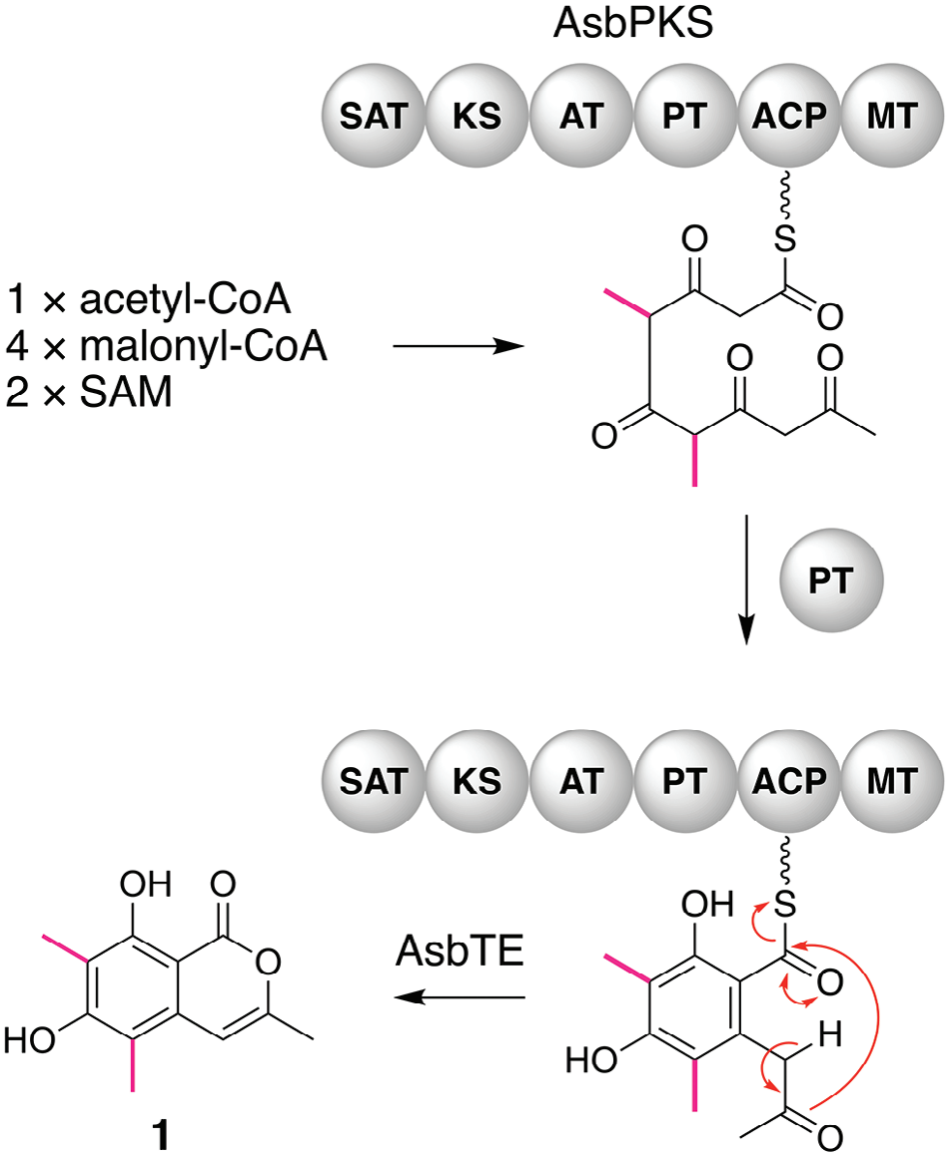
Proposed mechanism for the formation of anhydrosclerotinin B (**1**).

To investigate the function of AsbPKS, the PKS gene *asbPKS* was introduced into *A. oryzae* NSAR1,^[^ ^42^ ^]^ along with the esterase gene *asbTE*, which resulted in the formation of a specific metabolite **1** with the molecular formula C_12_H_12_O_4_ (Figure 3B). Subsequently, **1** was isolated from a large‐scale cultivation of *A. oryzae* and subjected to nuclear magnetic resonance (NMR) analysis for structural determination. NMR analysis identified **1** as an isocoumarin derivative, anhydrosclerotinin B (Figure 3C), which was first obtained as a dehydrated product of sclerotinin B^[^ ^45^, ^46^ ^]^ and was later reportedly isolated from the fungus *Keissleriella* sp. YS4108.^[^ ^47^ ^]^ During the biosynthetic process of **1**, the NR‐PKS AsbPKS synthesizes a pentaketide, and methyl groups are installed in the second and third rounds of chain elongation (**Figure** **4**). The pattern of methylation by AsbPKS is different from the patterns for previously characterized PKSs (Figure 1), thus demonstrating the utility of our MT domain‐targeted genome mining method for the discovery of PKSs with new functions. The biosynthetic role of the esterase AsbTE is currently unclear; however, it may be responsible for the chain release and lactonization required to complete the formation of **1**.

Finally, we investigated the importance of the glutamate residue specifically found in AsbPKS (Glu2052) in methylation programming. To this end, we replaced the glutamate residue with methionine or leucine, as found in CitS and PksAC, respectively (Figure 1), and coexpressed the individual PKS variant and AsbTE in *A. oryzae*. Both variants yielded **1** as the major product; however, LC–MS analysis revealed the emergence of a minor metabolite **2** in these variants (Figure S4, Supporting Information). Although we were unable to determine the structure of **2** due to its low productivity, **2** displayed a similar UV spectrum to that of **1** and appeared to possess one additional methyl group compared to **1** based on its MS spectrum (Figure S4, Supporting Information). Thus, Glu2052 somehow influences the methylation pattern of the polyketide chain; however, it is indicated that the KS domain of AsbPKS plays a significant role in the methylation programming of the PKS, as also observed in the mutational experiment of NvfA.

## 3 Conclusion

In this study, we investigated the relationship between the substrate‐binding residues of MT domains in fungal NR‐PKSs and their methylation programming. Our findings suggest that the substrate‐binding residues are key determinants of the methylation pattern in the polyketide chain and are optimized to yield a specifically methylated product. We also conducted MT domain–focused genome mining, which successfully resulted in the discovery of a PKS that exhibits a previously unreported methylation pattern. Considering that a number of unexploited PKSs can be found in publicly available databases, a similar genome mining approach may be applied to discover more PKSs with unprecedented methylation patterns, which would increase our understanding of the programming rules of fungal PKSs.

## 4 Experimental Section

### General Experimental Procedures

Organic solvents were purchased from Anaqua (Hong Kong) Co., Ltd., and other chemicals were purchased from Wako Chemicals Ltd., Thermo Fisher Scientific, and J&K Scientific Ltd. unless noted otherwise. Oligonucleotide primers (Table S2, Supporting Information) were purchased from the Beijing Genomics Institute. Polymerase chain reaction (PCR) was performed using a T100 Thermal Cycler (Bio‐Rad Laboratories) with Phanta Max Super‐Fidelity DNA Polymerase (Vazyme Biotech Co., Ltd.). Analytical HPLC was performed on a Dionex Ultimate 3000 UHPLC system (Thermo Fisher Scientific) using a Kinetex 2.6 µm C_18_ 100 Å column (2.1 × 100 mm; Phenomenex). Semipreparative HPLC was performed on a Waters 1525 Binary HPLC pump with a 2998 photodiode array detector (Waters Corporation). Flash chromatography was performed using an Isolera Spektra One flash purification system (Biotage). NMR spectra were obtained at 600 MHz (^1^H)/150 MHz (^13^C) with a Bruker Ascend Avance III HD spectrometer, and chemical shifts were recorded with reference to solvent signals (^1^H NMR: dimethyl sulfoxide [DMSO]‐*d*_6_ 2.50 ppm; ^13^C NMR: DMSO‐*d*_6_ 39.5 ppm). High‐resolution electrospray ionization mass spectrometry spectra were obtained using a SCIEX X500R Q‐TOF mass spectrometer. Samples for LC‐MS analysis were injected into a SCIEX ExionLC AD System with a SCIEX X500R Q‐TOF mass spectrometer, using a Luna Omega C18 column (1.6 µm, 100 Å, 2.1×100 mm; Phenomenex).

### Strains

*Aspergillus novofumigatus* IBT 16806 (IFM 55215) was used as a source for the cloning of *nvfA* and each gene in the *asb* cluster. *Aspergillus funiculosus* CBS 116.56 was purchased from the Westerdijk Fungal Biodiversity Institute and used as a source for the cloning of *fncE*. *Aspergillus oryzae* NSAR1 (*niaD^−^, sC^−^, ΔargB, adeA^−^*)^[^ ^42^ ^]^ was used as the fungal heterologous expression host. Standard DNA engineering was performed using *Escherichia coli* DH5α (Takara Bio Inc.).

### Phylogenetic Analysis

To extract the sequences of the MT domains, the collected sequences of fungal NR‐PKSs (see Supporting Information) were analyzed using the “hmmscan” tool in HMMER software^[^ ^48^ ^]^ (version 3.3.2) against the custom‐made HMM profile for the MT domains (PKS_NRPS_MT.hmm) created in previous study.^[^ ^49^ ^]^ The detected domain envelopes of each PKS, along with 30 additional amino acid residues at the C terminus, were extracted as MT domains. The sequence alignment and phylogenetic analysis were performed using the Geneious Tree Builder in Geneious Prime 2024.0.3 (https://www.geneious.com); the MT domains were first aligned with the global alignment method and Blosum62 as the cost matrix, and the phylogenetic tree was created using the Jukes–Cantor model and the Neighbor‐Joining tree build method. The phylogenetic tree of the KS domains was also created in a similar manner, in which KS domains were extracted using the HMM profile for the KS domains (PKS_KS.hmm; SM00825) from the SMART^[^ ^50^ ^]^ database.

### Construction of Fungal Transformation Plasmids

To construct expression plasmids for *A. oryzae*, *nvfA*, *asbPKS*, and *asbTE* were first amplified from the genomic DNA (gDNA) of *A. novofumigatus* IBT 16 806, whereas *fncE* was amplified from the gDNA of *A. funiculosus* CBS 116.56 using the primers listed in Tables S2 and S3 (Supporting Information). Each amplified DNA fragment was then introduced into the pTAex3‐HR vector^[^ ^38^ ^]^ using a ClonExpress Ultra One Step Cloning Kit (Vazyme Biotech Co., Ltd.). The transformation plasmid encoding the F2044L variant of NvfA was created using a PCR‐based method using a pair of mutation‐specific primers (Table S2, Supporting Information). To construct a plasmid with both *asbPKS* and *asbTE*, *asbTE*, along with the *amyB* promoter (*PamyB*) and the *amyB* terminator (*TamyB*), were amplified from a pTAex3‐HR‐based plasmid and further introduced into pTAex3‐HR‐asbPKS, yielding pTAex3‐HR‐asbPKS+asbTE. The plasmids encoding the E2052M and E2052L variants of AsbPKS were constructed by utilizing pairs of mutational primers (Tables S2 and S3, Supporting Information). The detailed methods for the construction of the plasmids used in this study are presented in Table S3 (Supporting Information).

### Fungal Transformation

*A. oryzae* NSAR1 was transformed using a pTAex3‐HR‐based plasmid by CRISPR‐Cas9‐mediated DNA double‐strand break and repair by homologous recombination, as previously described.^[^ ^38^ ^]^ The transformants created in this study and the plasmids used for transformation are presented in Table S4 (Supporting Information).

### HPLC and LC‐MS Analysis of the Metabolites Derived from the A. oryzae Transformant

To analyze the metabolites produced by each *A. oryzae* transformant, the transformant was cultivated on a DPY agar plate (2% dextrin, 1% hipolypepton [Nihon Pharmaceutical Co., Ltd., Seattle, WA, USA], 0.5% yeast extract, 0.5% KH_2_PO_4_, 0.05% MgSO_4_•7H_2_O, and 1.5% agar) for approximately one week at 30 °C. A small piece of fungal mycelia with agar was cut from the plate, soaked in ethyl acetate, and extracted using an ultrasonic bath. The ethyl acetate layer was transferred to a new tube, and the solvent was removed using nitrogen gas flow. The residue was dissolved in methanol and analyzed by HPLC and LC‐MS, with a solvent system of 20 mm formic acid (solvent A) and acetonitrile containing 20 mm formic acid (solvent B), at a flow rate of 0.4 mL min^−1^ and a column temperature of 40 °C. Separation was performed using a linear gradient from 10:90 (solvent B/solvent A) to 100:0 for 10 min, 100:0 for 3 min, a linear gradient from 100:0 to 10:90 for 2.0 min, and then 10:90 for 2.5 min of equilibration.

### Isolation of compound **1**

To isolate **1**, *A. oryzae*/*asbPKS+asbTE* was cultivated on 50 DPY agar plates (≈20 mL of medium per plate) for approximately one week at 30 °C. The resulting fungal cultures, including the agar medium, were crushed into small pieces, soaked in ethyl acetate, and extracted twice using an ultrasonic bath. After filtration, ethyl acetate was removed in vacuo. The resultant crude extract (0.78 g) was subjected to flash chromatography and eluted stepwise using a dichloromethane:acetone gradient (100:0 to 0:100). Fractions containing **1** (62.1 mg) were then concentrated and further purified by reverse‐phase preparative HPLC [37% aqueous acetonitrile containing 0.05% trifluoroacetic acid, 8.0 mL min^−1^ using an XBridge BEH C_18_ OBD Prep Column (100 Å, 5 µm, 19 i.d. x 250 mm; Waters Corporation)] to yield 33.1 mg of **1**.

### Anhydrosclerotinin B (**1**)

White amorphous solid; for NMR data, see Figures S5 to S9 (Supporting Information); HRMS (ESI) *m/z*: [M + H]^+^ calcd for C_12_H_12_O_4_ 221.0808; found 221.0801.

## Conflict of Interest

The authors declare no conflict of interest.

## Supporting information

Supporting Information

## Data Availability

The data that support the findings of this study are available in the supplementary material of this article.
