# Supplementary material for: Methyltransferase Domain‐Focused Genome Mining for Fungal Polyketide Synthases
Source: Small Methods. 2024 Apr 21;8(11):2400107. doi: 10.1002/smtd.202400107 (PMC11579551; doi:10.1002/smtd.202400107)
Supplement: Supplementary file 1 — Supporting Information [file SMTD-8-2400107-s001.pdf]

small
methods

Supporting Information

for *Small Methods*, DOI 10.1002/smtd.202400107

Methyltransferase Domain-Focused Genome Mining for Fungal Polyketide Synthases

*Dexiu Yan and Yudai Matsuda**

WILEY-VCH

Supporting Information

Methyltransferase Domain-Focused Genome Mining for Fungal Polyketide Synthases

*Dexiu Yan and Yudai Matsuda**

Table S1. Revised DNA and protein sequences. Highlighted in magenta are the regions predicted for an intron in this study.

Gene	Revised DNA sequence	Prevised protein sequence
asbPKS	atgcatcacatcagtggaaaagcgtgcgtacttctttttgggcccttggccctagggctcgattcggcgtcattactcaaaacacacaagctactgatagatccggactac<br>gattcccagtgggtgattgatgtcctcgaagagcttccggcgtactgggagctactagcaaaggaaatcccggccctatgccattgttatggcgcatcaaagctccgtgat<br>ttgaagggatggtttcaaaccggcaacatcccggctgacgcttttccacttccgaacattcttcttgtgccttctgctattgtcatgcagcttgtggaatatcagcgatttgccaa<br>acttgcaaacgttgggaggaagcattccgaagcctactacggcaacacagaagcctttgggctgtgtacggggctcctgagtgcactcgccgtggctcagtccaccac<br>ccaagaagagctacatcaatatggtgctgtagccatccgcctggccatggttgttggggctactgtggacgccgaggacacgtcagatgggaaggctatgggcaagtc<br>ccgatccttctcagtggcctgggaccaagacacctcgtgggaagacattgacaatattctgcagcggtttccagaggtatgcgtccactgccaaatctaaaacattaaga<br>gctataagatttacagcatgtcagtcacaggcctacgcgtccgttctcttcgataagcgacgcgtgactatcacgacatccaagggcgcgtccgcctctttattgcagagc<br>ctccacgaggttccaggagtgactattacagctatcaagttgcacgggcgatttcaccacgtgtgccatcaggaagtcctgggccgccttgcagagttctgtaattccaat<br>agtgcctttcactttcgagacgattctggtccaccgatttctctgcgcgtcggctgtgcgaagcccgaacaaggagagcagaaactacatatcctggccctacggtctata<br>ctggttgaccagtccctatggtatgaagccttccgtgaaatccaaagtctgatattgtcaagcgaaagttcacgcctggtctgtttcgggcaagagcgatccattccaccct<br>cacttcagcaagcaatctcttctcgaattgtccacccgactgagctcctggtaccagccaaggcatgcgcagatccatatcagtctaccaaaatatcctacaggggttgc<br>aatggctcagtcaaccaccatgcagttgctgtggtcggtatggcatgcgaggtccctggagcaacggatctggatgaatttgcagccctattgaagcagggcaagtcgc<br>agcatgtggaagtccccagcgagcgagtggacttctacaccccatggcgcgagactgacagtaagcgccgctggtatggaaatttcctggataactatgacatgtttga<br>tcacaagtttttcaacaagacggcgcgcgagatggcttctaccgatccccaacatcggcttgttttgcaggtggcgtaccaagcattgcaacaagcgggttacttccgctg<br>gaaagatgctgataaaaacatcggctgctttattgcgatgggtttaaccgattatgagcagaacgttgcttgccacccgcctaatgcatattctgctacggggaatctcagg<br>agcttcgctgctggccgtatcagtcactatttcggctggacagggcccgcattggtcattgataccgcttgttcttcctcggcagtcgctgtccatcaagcatgtcaagccttg<br>aagagcggagaatgttctgctgcactcgtttgcggagtgagtcttatgacaagtccgcaatggtttcagaacctcgctggagcctcgtttcttagcccaacagggcaatgc<br>aagccattcgacgctgctgcagatggatactgtcggggagaggcggtgggtgctgtgttcctgaaaagctatcagaatgctgtgagccaccatgatcaaatcctgggtct<br>catcgccggttcagccgtctcccaaaatcgcaattgcaccccccattacggtcccaaacgccgccagcctttcggatctttaccgctctgtgatcgaaaaagccggtctgg<br>agccaagctccatatccgtcgtcgaggcacacggaacaggcactccggtgggtgatcccgcagaatttgacagtgtccgccaagtattcagtggccaggagagaatt<br>ggccggctgcatcttggatctgttaaggggcttgttgggcatagtgaggcagccgcagggattgtctctctgatcaaagtcatcctgatgatgcatgagcgcttcattccgc<br>cacagtctagcttcgatacaatcaatccagcaattaaacggtcagcgaacgatcaaattgagatcaccaagaatctgcaaccatggacctccgacttttatgctgcgctc<br>gttaatagttacggcgcctcgggatcgaatgcctcgctggttctcacagggtcccccgtacgtttgaccgaaacggaagacaatggttatgcggaagctgctaatgtgaa<br>atacccctttcgcatatacggtttcgacacgcctagtcttcagagatatgtaactcggttccgccgatatctgagtaccgcaggacaacatgtttctgtcgctggcctggccc<br>accaattggcgttgcagtcaaatccgacacttccttacgcgctagggttttccgccagaaccaaggaggaattaatcgataagctaggtatcatcgaaagtggtgctcag<br>gcccctgcagcagtccggacggatggagcgaggccggtcattctatgctttgggggtcaaatttcaagatacatcgggctcgaccgagacatatactatcatatagatat<br>tttccgccaatatctagaccgatgcaacgcatactgtgagaagcttggtcttggtagcatcttcccggggatctttcaacgcgcgccagtggaagatatcgtgaaactcca<br>gacgatgctcttcgccctccaatatacttgcgccaaatgctggctggattgtggagtctcagttgcggcagtggttggccacagctttggggaattgaccgctctttgcatctc<br>cggtgccattttttggaagatgctcttctcgccatcgctggacgggcccgtctgatccaggagtactggggtgcggacaaaggatctatgttggctgtcgagggagacttg<br>gctgtggtgaaggagctgctagctactgcgaatgctacagcagagccctcccctcctgcaaatatcgcttgcttcaatgcgccgcgcatattcaccttggcaggtcccac<br>acattccatcaatgtggtcttcgacatggcccgcggacccaagtatgcatcacttgttagggctaagaaattagaggtcacgaatgcgttccattctgctttagttgaccctct<br>tgcgacacatttgcaaaagctggggcagaacctgatgttccaggccccgaagatccatatcgagcgagcaactgcagaggccgagtttatctctccaggtgtccagtc<br>agacatattgctgaatcatatgagaaacccagtatacttccaccatgcggccaagcgcctggcccagcgatatccgttcagtgtttgggtggaagccggagcaaactca<br>tcgattacgtccatggcccgccaagcactgggggatctttgtgaacattctcaacatgcgtttcagtcgttgaatattaccagcggtgcaggagtgggattcaactcccttgt<br>ggatgcgacgacgtccttgtggaatgaaggcctgaatatcgtcttttggggtcactgttcgaccgaaccggggaggtacaaccctctcctactgccgccatatcagtttgc<br>aaagacgcgcaattggatggagattaccagacggaatatcccagccgtcggcaaagccgacgcactggcgtctgcaggacaaaacattgacgatgtcaatcgccta<br>tggaaactcgttatctccgaggaagatggacgtcgctcggcccgctttgagatcaattcttcgtctcaagtcttccgcgactacttattgggacatgtcgtcgcccagaaag<br>cgcccctagcaactagcgtgttgcagttgcagatcgccctggaagcagtggcaagtttacggtctgactttcaggacggtacgtttctcccagctctcagaggtctgaaca<br>gtcatgctcctatgcttttggatgcgacaaggcgtttctgggtcgatgcggaggctcaggatgctggtggccttacgtgggacctcaaggtgaacagcggagcgagtgat<br>ggcagtggaccaaccacgaaacatacgtcgggacgcattgtgtttgaaaaggcggatgatcccgaaaagctggcagagctttcgaggtacaaacgtgtgatgaaca<br>gtacacggtgcctgagcatgttaaatgccggcgacaacgacagcatcatgcaaggtcccggcatttatcgggcctttaatgatgtggtccagtattccccggcgtatcgg<br>gggctgcagaagattgttgggaagggggcagagtcggcagggagagtggtaatgagcccaccggaagggggatgcatcgacactggcctgttcgactccttctgcc<br>aggtggccggtatctatctgaactgtatgaccgacatgccggagcaatacatgttcatctcgaccaacattgaacgatggatgcgggcgcctacactacactcgcgaga<br>gagatggcctacgtcattcgatgtgcatgtgtgccatgagtatcagtgtgacagcgaagtcttgagcgacgtttttgtgttcgacgcgcagagcggagacttgttcgaagtt<br>atcctcggcatacgctatagtcgcattttgaaggagcgaatgcgccaagtgctgcaccaagccatacctgccccagaaggcagttcccgccttccagcagatgagcca<br>gatccagtcaaggacatgccccataagccagtagtggatgggagaggcaaggagtcctctcagacgggagtatggacgcaactccaagggatgctctgtgatctttgt<br>ggcctggaaccggatgaagtcagacaggactcgcaactgggagacatcggcgtcgattcactcctgggaatggaactggcaagagagatagaaagtcttttccagtg<br>tatgctgaatcaggatgcactcaatgaagctgtggatgtggctgatcttcttcggtgcattgaggaaactcttggtgtgaaacccattacacagaatggctcgacagagaa<br>ggcggccgcatctactttcaaagtatccaacggagacagcatggactccaaaggctgtcagaacggtgtggaaatgtcttttggttcaacaaacaccggcatttcagtc<br>aacaggctaccaagacaggcgctttcagccgcgaaaggctctattgccgcagtcgctacagaagtatttgccagcaccaaggaggccaccgattcgttcattgaaac<br>ctatggcctggctgactatcttgatcaagtcctgccgaggtcgacggagctggttgtggctcatattctggaagcattggaggccttaggatgtcccgtcggtgcagtaccg<br>ccgggcggacgcctgcccaaagtgacgtatctcccaaagcatgagagattcatgaatcttttgtacggactcttggaaaaagcgcggctcattgagatacatgacaag<br>ggcacgtccctcattcggacagccaacccgttgccctcgacgtcagccgaagccctggccagagaactttttagcgactaccccgagcacatttccgaccatcagctta<br>cgcactacacggggaagaacctatccgactgcctcgtcggtcaggccgatatcctgcagattctcttcggctcgccggaatgccgcacgatcatgaccaatatgtacag<br>ccgctcaccagtcaacctggtgtggatcaagcagatgcagtccttcctggagggtgtctggacccagttccgttcgaacgatgggcccgtccggatcttcgaaatggga<br>gccggtactggggctacgactgaagccattgcgtctcttatcgcccgtctgggaatcccagcggtatacaccgtaactgacatctccacctcgtttattgccagagcgcgg<br>aagagattcaagcaataccccttcatggagttccgggcgcatgacatcgagaaggcaccaccaccggagctgcagaacagccagcacgttgtcctagctaccaatg<br>ccgtccatgcaacgcacagtctttcccagtccgctgccagtatccgacaattcctaaggcaggacggcttcttggtgttgctcgaggagacccgccctctgccatgggtg<br>gaccttgtttacggattagtggatagctggtggctgtttgatgacgggcgagagcacgcgctggcgcctccagatgtgtggaagcaggcattgaacaaggctggctatc<br>gcacgattcagtggacggatgggagtcgtcctgaagcaagcatccagcgtcttttcctggcgacattataagaatttgaccctgaagctaccttagtttcgagttcgcaata<br>caagcgaggtaattcaaagccagtagagtagtttgaacgatatatttgcatcgatacggctctgcagcacttccttggaagctatggtttgagtgaggctcggctcatctca<br>cagtcttccgaattgcgtgttccacctcctgtctaa	MHHISGKACVLLFGPLALGLDSASLLKTHKLLIDP<br>DYDSQWVIDVLEELPAYWELLAKEIPALCHCYGA<br>SKLRDLKGWFQTGNIPADAFPLPNILLVPSAIVMQ<br>LVEYQRFAKLANVGRKHSEAYYGNTEAFGLCTGL<br>LSALAVAQSTTQEELHQYGAVAIRLAMVVGATVD<br>AEDTAIRFTACQSQAYASVLFDKRRGASASLLQSL<br>HEVPGLHGRFHHVCHQEVLGRLAEFYDSGPPIS<br>LRVGCAKPEQGEQKLHILALRSILVDQSLWYEAF<br>REIQSLILSSESSRLVCFGQERSIPPSLQQAISSRI<br>VHPTELLVPAKACADPYQSTKISYRGCNGSVNHH<br>AVAVVGMACEVPGATDLDEFAALLKQGKSQHVE<br>VPSERVDFYTPWRETDSKRRWYGNFLDNYDMF<br>DHKFFNKTAREMASTDPQHRLVLQVAYQALQQA<br>GYFRWKDADKNIGCFIAMGLTDYEQNVACHPPN<br>AYSATGNLRSFAAGRISHYFGWTGPALVIDTACSS<br>SAVAVHQACQALKSGECSAALVCGVSLMTSPQW<br>FQNLAGASFLSPTGQCKPFDAAADGYCRGEAVG<br>AVFLKSYQNAVSHHDQILGLIAGSAVSQNRNCTPI<br>TVPNAASLSDLYRSVIEKAGLEPSSISVVEAHGTG<br>TPVGDPAEFDSVRQVFSGQERIGRLHLGSVKGLV<br>GHSEAAAGIVSLIKVILMMHERFIPPQSSFDTINPA<br>IKRSANDQIEITKNLQPWTSDFYAALVNSYGASGS<br>NASLVLTGSPVRLTETEDNGYAEAANVKYPFRIYG<br>FDTPSLQRYVTRFRRYLSTAGQHVSVAGLAHQLA<br>LQSNPTLPYALGFSARTKEELIDKLGIIESGAQAPA<br>AVRTDGARPVILCFGGQISRYIGLDRDIYYHIDIFR<br>QYLDRCNAYCEKLGLGSIFPGIFQRAPVEDIVKLQ<br>TMLFALQYTCAKCWLDCGVSVAAVVGHSFGELTA<br>LCISGAIFLEDALLAIAGRARLIQEYWGADKGSML<br>AVEGDLAVVKELLATANATAEPSPPANIACFNAPRI<br>FTLAGPTHSINVVFDMARGPKYASLVRAKKLEVT<br>NAFHSALVDPLATHLQKLGQNLMFQAPKIHIERAT<br>AEAEFISPGVQSDILLNHMRNPVYFHHAAKRLAQ<br>RYPFSVWVEAGANSSITSMARQALGDLCEHSQH<br>AFQSLNITSGAGVGFNSLVDATTSLWNEGLNIVF<br>WGHCSTEPGRYNPLLLPPYQFAKTRNWMEITRR<br>NIPAVGKADALASAGQNIDDVNRLWKLVISEEDGR<br>RSARFEINSSSQVFRDYLLGHVVAQKAPLATSVL<br>QLQIALEAVASLRSDFQDGTFLPALRGLNSHAPM<br>LLDATRRFWVDAEAQDAGGLTWDLKVNSGASD<br>GSGPTTKHTSGRIVFEKADDPEKLAELSRYKRVM<br>NSTRCLSMLNAGDNDSIMQGPGIYRAFNDVVQY<br>SPAYRGLQKIVGKGAESAGRVVMSPPEGGCIDT<br>GLFDSFCQVAGIYLNCMTDMPEQYMFISTNIERW<br>MRAPTLHSRERWPTSFDVHVCHEYQCDSEVLSD<br>VFVFDAQSGDLFEVILGIRYSRILKERMRQVLHQA<br>IPAPEGSSRLPADEPDPVKDMPHKPVVDGRGKE<br>SSQTGVWTQLQGMLCDLCGLEPDEVRQDSQLG<br>DIGVDSLLGMELAREIESLFQCMLNQDALNEAVD<br>VADLLRCIEETLGVKPITQNGSTEKAAASTFKVSN<br>GDSMDSKGCQNGVEMSFGSTNTGISVNRLPRQ<br>ALSAAKGSIAAVATEVFASTKEATDSFIETYGLADY<br>LDQVLPRSTELVVAHILEALEALGCPVGAVPPGG<br>RLPKVTYLPKHERFMNLLYGLLEKARLIEIHDKGT<br>SLIRTANPLPSTSAEALARELFSDYPEHISDHQLT<br>HYTGKNLSDCLVGQADILQILFGSPECRTIMTNMY<br>SRSPVNLVWIKQMQSFLEGVWTQFRSNDGPVRI<br>FEMGAGTGATTEAIASLIARLGIPAVYTVTDISTSFI<br>ARARKRFKQYPFMEFRAHDIEKAPPPELQNSQH<br>VVLATNAVHATHSLSQSAASIRQFLRQDGFLVLLE<br>ETRPLPWVDLVYGLVDSWWLFDDGREHALAPPD<br>VWKQALNKAGYRTIQWTDGSRPEASIQLSSSQY<br>KRGNSKPHFLGSYGLSEARLISQSSELRVPPPV*

Table S2. Primers used in this study.

Primer	Sequence (5′ to 3′)
asbPKS-F1	TCGAGCTCGGTACCCATGCATCACATCAGTGGAAAAGC
asbPKS-F2	AGTATGCATCACTTGTTAGGGCTAAG
asbPKS-R1	CAAGTGATGCATACTTGGGTCCG
asbPKS--R2	CTACTACAGATCCCCTTAGACAGGAGGTGGAACAC
asbTE-F	TCGAGCTCGGTACCCTATTATTCATGGACCCGGACTCG
asbTE-R	CTACTACAGATCCCCTTAATTGCTTAGTGCGCCTGTTCTTTC
asbPKS(E2052L)-F	TTGCTCGAGTTGACCCGCCCTCTGCCATG
asbPKS(E2052L)-R	AGGGCGGGTCAACTCGAGCAACACCAAG
asbPKS(E2052M)-F	TTGCTCGAGATGACCCGCCCTCTGCCATG
asbPKS(E2052M)-R	AGGGCGGGTCATCTCGAGCAACACCAAG
nvfA-F1	TCGAGCTCGGTACCCATGGAACCTTCGGACACC
nvfA-R1	AGGCCAAAGGGTCATACTCAGTCCAATG
nvfA-F2	GCAAAGACTAGCCATTGGACTGAGTATG
nvfA-R2	CTACTACAGATCCCCAGTCTAGAACTGAACACGATCG
nvfA_F2044L-F	GTTTTGGCTTGACCTTGTGTTCGGGC
nvfA_F2044L-R	AGGTCAAGCCAAAACAGATTCCGAGTC
fncE-F1	TCGAGCTCGGTACCCAGGATGGGCTCATTACCAGAG
fncE-R1	GAATGGAACGCATGGGTGTTGGTC
fncE-F2	CCATGCGTTCCATTCCAGACTGGTC
fncE-R2	CTACTACAGATCCCCAGTAGAACCAGCTAAAGTACAGC
InF-pTAex3_SdaI-F	TGTACTTCTTGTTGCATGCCCCCATCATGGTGTTTTGATC
InF-pTAex3_SdaI-R	GCAGACTCTAGAGTCGAACCGTAAGATACATGAGCTTCGGTG

Table S3. Plasmids constructed in this study and PCR conditions for the amplification of the inserts for the plasmid constructions.

Plasmid	Inserts	Primer 1	Primer 2	PCR Template	Vector
pTAex3-HR-nvfA	1st fragment of *nvfA*<br>2nd fragment of *nvfA*	nvfA-F1<br>nvfA-F2	nvfA-R1<br>nvfA-R2	gDNA	pTAex3-HR digested with *Sma*I
pTAex3-HR-nvfA_F2044L	1st fragment of *nvfA_F2044L*<br>2nd fragment of *nvfA_F2044L*	nvfA-F1<br>nvfA_F2044L-F	nvfA_F2044L-R<br>nvfA-R2	gDNA	pTAex3-HR digested with *Sma*I
pTAex3-HR-fncE	1st fragment of *fncE*<br>2nd fragment of *fncE*	fncE-F1<br>fncE-F2	fncE-R1<br>fncE-R2	gDNA	pTAex3-HR digested with *Sma*I
pTAex3-HR-asbPKS	1st fragment of *asbPKS*<br>2nd fragment of *asbPKS*	asbPKS-F1<br>asbPKS-F2	asbPKS-R1<br>asbPKS-R2	gDNA	pTAex3-HR digested with *Sma*I
pTAex3-HR-asbPKS (E2052M)	1st fragment of *asbPKS* (E2052M)<br>2nd fragment of *asbPKS* (E2052M)	asbPKS-F1<br>asbPKS(E2052M)-F	asbPKS(E2052M)-R<br>asbPKS-R2	gDNA	pTAex3-HR digested with *Sma*I
pTAex3-HR-asbPKS (E2052L)	1st fragment of *asbPKS* (E2052L)<br>2nd fragment of *asbPKS* (E2052L)	asbPKS-F1<br>asbPKS(E2052L)-F	asbPKS(E2052L)-R<br>asbPKS-R2	gDNA	pTAex3-HR digested with *Sma*I
pTAex3-HR-asbTE	*asbTE*	asbTE-F	asbTE-R	gDNA	pTAex3-HR digested with *Sma*I
pTAex3-HR-asbPKS+asbTE	*PamyB-asbTE-TamyB*	InF-pTAex3_SdaI-F	InF-pTAex3_SdaI-R	pTAex3-HR-asbTE	pTAex3-HR-asbPKS digested with *Sda*I
pTAex3-HR-asbPKS (E2052M)+asbTE	*PamyB-asbTE-TamyB*	InF-pTAex3_SdaI-F	InF-pTAex3_SdaI-R	pTAex3-HR-asbTE	pTAex3-HR-asbPKS (E2052M) digested with *Sda*I
pTAex3-HR-asbPKS (E2052L)+asbTE	*PamyB-asbTE-TamyB*	InF-pTAex3_SdaI-F	InF-pTAex3_SdaI-R	pTAex3-HR-asbTE	pTAex3-HR-asbPKS (E2052L) digested with *Sda*I

Table S4. *Aspergillus oryzae* transformants constructed in this study.

Strain	Host strain	Plasmids used for transformation
A. oryzae/nvfA	*A. oryzae* NSAR1	pTAex3-HR-nvfA
A. oryzae/nvfA_F2044L	*A. oryzae* NSAR1	pTAex3-HR-nvfA_F2044L
A. oryzae/fncE	*A. oryzae* NSAR1	pTAex3-HR-fncE
A. oryzae/asbPKS+asbTE	*A. oryzae* NSAR1	pTAex3-HR-asbPKS+asbTE
A. oryzae/asbPKS (E2052M)+asbTE	*A. oryzae* NSAR1	pTAex3-HR-asbPKS (E2052M)+asbTE
A. oryzae/asbPKS(E2052L)+asbTE	*A. oryzae* NSAR1	pTAex3-HR-asbPKS (E2052L)+asbTE

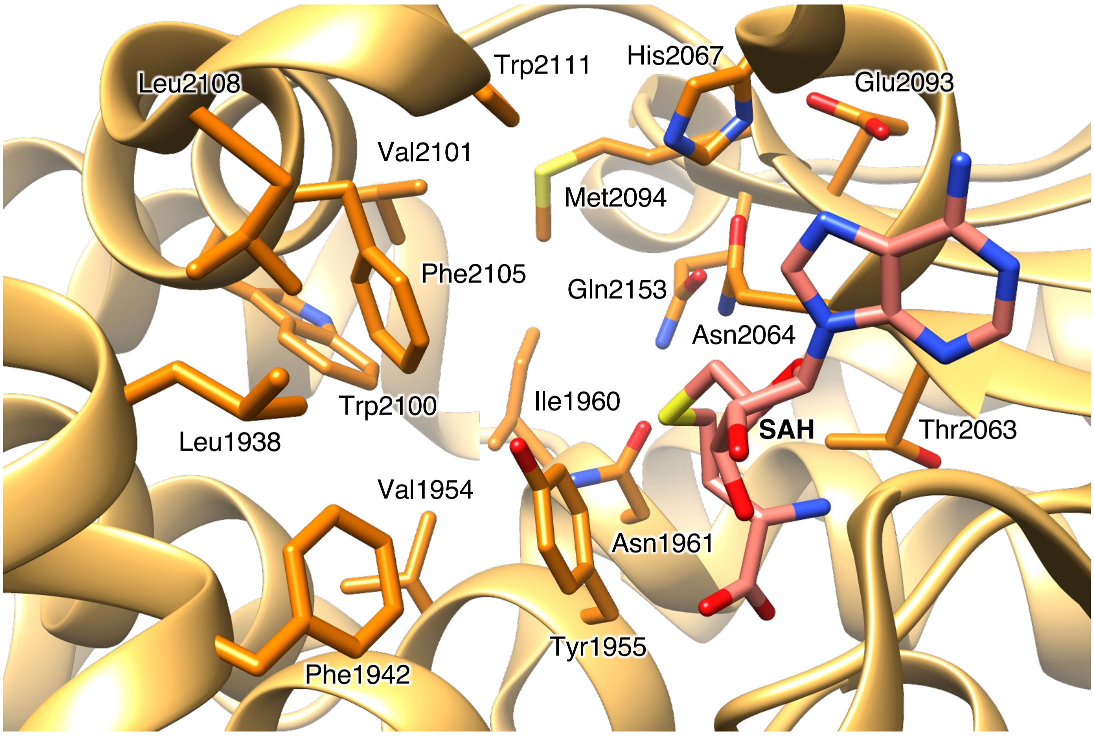

Figure S1. Substrate-binding site of the methyltransferase (MT) domain of CitS (PDB: 5MPT). SAH: *S*-adenosyl-L-homocysteine.

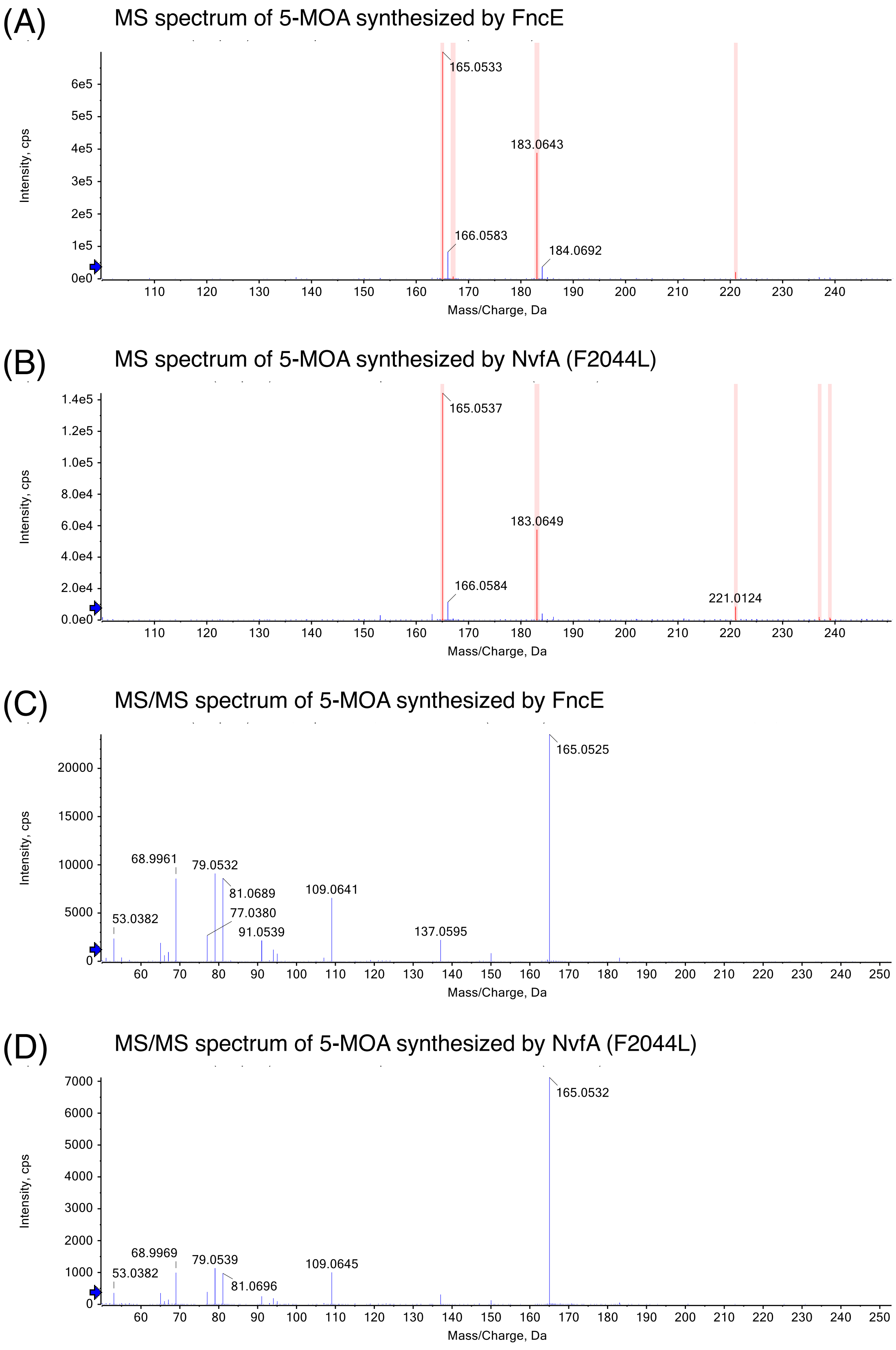

Figure S2. MS and MS/MS spectra of 5-MOA synthesized by FncE and NvfA (F2044L).

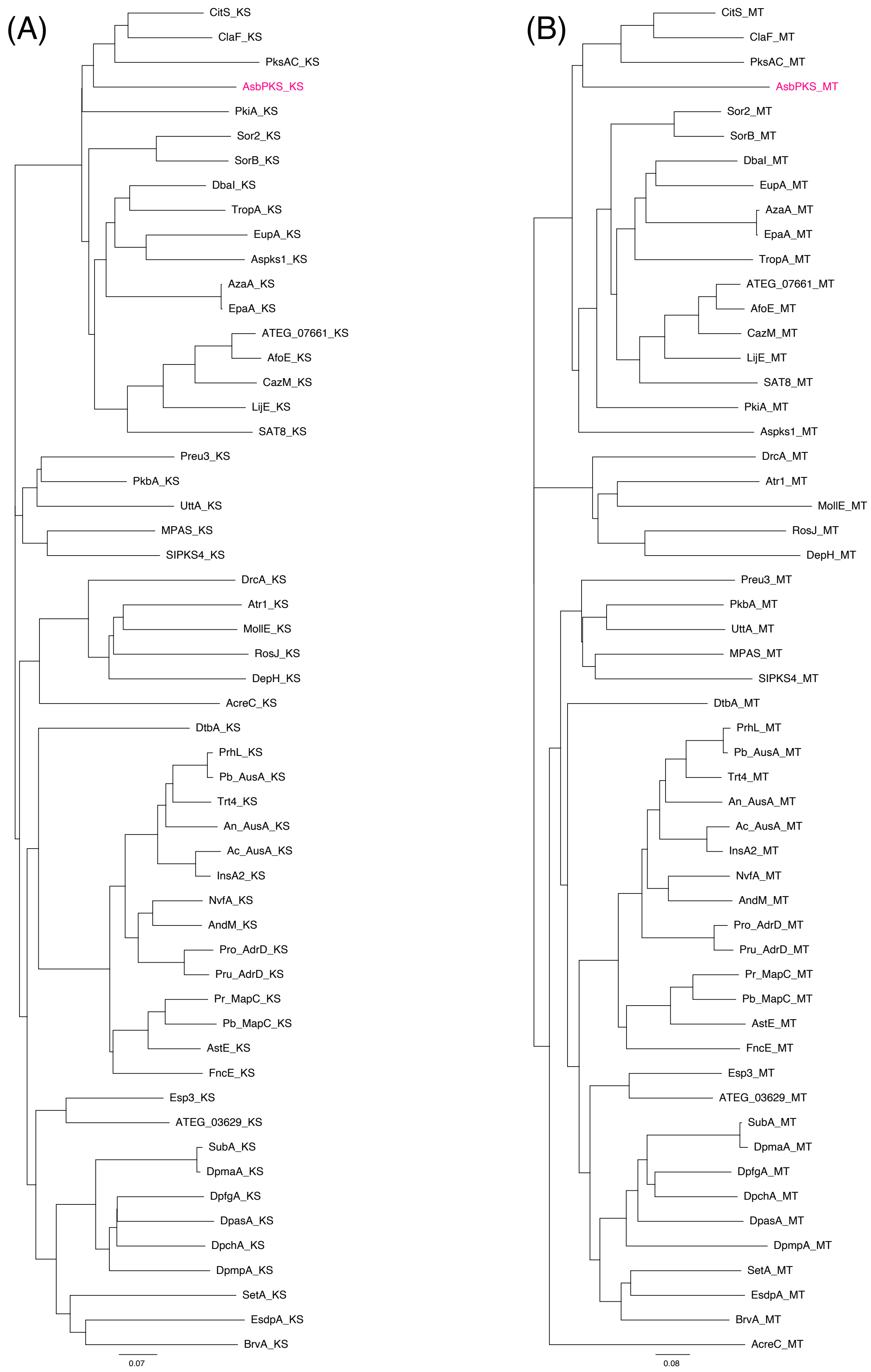

Figure S3. Phylogenetic analysis of the KS and MT domains in known fungal NR-PKSs.

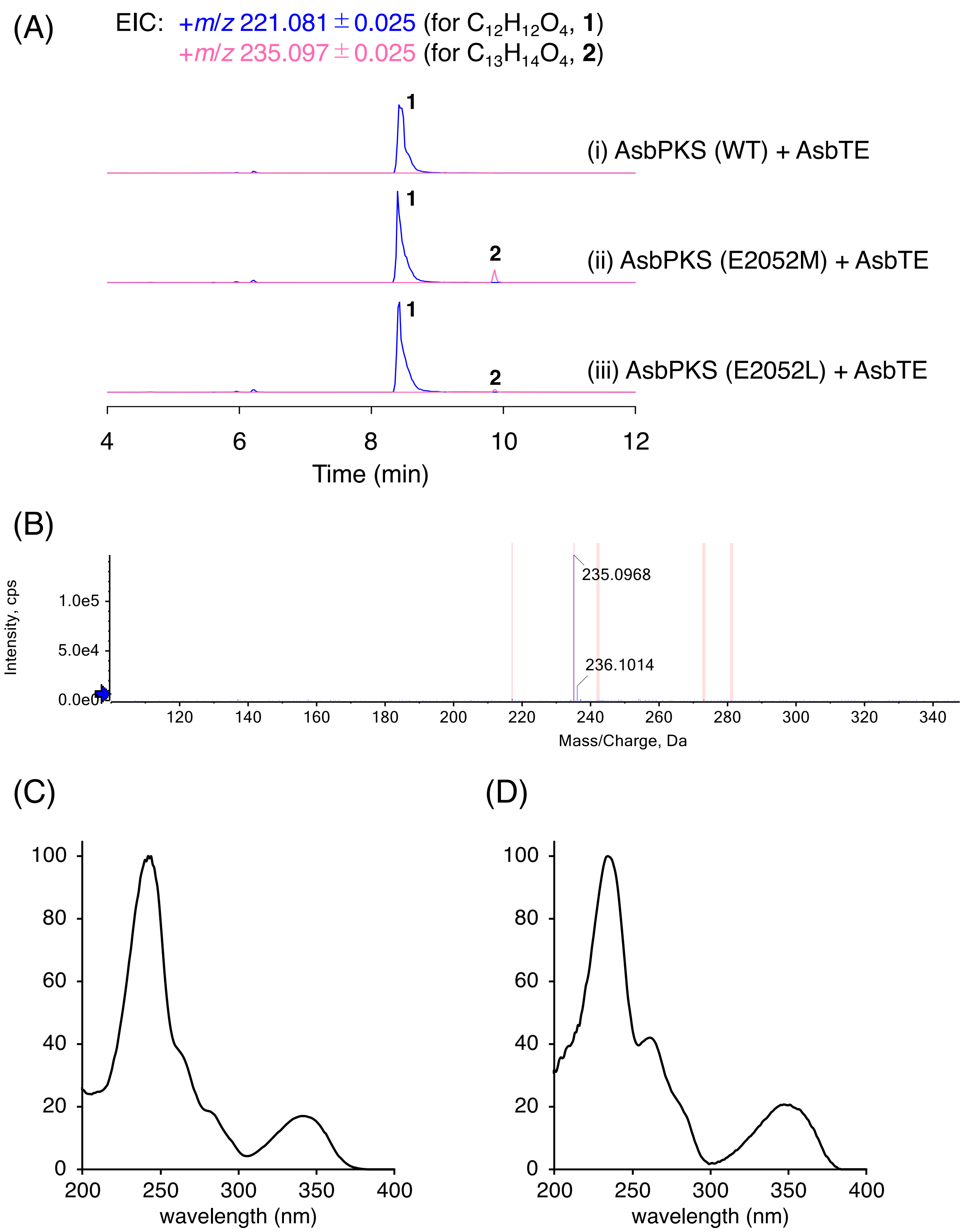

Figure S4. Functional analysis of AsbPKS variants. (A) LC–MS analysis of the metabolites from *A. oryzae* transformants. (B) MS spectrum of **2**. (C,D) UV spectra of (C) **1** and (D) **2**.

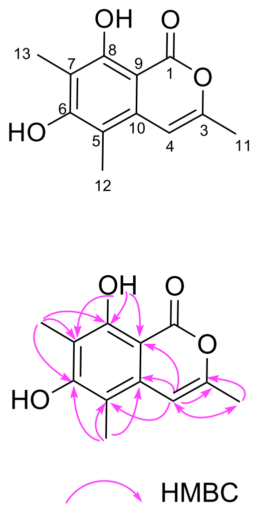

position	δ_C, type	δ_H, mult. (*J* in Hz)
1	166.5, C	
3	152.6, C	
4	101.7, CH	6.63, brs
5	109.0, C	
6	161.2, C	
7	109.8, C	
8	158.1, C	
9	97.7 C	
10	134.0, C	
11	19.0, CH_3	2.24, s
12	10.7, CH_3	2.14, s
13	8.6, CH_3	2.08, s
8-OH		11.4, s

^{1}H NMR: 600 MHz, ^{13}C NMR: 150 MHz (in DMSO-d_6)

Figure S5. NMR data of **1**.

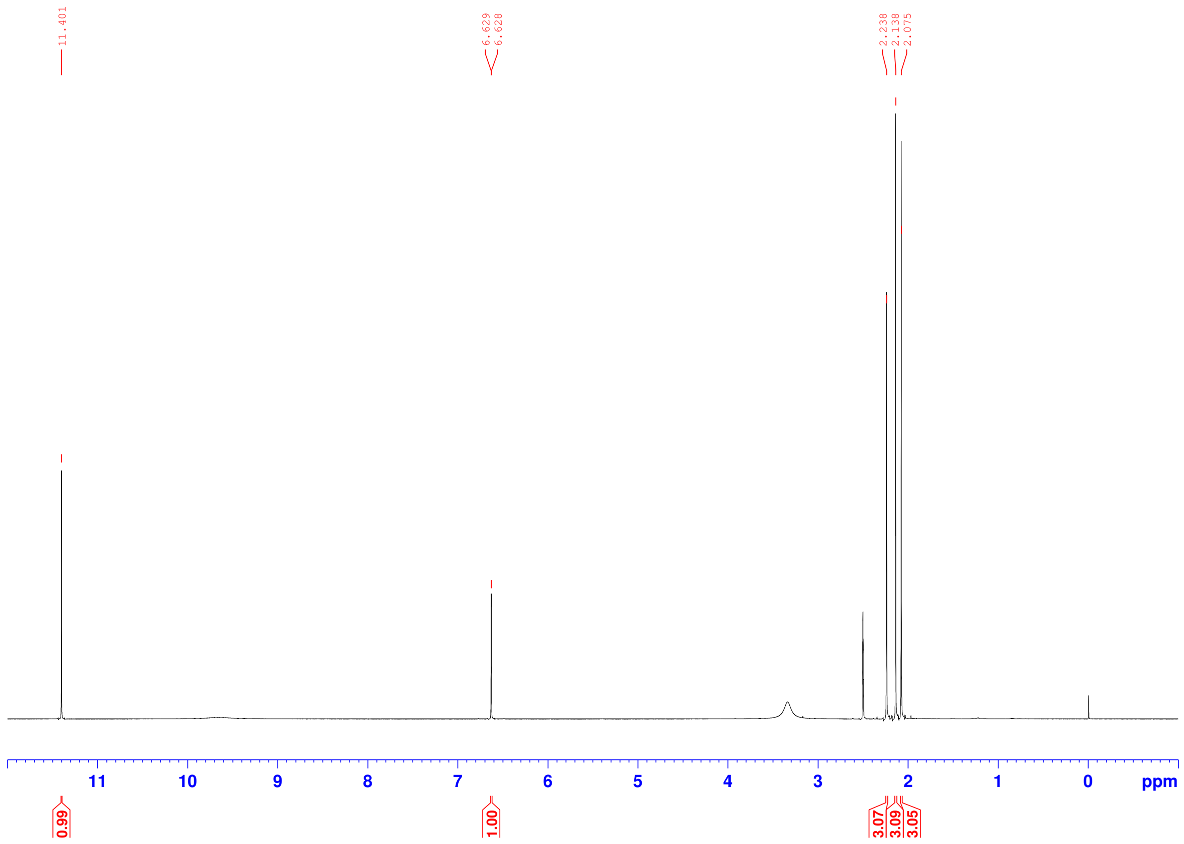

Figure S6. ^{1}H NMR spectrum of **1** in DMSO-d_6 at 600 MHz.

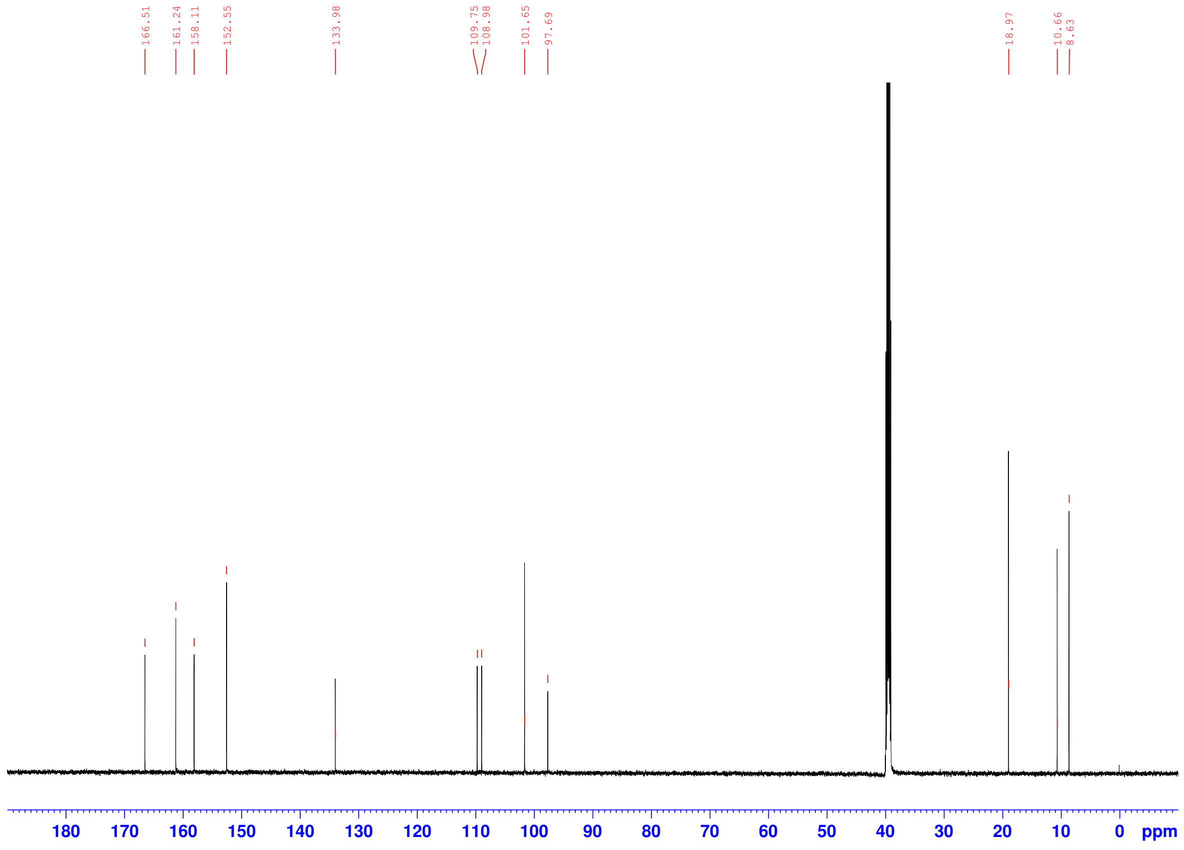

Figure S7. ^{13}C{^{1}H} NMR spectrum of **1** in DMSO-d_6 at 150 MHz.

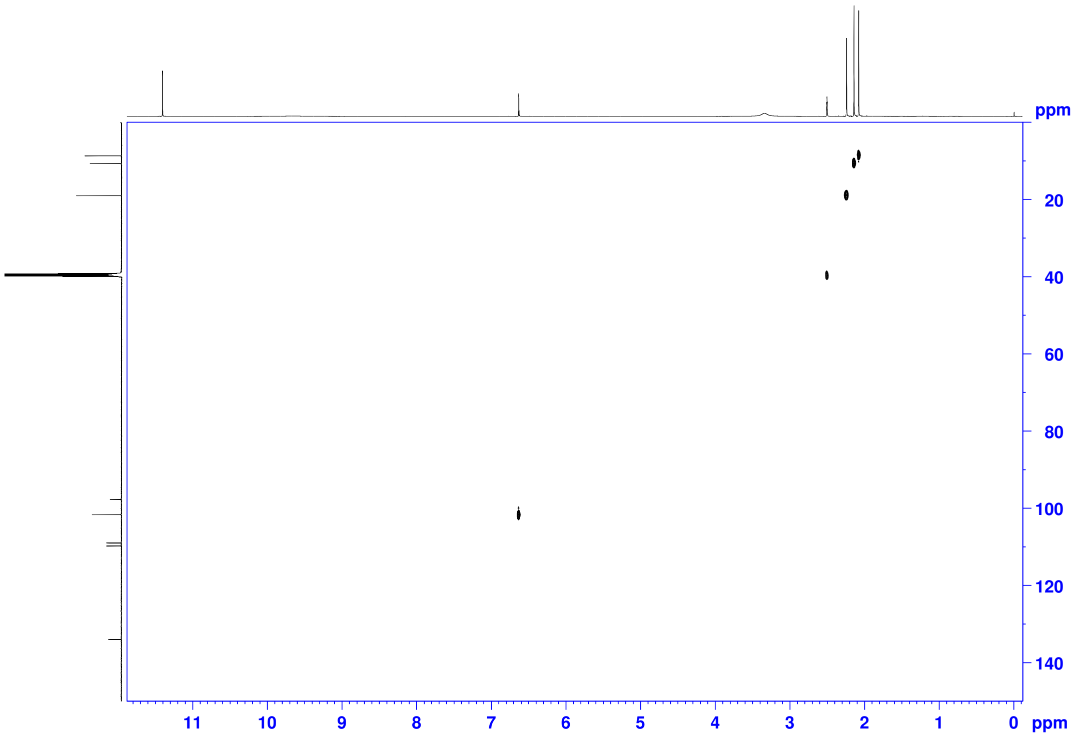

Figure S8. HSQC spectrum of **1** in DMSO-d_6.

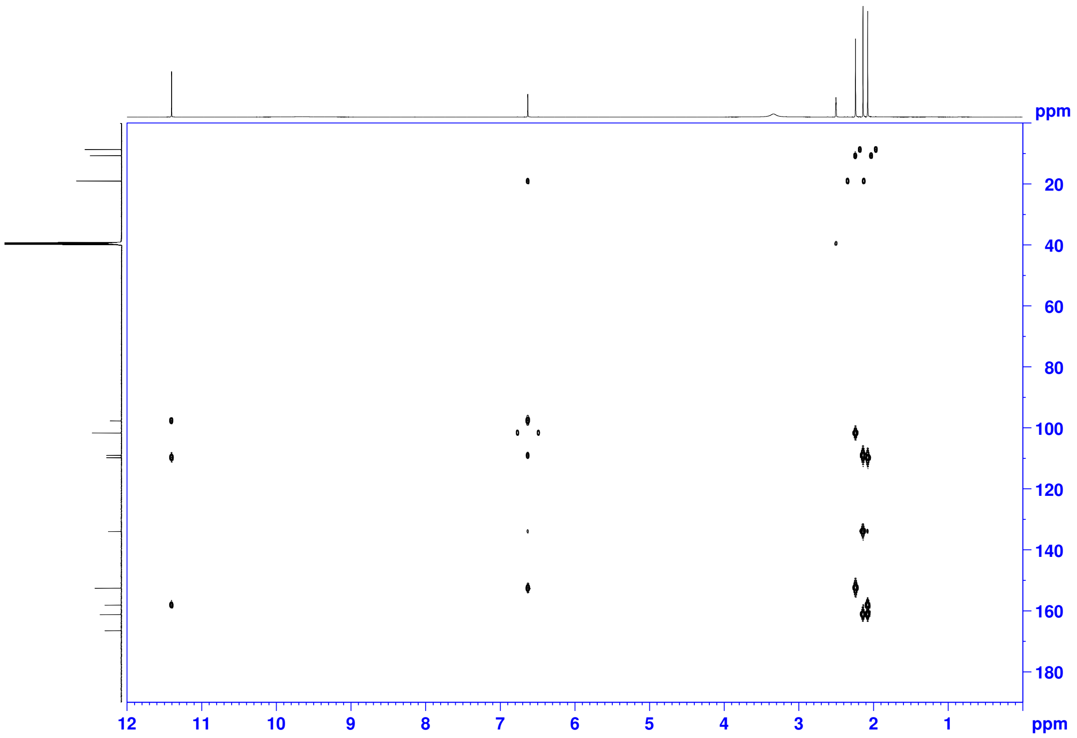

Figure S9. HMBC spectrum of **1** in DMSO-d_6.

Supplementary Data

Sequences of known NR-PKSs with an MT domain.

```
>CitS
MIDSTSHSNLRSKAFIFGPQDLSFDVRSFNKLHSQLQNHQWVLDALASLPKLWDNFAASDQKVQQSNTGKLLENLNAWISSGV
APEEAFPLPNVLLSPLVVIGQLVEYMTFLKAAFPDLGKKHDLPISIKEDTETFGLCTGTLCAFAVACSSNIADIQHYGAVAAR
LAMLVGAIVDTEEVLSDPEGKSVSFSASWNSAEFSDSFTHVLETFPDAYVSVIVDQRRATLTASKKTAPAIIERLKQEGAHVT
SIALSGRFHWKKHQDAVSSLIQFCGLDPDLQLADATKMLLPSRSSSDGQYITTGKLHELALRAILLEQSEWYKTCRISYLSKF
IMDDAAVICFGPERCMPPTLARKLGPRLTYVSEIDISSSRVPGQLLGGTQKLNLTDLPDERIAVIGMACRLPGAEDHEGFWEI
LKTGQSQHREVPEDRFGMATAWREADKRKWYGNFIDNYDTFDHKFFKKSPREMASTDPQHRLMLQVAYQAVEQSGYFRNNGTN
RRIGCFMGVGNVDYEDNIACYPANAYSATGNLKSFLAGKISHHFGWTGPSLTLDTACSSSSVAIHQACRSILSGECNGALAGG
VNVITSPNWYHNLAGASFLSPTGQCKPFDAKGDGYCRGEGVGAVFLKRLSSAIADGDQVFGVIASTKVYQNQNCTAITVPNAI
SLSELFTDVVRQARLEPKDITLVEAHGTGTAVGDPAEYDGIRAVFGGPIRSDVLSLGSVKGLVGHTECASGVVSLIKTLLMIQ
QGFIPPQASFSSINPSLNAKAEEKIEISTRLKPWDAPFRAALINNYGASGSNASMVVTQPPNLTETPSTPLPGKSYPFWISAF
DQQSLQSYVRRLRQFLEKHAADKNLSVANLSFQVACQSNWSLPQALVFSASTKEELNRALASFEKGSTDFPSVQLPDPKPVIL
CFGGQVSTYVGLDQEVYNSTAILRHYLDQCDAMCLSLGLQSIYPAIFQRSPIEDIVQLQTALFAMQYSCAKAWIDSGLKVASV
VGHSFGELIALCVSNAVSLKDAVKMISGRARLIKERWGADKGSMIAVEADLSDVEALLAKVKSQMGSETGLAIACYNASKSFT
LAGPTKDVDHAENLLKNDPDFSGIRYKRLNVTNAFHSVLVDALIDDLESLGQGIRFKEPTIKLERATEQESTSTLNANYVATH
MRKPVFFAQAVKRLSDKFPVAIWLEAGSNSTITAMASRALGTSNSSFQAVNITSEGAFRFLCDTTVKLWKEGQKVSFWAHHRL
QTPMYTPVLLPPYQFEKSRHWMDLKVPPKPEASVQVAEQTAIIEAPKGLTTFVGYQDASQRSVRFRVNVTTEKFNRLLSGHIM
ANAAAVCPGMFQVEIALDALTSLRPEFQARSFIPELHDLRHYQPLVRDESRAVWIEAHCPNAEGLVWNWKLTASDDKGSGSVT
HTSGTITFQAADSVQVKSEFEKLRRLIGRKRCLQLLDSNVADDILQGRNIYRAFTEVIDYKEIYRHVTKIAGRDNESAGRVIK
TYDGETWLDTVLTDCFCQVAGIFVNLMTTKIDLSERGIFICDGIDRWLRAPNAGSNNTPSQVYEVFALHHCESDSKYLSDVFA
FDAREGSLVEVALGISYQKVSISGIRRVLSKGMPAGLQPQVPTSPAAVAAIKTVSPPPVADSPLVDGSSTAVSGTPPTKKAPK
APSVDITGKMREIICNLSGLEPDEVKDDSDLVELGIDSLMSMELAREVDLAFKTTIDVTQLIDVTDFRSLVECMQRILGIDNQ
EDNTYLAEGLNGHEGVVTNGNAYHVNGTNGVVNGNGVLFPELGGSILPKSAILDAFRIAKEATDDFILNGQLGTYYNEVMPRS
TELCVAHIVNAFEQLGCPIRSAAAYQRLERVPYLPKHERFMNLIYGLLEEARLIDINGSEITRTSVPVSTKSVETMLEELLHD
EPLHAAEHKLTSLTGSKFADCITGKEDGLQLIFGSPEGREIVTDVYAKSPINAVWIQQAEFFLEQLVKRLPNTGEPLRILEMG
AGTGGTTVKMLPLLERLGVPVEYTMTDLSSSLIAAARKRFKKYPFMKFKVVNIESPPDPQLVHSQHIILATNCVHATRNLEIS
TRNIHRILRPDGFLLLLEMTEQVPWVDFIFGLLEGWWLFEDGRRHALQPATHWKKILTSVGYGHVDWTEGTRPEANIQRLIIA
LASEPRYDHTPQSLQPPVQVPLTDIAGRQEIIDTYIREYTEDFRALPIPGIQQAVMPAPTGHCVLVTGATGSLGSHVVGYLSR
LPNVHTVVCLNRRSTVPATIRQEEALKVRGISLDDNSRSKLVVLEVETAKPLLGLPVETYQKLVNTATHIVHSAWPMSLTRPI
RGYESQFKVMQNLINLAREVAAWRPVPFKFSFQFISSIGVVGYYPLRYGEIIAPEETMTADSVLPVGYAEAKLVCERMLDETL
HQYPDRFRPMAVRIAQIAGSTSNGHWNPVEHFAFLIKSSQTLKALPDFDGSLSWCPVDDVSATLGELLISNTTPYSIYHIENP
SRQQWRKMVKTLAQSLDIPRDGIIPFDQWIERVRNSSASINDNPARQLLEFFDQHFIRMSCGNLILDTTKTREHSATLRERGP
VGPGLVEKYISAWKTMGFLD
>ClaF
MPSESYPRVNPKVFLFGPQALAFDAKLFTTLQSHLYDSWALDALSDLPIIWESLVKQVPKLQHVEGERLLRELHQGLQTGSLP
DSLFPLPNILLSPLVVIVQLTQYLAFVRSGLPGLGDTDEIPQSVMQTSESLGLCTGILSAFAVSCASSIAKVQQYGAVAVRLS
MLVGALVDAEEASPDTGSPAMSFSMSWNALESRTSVDEVLAEFPEAYISVFVDEKRATVTAPKESAPALLDKLRLSGAHVTEV
ALSGRFHWPKHREDAKQLIAFCDHDPRFQFPDASEIVLPTRLSTGGRLHEIALQEILLKPSEWLSLFGLVQSSHIDAGGANFV
CFGSERCVPPTMIRKLGPLLIHISDVDLSTSALPSELLRSTSASPFDNLPDDQIAVIGMACHVPGAEDLDEYWRILTSGQSQH
TRVPLERFSMKTAFRELEENRKWYGNFLRDYDTFDHKFFKKSAREMSSADPQHRLVLKLAYQAIEQSGYFGASHNSKHVGCYI
GIGNNDYERNIACHPANAYSATGNLRSFAAGKVSHYFGWTGPSLTIDTACSSSGVAIHQACRAILHGECTSALAGGVNVLTSP
EWFQNLAGASFLSPTGQCKPFDARGDGYCRGEGAGVVFLKRLSSAIADGDQVLGVIASTKVYQNQNCTAITVPNSISLAGLFG
DVVEQARLEPQAISVVEAHGTGTPVGDPAEYDAVRRVFGGSIRSDTLSLMSVKGLLGHTEFASGIVSLVKILLMINEGFIPPQ
ASFTSMSPALNAYHEDMINIATQLTPWNVDFRAALINNYGASGSNASMIITQAPKPRSSTSNPSPLSSSATSFPFWLCGIDSQ
SLRAYATKFRRFIHDNADSVKDLTVRNLSFQISRQSNRNLPRALIFSAASRNELEEKLLDYEQGGRSIAEIEVPPPRPVILCF
GGQISTYVGLGKDVYNQATILRSHLDQCNTVCLSLGLGSIYPAIFQRSPILDTVELQTVLFAAQYSCARAWIDSGVKVTAVIG
HSFGELTALCIAGAYSLADALRLISGRARLIRDKWGSDKGSMLALEADLAEVTALLSTSNKPDVSIACYNGPRSFTLAGSTES
VQFIEELARSNQTFFGMKLKKLNVTNAFHSANVDPLISDLEALGREIQFNEPIIQVEAATETRSSPTRGSHFIAQHLRNPVYF
NHAVQRLAEEYPAAIWLEAGSNSTITTMISRALGNSSSPHHFQSVHITSEESLPLLAEATTKLWKEGLNVSFWAHHPMEVSQH
SLVILPPYQFEKARHWMDLKEVPEVKSSIDTTVQPPEPPKGLTTFIGFEDQAKQSARFRVNTTCDKFQQLTSANVALNTTAVT
PGMLQIEISLDAIMNLQPDFKTYQFQPEVQGVSYHNALIDSNSTDLYLDAIAKDDGGLAWRWRLYGTDLGDRVTEFSSGSIVF
LPASDPALKENFERLSRLSGKKRCASLLQGNGADDVLQGRNIYRAFEQVVNYAEPFRRVTKIAGKEDESAGYVSKAYTGETWM
DPVLTECFCQVAGIFINLMTDASDLSKRGVYICDGISRWMHYPGLGSMTSAPDAWEVFAVHHHESETKYVSDVFAFDPRDGSL
IEAILGISYRLVPMDSMRKLLTRGPQQESHFSTAAVSSKSTPVHAPTPTTTVSSTPSSLNSFQEKTIVKNVAKPPGPDISAKM
CEIICNLSGLEPEEIEDDSDLVELGIDSLMAMELVREVDSAFKCTLQNDQLMELTDFASLVSCIRSTLGFDDEESGVGFERDS
SVDTEAYILLEPNEPATNINGANGTVSFDHRDGNAVLSMSTLLDAFREIKWDTDDDIVKGQLGTYSKHVMPRSTELCIVYIVD
AFEQLGCPIRSAAPGQVLTRVPYHPKHEKFMNMIIYGLLEKDARLIDINGSIITRTAVAPPTASADTLLSKLLHDEPVHAAEH
KLAALIGQKFADLITDKEDGLKLIFGNPESREIAADMYSNSPVNTVWIKQLERFFERVLGRLPKDGQPICILEVGGGTGGTTS
RIVPLLAKLGVPVKYTMTDISGSLIAAARKRFKKYPFMDFKPLNMESEPDAKFLQSQHIILATNCIHATRNLSVSLKNLHRIL
RPDGALIMLEMTEQVPWCDFIFGLLEGWWLFEDGRDYVLQPATYWEKVLQSVGYGHVDWTEGELPEARIQRLIIAHASGSRYD
RGPKPPLASIPELTLPDISERRARIDAAVHKYTKDFVAPSQILSPAKLPSLSSGQCVLVTGATGSLGAHIVASLVQRPGIHTV
VCLNRLSTTEATVRQQNSLQMRGISLDPTSLSKLKVIETDTSKPNLGLSPENYQYLIQNVTEIVHSAWPMSLTRPMRTYEPQF
KIARGLIDLAREVAQHRPAPFKFGFQFISSSAVIANYPLLAGTPVVPEQSGTVESVPLTGYAEAKLATERILAETLYRFPDRF
```

HVMAVRIAQITGSTSNGYWNPSEYMPFLIKSSQVLKILPELDGTLSWYPVNDVASVLGELLLSQSTTDLIYHIDNPSRQTWRE
MIAILARALDLGQKSIVPFGQWVNRVRGFRGSIADNPALQLIDFFEHYFVPMSCGGLVLDTTKSSQHSKTLQNQGPIDEDLMM
KYIARWKESGFLNP
>UttA
MVVEGYPDTAVAVIGMACKFPGADSLDEYWQLLDEGRSMCEPAGEDRFATSENRRHQENKSIYNANWVSDIDAFDHKFFRKSS
REAASMDPQQRLLLQVAYHALESSGYFGPVEPRTDVGCYVGVCASDYNDNVAAHPPNAFSTLGTLRAFLTGKISHYFGWTGPS
VSVDTACSSSMVAIDAACNAILAGNCYSAVAGGVSLFTSPYFFQNLSGASFLSATGPTKSFDADADGYCRGEGVGLVVLKKLS
DALADNDNIQGVILSTAVAQSSNLVPITVPYSPSQTSLYRRVLKQAKVSPEEVTYLEAHGTGTRIGDPQEYDGIREVFGCQGR
REKPFHFASVKGNIGHTEGASGVAGLIKTLLMMKRAVIPRQGSFKTLNPKITLEPNLIIPTSSVPWTAKTLIACINNYGAGGS
IAAAVVKEAPKLSIARPSSSSSLASQARYPVFLSAHSPQTLGELASRLRTYINQLPLSSSKNILADFAFNLADQQNRSLSNIV
ATTVSSMSELDDQLRIVSSVPDSAPAQTAVTKPKPIVLAFGGQTNRSVGLNRAVTEASGLFKSYLDECDRILKSFGSNSIYPG
IYESNPRDDIVGLQTMQFALQYASAKTWIDSGLRVDYIVGHSFGQLVGMTVAGILSLEDGLKLVHGRAALMQHSWGPERGEMV
ALDADLKTTQELIATISSPTDKLEIACFNGPKSHVVVGSNAAVDGLIDKLRLSSAVKYKKLNVTHGFHSRFTDAILPALESLA
GTLTYCSPQIPLETCSDSASWPDPPTPQLISQHTRIPVYLGQAISRIVQRLGPSIWLEAGSNSSITSMARRAIGGDDVHSHSF
IPLNLSSASAIGSLADATVHLWRGGHHRVQFWPFHRAERAAYAGTQLLSLPPFPFEKTRHWLDWVEEKPAAAAAAAEAVSAAK
DGADKGEVVSIKELLFSPTWLNASQTSARIRVNPRSEEWRRVVEGHAVLNNPLCPAPLYIELVARAIKILGLGDVSAFHALEI
VAPLGLSQDADLALQLEQQQQSSANGSWSFSFVSQPLNASKQQQNLEPAVHATGAVTIAASGTTSNKAEFDRTARLLPYSRVK
ELLAQADGSSMHGALVYKVFNRVVQYDSYYKGVARVASNKSGQVAADVVLRNDSPPLSWAAEQIVNPLALDNFLQVAGLHVNS
LRDCADSDVFVCTKVDRLAISPSYYSSGGSSTAQPGHGSWGVFSNLNVISDKQVENDVYVFDAATGELVLVVVGAQFSRVLIS
SLSRVLSRANNTATTTTTTTTTEAPKAIPVATSFSETPFTSSPTVRVQQAPPAPSIPAPVKKVRTVRKQKTTPPPPPGIEKDL
RQLLSKITDVPEQEFRGQATLEDLGIDSLMITEIVSEIATAFGVNIPQEDLQDLLTFEALSVYLVRRGARGSSSGSVAVEEEE
YEEVVEEEDEDDAVQNPVVPLSNIAIAAAVVPNKADSQRDENLRLQLAQLVASHLEVAASNFVRATNLADEGLDSLLCMELAS
DIERSFAVRIDVTLLTTESTFGDLADMVFGKGSASGQLLASSLPSSSSGSESLGTSTPAGSTDTDYERISLPDNTNTNTGYIP
QQAFEKIQFDYDQCSRETGFLGFWKNVYPTQARLVLAYVVETFRDLGCPLDVLTPGQTIPLLPILPKHDRLRDVFYEVLRDGQ
IADYTGKEYIRSEKPLDAAPSSQIYAELIRAHPQHAKEHELLHLCGSIMAKLVSGERDPLQLLFGTKKNKELLEEVYSNGPMY
RAMSQLLARFLQQALGGSRSSSGPVRILELGAGTGSTTKWVVDALARAEIPFNYTFTDISPSLVAAGRRKFAQYADSMEFQVL
DIEKEIPSKFFRSFDTILSTNCIHATSNLPNSLTHIYQMLQPGGFVSLVEFTKNMFWFDLVFGLLEGWWLFNDGRQHVLADEA
FWERCMRNVGFQHVAMTEGPSLESNTVRIITGFTSKASEPPATASWIDRKKDETEVETVAFSTTSDGVVLRADIYYPTASSTA
GSAGLSSPKQWPVALLIHGGGHVMLSRKDIRTKQVQHLIRNGILPVSVDYRLCPEVNIVDGPMRDVRDAYVWARTQLPSLKLN
AAPYLQIDASRVAVVGWSTGGTLALTLGWTPDEVVKAPDAILSFYCPTDYEDPCWQAPNFPNKSETEAQNEDYDLLEGVYDQA
ITSYNVPPSKRATGGWMCPSDPRSRIILHMNWRGQMLPVLFNGLPSKKDAPSAESQKQFARLPQPSPSQIAKFSPLAHIATGE
IRSPTYLVHGTEDDLIPWQQSQRTYEALVAAGVEAGISVLEGQPHLFDLFSDGDGKKWERILEAFEFVFRHLGVVPK
>PrhL
MGSLGDLPLNRISVLFGSKYSEIDRSALHIRRYLSTHRAATWLEGAVEDLPSVWQDVTKVWPAGEGIHGEARLQQLSAFLRGE
GLPSNMEDPMNYLLMPITVLRHLVDFHEFKEAGVNCDIKSMQGFCAGYLAAVAACWEKDQSEFSKVVATMVRTAIFIGAAVDL
DELATQRATSIAVRWKTAEAYKPFAATLGRYPGAYMACITDESSVTVTVWEDQAAALVQELERNGLLVKDTRLRGRFHHADHL
SAAQDILKLCQQDSRFQLPDTCPAEELPRSNADGDLPTLKSLLSAAIQSILITQADWNLTVSNTLNSLDSSDAKCILSIGAGQ
FLPRQARSQILNITDSSRGDNLVNGDHDSMTITNGASFVADSINGTAPVPTSIPIAVTGLACRYPQADCVEELWKILEQGLCT
VSRMPESRLKPDRLQRKPDGPFWGNFISRPDAFDHRFFKISAREAESMDPQQRLLLQVAYEAMESAGYCGLRATNLPEDVGCY
VGVGTEDYSENVGSRNATAFSATGTLQAFNSGRVSHHFGWTGPSVTVDTACSSAAVAIHLACQALQTSDCSVAVAGGVNVMTD
PRWSQNLAAASFLSPTGASKAFDANANGYCRGEGAGLVILRPLEAALRDGDPIHAVITGTSVNQGANCSPITVPDSNSQRSLY
MKALSLSGLKPEVVSYVEAHGTGTQVGDPIEFESIRKTFAVPSRTERLYVGSIKDNIGHTETSSGVAGLLKTILMLQKGKIPK
QANFTQLNPKITVNQEDKMSIPTSSILWKTQKRVAMVTNYGAAGSNAAIVLKEPISTPRALCSDEKERLPSVVPFFVAAQTDE
SLRAYCQTLKASLLNGAHLESIAVQDLAFNLARKQNRSMEFSVSFTNSSSLTELHDRLDDVISGRMNIEKKTHTSNPVVLCFG
GQTGNKASISESLVASSALLRLHLDECESACKALGLPSLFPAIFDSSPNNDIVNLHCVLFSIQYATAKAWIDSGLKVDRMIGH
SFGQLTAVCVAGGLSLIDTMQLISTRAHLIRSEWTSEIGVMLSLKGEKNAVRELLDSVPESADLACVNGADSFVAAGSEVAIH
EIQKNAAERGIKSQRLDNTHAFHSRLVDPILPGLAKVASTLNYKPLRIPVEACSESEDDWLLPTWEKIVQHSRKPVYFHQAVH
RTISRIQGPAIWLEAGTMSPIIGMVRRAVDTPSSVQGHVFCPMDLSGPQAESNLAKITSSLWSNGVPVQFWPFHSSQRGYQWI
NLPPYQFAKTSHWIEYDPTAFSYQISKHEEPLTEGLKLVQLLKNEGKVSLFRINDNDPMFRMCTAGHAVVEQNLCPASLYFEL
VARAATTTLPKGTDPTMYHLADLNISAPLVLDMPGSVLLELTQRDSTPGQWAFVLFTREDTLQSVTHATGTISLSPGANNTGI
SSRFSSLKRLLNPAHWDSIATSPSSSGLKRSTVYQAFRRAVTYAEYYRGVESVYALGHEATGRVNLPSSPTKNSPCDPILIDN
FIQVAGIHVNCLSETHDDEVFVCSSVGDVIIGESFVKRDPSVATPWVVYSNYEQESRKKALCDVFVVDEATGSLALCVLAATF
TSVSIQSLRRTLTRLTNKGVSPVPVDIAVAAEVAPAVPAASLITATRASSNGDDLRTVQAMLSELLGIPASEIPASASLADVG
VDSLMNTEVLSEIKNRFQVVITKSELTAIEDVGALVQRIFPGRSTVHIETHAQPAVGITAINGGSKPSSRGSVPASRVGDDLS
GFADKAGELFTASRKSNEHSKATQFLGFCDTVFPQQMELVTAYVVEAFKALGVDLQSLNAGQPIPSVDILPQHSQVMNQLYAV
LEYSGLIERSGTSFCRGHCEVNQNATPVLHQRILNDHPHHTSEHKLLHTTGPRLADCLTGAADPLSLLFQDAQARALMQDVYS
NAPMFKSATMHLAQYLKNLLSQVNSPRPIKILEIGAGTGGTTDYLLKQLSSVAGLCFEYTFTDISPSLVTLARKRFKTFNSIH
YQTLDIEKGPTSEMLGQYDIIVSSNCIHATRSLSTSCSNIQKLLRPQGILCLIELTRNLFWFDLVFGLLEGWWLFNDGRSHAL
AHESFWDRTLRSSGFNWVDWTDNQSEESNILRLIVASPTRPALSLEATMESSDIHEETVVYGRKDDLDLLADIYYPQILDSDG
KSRPVALLIHGGGHIMLSRKDVRHTQVQLLIDMGFLPVSIDYRLCPEVSLLEGPMADACEALAWAQSTLPQLNLQRPDIRPDG
NNVVAVGWSSGGHLAMTLAWTAPARGLRAPSAVLSFYCATDYTDPFWTKPNFPYQGDVSIEDVPTQSPFLGLNDRAITSYNPA
PSKRALGGWMSPSDPRSMIALHMNWTGQTLSVLFNGHKYKSLVAIAGGDDNVILPKPTLSEIQKACPLSHVCAGRYKSPTFII
HGTLDDLIPVEQSQRTHDQMLANGVESELRVVADAPHLFDMSPNLKNNKDAFRAVADGYEFLRSHVRL
>Pb_AusA
MGSLGDLPLNRISVLFGSKYSEIDRSALHIRRYLSTHRAATWLEDAVEDLPSVWQDVTKVWPAGEGIHGEAGLQQLSAFLRGE
ELPLNMEDPMNYLLMPITVLRHLVDFHEFKEAGSDCDIKSMQGFCAGYLAAVAGCWEKDQSEFSKVVATMVRTAIFIGAAVDL
DELATQRATSIAVRWKTAEAYKPFAATLGRYPGAYIACITDESSVTVTVWEDQAAALVQELEGNGLLVKDTRLRGRFHHADHL
SAAQDILKLCQQDTRFQLPNTCPARELPRSNADGDLPTLKSVLSVVIQSILISQADWNLTVSNTLNSLDSSDAKCILSIGAGQ
FLPRQARSQILNAIDSSRGDNLVNGDHDNIAITNGTSFAASSVNGTAPVPTSIPIAVTGLACRYPQADCVEELWKILEQGLCT

VSRMPESRLKPDRLQRKPDGPFWGNFISRPDAFDHRFFKISAREAESMDPQQRLLLQVAYEAMESAGYCGLRATNLPEDVGCY
VGVGTEDYSENVGSRNATAFSATGTLQAFNSGRVSHHFGWTGPSVTVDTACSSAAVAIHLACQALQTSDCSVAVAGGVNVMTD
PRWSQNLAAASFLSPTGASKAFDTNANGYCRGEGAGLVVLRPLEAALRDGDPIHAVITGTSVNQGANCSPITVPDSNSQRSLY
MKALSLSGLKPEVVSYVEAHGTGTQVGDPIEFESIRKTFAVPSRTERLYVGSIKDNIGHTETSSGVAGLLKTILMLQKGKIPK
QANFTQLNPKIIVNQEDQMSIPTSLIRWETQKRVAMVTNYGAAGSNAAIVLKEPIPTPTALCSDEKERLLSAVPFFVAAQTEE
SLREYCQALKARLLNGAHLESIAVQDLAFNLARKQNRSMEFSVSFTNSSSVTELRERLDDVISGRMNIVKKTHTSNPVVLCFG
GQTGNKASISESLVASSALIRLHLDECESACKALGLPSLFPAIFDSSPNKDIVNLHCVLFSIQYATAKAWIDSGLEVDRMIGH
SFGQLTAVCVAGGLSLIDTMRLISTRAHLIRSEWTSEIGVMLSLKGEKNAVRDLLDSVPDSADLACVNGADSFVAAGSEVAIH
EIEKNAAERGIKSQRLDNTHAFHSRLVDPILPGLAKVASSLNYKPLRIPVEACSESKEDWMLPTWEKIVQHSRKPVYFHQAVH
RTISRIQGPAIWLEAGTMSPIIGMVRRAVDTPSSARGHVFCPMDLSGPQAESNLAKVTSSLWSNGVPVQFWPFHGSQRGYQWI
NLPPYQFAKTSHWIEYDPTAFSYQMSKQEKPPIEDLKLVQLLKNEGKVSLFRINDNDPMFRMCTAGHAVVEQNLCPASLYFEL
VVRAAITTLPKGTDPTMYHLADLNISAPLVLDMPGSVLLELTQRDSTPGQWTFVLFTREDTLQSVTHATGTISLSPGADNTGI
SSRFSSLKRLLNPAHWDSIATSPSSSGLKRSTVYQAFRRAVTYAEYYRGVESVYALGHEATGRVHLPSSPTKNSPCDPILIDN
FLQVAGIHVNCLSETHDDEVFVCSSVGDVIIGESFVKRDPSVAAPWVVYSNYEQESKKKALCDVFVVDEATGSLALCVLAATF
TSVSIQSLRRTLTRLTNKGVSPVPVDIAVAAEVTPAVPAASSITATRASSNGDDLRTVQAMLSELLGIPTSEIPASASLADVG
VDSLMNTEVLSEIKNRFQVVITKSELTAIEDVGALVQRIFPGRSTVHIENHDQPAVGITAINGGSKPSSGGPVPASKVGDDLS
GFADKAGELFTASRKSNEHSEATKFLGFCDTVFPQQMELVTAYVVEAFKVLGVDLQSLKAGQPIPSVDILPQHGQVMNQLYAV
LEYSGLVDRSGTSICRGHCEVNQDATAVLHQKILNDHPQHTSEHKLLHTTGPRLADCLTGAADPLSLLFQDAQARALMQDVYS
NAPMFKSATMHLAQYLKNLLRQVNSPRPIKILEIGAGTGGTTDYLLKQLSSVAGLRFEYTFTDISPSLVTLARKRFKTFNFIH
YQTLDIEKGPASEMLGQYDIIVSSNCIHATRSLSTSCSNIQKLLRPHGILCLIELTRNLFWFDLVFGLLEGWWLFNDGRSHAL
AHESFWDQTLRSSGFNWVDWTDNQSEESNILRLIVASPTRPALSLEATTESSAIHEETVVYGRKDGLDLLADIHYPQILDSEG
KNRPVALLIHGGGHIMLSRKDVRPPQVKLLIDMGFLPVSIDYRLCPEVSLLEGPMADACEALAWAQNTLPQLNLQRPDIRPDG
NNVVAVGWSSGGHLAMTLAWTAPARGLRAPEAVLSFYCATDYTDPFWTKPNFPYQGDVSIEDVPTQSPFLGINDRAITSYNPA
PRKRALGGWMSPTDPRSRIALHMNWTGQTLTVLFNGHKYKSLVAIAGGDDNVILPKPTLSEIQKACPLSHVYAGQYKTPTFII
HGTLDDLIPVEQSQRTHDQMLANGVESELRVVADAPHLFDMSPNLKNNKDACRAVADGYEFLRSHVGL
>Trt4
MGSLQDAHPHRVSVLFGPKCPKTDRSVLHIRRYLSSHRNTGWLEDAVQALPSVWHDVTKVWPAAEKIPGFCVGYLAAVAACWE
TDQTEFPKAVATMLRIAVCIGAVVDLDELEKQRATSMAVRWKTSADYKLLTALLSRYPGAYIACVTDESAATVTIWESQAAAL
VKELESNGLVVKSTQLRGRFHHSDHTSVVQEFLKLCQEDNRFHLPNGNPAVGLPRSNIDGEVPTLQSLLSVANESILISQANW
NLTVSATISSLQLTDAKSIVSIGAGQCIPRKARGRILHTVEPPDSHHNTNTTQDSDVTTNASPLTAGYVNGTGPAATATTVPI
AVTGMACRYPQADSMEELWKILEQGHCTVSPMPKNRFKLDELQREPKGPFWGNFLSRPDTFDHRFFKISAREAESMDPQQRLL
LQVAYEAIESAGYCGLRASQLPQDVGCYVGVGTEDYSENVASRNATAFSATGTLQAFNSGRVSHYFGWTGPSVTIDTACSSAA
VAIHLACQALQTNDCSMAVAGGVNVMTDPRWSQNLAAASFLSPTGASKAFDADANGYCRGEGAGLVVLRPLEAALRDGDPIHA
VITGTSVNQGANCSPITVPDSNSQTTLYLKALSISGIKPDVVTYVEAHGTGTQVGDPIEFQSIRKTFAVPHRTERLYVGSIKD
NIGHTETSSGVAGMLKTILMLQKRRIPKQANFTRLNPMITLQKEDQIFIPVESTDWKAEKRVAMVTNYGAAGSNAAIVLQEPT
CTSRTPISGYREYLPSVIPVFVAARTEESIREYCKALQTAFLEAPQVNNVEVQDIAFNLARKQNRDMEYSVAFTTASGNDAEL
RERLEDIVSGRTRIEKKCQAAHPVVLCFGGQTGNTASISQNLVQSSELLRFHLYATAKAWLDSGLRVDRMIGHSFGQLTAVCV
AGGLSLLDAMRLISSRAQLIRSEWKSDTGLMLSVRGEKETVQALLDAVSNAADIACVNGPESFVVAGDEATIHKMENIAVERG
MKLRMQRLKNTHAFHSKLVDSILPGLTKIASTLNYRPLRIPVEPCSELADDWSLPTGDKIVQHSRKAVYFHNAIRRTISHMDS
PCIWLEAGSASPIIRMVRRAVDASPSPRDHVYCPIDLSGPQAEENFAKVFSSLWSKGVQVQFWPFHGSQTGYRWINLPPYQFA
KTSHWIDYDPNAFYSDPPKREAGRTDEPSLVKLLNNDGNVYLFGVNVNDPLFRMCTAGHAVVDQNLCPASLYFELVVRGAVAV
LPLENDPTMYHIAGLDISAPLVLDMPGSVFLELTQRGSGPGQFTFVLFTRDGNQDSVAHATGKISISSEANDSGISSRFGSLR
RLVNPSRWGFIATSPSSSGLKRSTVYQAFRRVVNYADYYRGVEEVYAVGHEATGRVLLPSSPTNKASCDPILIDNFIQVAGIH
VNCLSETLDEEVFVCSSVGDVIIGELFVRRDPGVSVPWIVYSSSERESMKKSLCDIFVVDEATGSLALCILSATFTCVSIQSL
KRTLTRLNNKALASTGVDVVVPAVAVAPAAPAASAAMPDSSRSEDGLRVVQAMLSELLGISAGEIPASAALGDVGVDSLMSTE
VLSEINKRFKVVITNSELTAIADVSGLVQRIFPGGSVAHVETHSQPPDKIGITTGDRMPPPRVPPPTVIQEQSLPGFVDKARE
LFAASRTSNEYRQKTRFLGFCDSVFPQQMELVTVYVVAALKALNVDLQSLRFGQAVPSVEVLPQHGKVMNQLFAVLEYSGLVE
RRGTGMIRGHRAVNKSTATILHKKILSEHPQHASEHKLLHTTGSRLADCLIGAADPLSLLFQDAQARALMQDVYSNAPMFKSA
TMHLAQFLKDLLGQRCFQRRISILEIGAGTGGTTDYILKQLSSVAGLTFEYTFTDISSSLVTLARKRFKTYHFMHYATLDIEQ
DPAPELQGQYDIIISSNCIHATRSLATSCSNIQKLLRPQGILCLIELTRNLFWFDLVFGLLEGWWLFNDGRSHALAHERLWDQ
TLRQAGFNWVDWTDNDSEESKILRMIVASPSQPVLCSPGEAKSNAVAEETLVYSRKDGLELCADIYYPHGLDGEDRKRPVAIL
IHGGGHIMLSRKDVRPMQVKMLLDMGFLPVSIDYRLCPEVPLLEGPMVDACDALAWARHELPQLQLKRPDIRPDGGNVVAVGW
SSGGHLAMTLAWTAPARSVRAPEAILSFYSPTDYTDPFWTKPNFPYQESVCMSDVPTSDPLLALHDTAITSYNPAPGKGVLGG
WMSPSDPRSRIALHMNWTGQTLPVLFYGCKYKSLAAAAKGDEVLLPAPHMSEVEKACPLSQVRAGRYKTPTFLIHGTLDDLIP
VQQAQRIHQQMLVCGVESVLRIVSDGLHLFDIIPHLKENKDASQAVLDGLAPHIPTDEYPNPPPKLPKMDTSYPKCLYLISGV
PVKALEIMSCYVFHR
>An_AusA
MGSLDDNTLQQVSVLFGPKYPEVELPAGHIRRYLSNQRNANWLHDAIRDLPSVWHDILRLWPAAEKLHGDARLRQLSAFLGGG
TLRPDMAEPMNFLLVPATVLRHLVDFLELKEDKNYDVCDIQGFCVGFLAAIAAACWSDNEDEFGKVVSTVLRLAVYIGAAVDL
DELCEQPARSIAVRWRTAQEHKLLTEVLTRYQGAYISCVTDENAVTVTVWDSQSVSFAKELEKHGLSVKTTTLRGRFHHSNHT
QAVEDILQSCERNSRLCLPSKCHKRSLPRSNINGRVCEADSLFTVAVESILTTQANWKITVTATLDNMGQSDARSIIPIGAGQ
FVPRHARCRMLNIVEFNKGEHINGRRKMQSATALDVGVNVTAPETTAVPIAVTGMACRYPQADSVEELWRILDLGQCTVSPMP
NSRLKSGSLQREPKGPFFGNYLARPDAFDHRFFGISAREAESMDPQQRVLLQVAYEAMESAGYCGLRRSKLPDDIGCYVGVGC
DDYSENVGSRNATAFSATGTLQAFNSGRISHYFGWSGPSVTVDTACSSAAVAIHLACQAIRTNDCAIAVAGGVNIMTDPRWSQ
NLAGASFLSPTGASKAFDADANGYCRGEGAGLLVLRPLEAALRDGDPIHAVITGTSVNQGANCSPITVPDSNSQRSLYLKALS
LSGLTPDVVGYVEAHGTGTQVGDPIEFESIRKTFSGPNRATKLYVGSIKDNIGHTETSSGVAGMLKTILMIQKRRIPKQANFR
RLNPRITLNERNHIEIPTQSIDWEAEKRVAMVTNYGAAGSNAAIVLREPASTPATSNSAHRETLPSHVPFYVSARTEESLRSY
CEALQSTIREVAQSGTNTVQHIAYNLARKQNRDMEHFVTFPAAAGEPSELMTRLGSIASAHTQVERRSQSFHPVIICFGGQTG
DTASISRNLFESCELLRFHVDECENACNALDLPSLFPAIVSPFPNKDIVNLHCVLFSIQYATAKAWLDSGLQVTRMIGHSFGQ

LTALCVAGGLSLIDGMRLVATRAQLIQKHWGPHTGVMLSLRASKEKVQALLDAASGHADLACLNGPDNFVVAGDEESIRRIEI
IATEKGMHVELKRLKNTHAFHSRLVDAILPGLSEVANTLTFRQLDIPVEACAEQEDDWLWVTGDKIVQHSRKPVFFHDAVERT
LSRVDGPCVWLEAGTASPVINMVRRVVEASRPLKSHVYLPTDLSGAQAQANLAKVTCTLWSKAVPVQFWPFHPSETGYRWINL
PPYQFAKTSHWIEYNPDAFRSPPQVPDQENVQEASLVRLLRQDGKEALFTINNKDNVFRMCTAGHAVANQNLCPASLYFELVV
QAALLVSSTATKPTMYHIESLNICSPLVLGMPGAVLLQLTQQDESHGQWSFVLSTRDGLQDAVTHATGRVSLQAAGSNTGICA
RLSSLQRLLNLASWNSIATSPSSSGLKRSTVYQAFARAVNYADYYRGVEEVYAVGHEATGRVILPSSPTKCNPCDPILIDNFI
QVAGIHVNCLSETHDDEVFVCSSVGDVLIGESFVRRDTAATVPWAVYSNYEPESKKKIVCDVFVLDHTTGALAVCMLSATFTG
VSIQSLKRTLNRLSNHTARPTEAEQVSINVAAEATALSSTPVAHVSSSDGDLLAVQTMLGELLGISADELSAAAALGDIGVDS
LMSTEVLTEINKRFGVAISNAELTQIPDVGGLVQRIFPGHSVVRIKTHSQGAVETEITITDREPKSISVDLAPVCDTSPTAFV
DKASKLFATTRTSAEFSRKTRFAGFCDTVFPQQMELVTSYVVEAFHALGADLASLTPGQVVPPVKILPQHGKVMNQLVAVLEY
SDLIERRESEIIRSQQPVGTVPSLILYKKILNKHAQHASEHKLLHTTGSRLAECLSGKADPLSLLFQNAEARALMTDVYSNAP
MFKSATIQLAQYLKDLLFNLGTQREIKVLEIGAGTGGTTNYLVQELAAVPGLRFQYTFTDISSSLVTLARKRFKAYDFMRYTT
LDIENDPSPELQGQYDIIISTNCIHATRNLITSCTNIRRLLRPEGILCLIELTRNLFWFDLVFGLLEGWWLFNDGRSHALAHE
RLWDHNLRQAGFNWVDWTDNDSAESDILRLIVASSTQPFYALEGDDECEADCNTVQEQTVLYNTRDGLELFADIYYPEKTDRS
GAKRPIALLIHGGGHIMLSRKEIHHEQVRMLFDMGFLPVSIDYRLCPEVSLLDGPMQDACDALAWARNKLPQLQLQRRDILPD
GNNVVAVGWSTGGHLAMTLAWTAPARGVSAPEAILSFYSPTDYTDPFWSKPNFPYRVDVSTSDIQTGNPLDALQDAPISGYNP
PPSKRALGGWMAPSDPRSRIALYMNWTGQTLPVLFYGCNYRARAAESGQDYEVVLPEPILSEVQKVCPFSQISAGSYRAPTFL
IHGTLDDLIPVQQAQRTHDKMQACGVDSDLRIVRDGLHLFDLEANFAGNQHAFQAVVDGYEFLRRHVGL
>Ac_AusA
MGPQTPSSEPPRPVTVFFGPVYPELTQSSSRIRQYLADKASAGWLDDTLQGLPSTWQDIMRQWPALKKIPGESLLRQLTQYLC
RESSCPVGDTLNLLLVPVTVLRHIVEFQQLKDEKKHLEIRDVQGFCVGVLAAITASWEHDDAEFPKVVSTVLRVAVCIGALVD
LDEVNGSSSKSMAVRWKTKCEYRHLGQVLERYKGYIACMIKTNGATVTVPTANYLSLAEELESHGISVKNLPLRGRFHTADHI
PAMKQLLALCARDARFQLPIKKNLPLLPRSNVDGTRLPSNVLMAAAVESILAKQANWMLTVAEALNSEGPPDEKHAVVIGAGQ
IIPQRSLLASVEHIGDQMAPTDTSLHRPSPTIDIRPTAQCNGTSPETSTQAVSAPIAVTGFACRYPQADSVEALWTLLERGQC
TVSSMPNHRLKADSLQRQPRGPFWGNYLESPESFDHRFFGVSAREAESMDPQQRLLLQVAYEAIESATYCGLRATKLPDDVGC
YIGVGSDDYSQNVGSRDATAFSATGTLQAFNSGRISHFFGWSGPSITVDTACSSAAVAIHLACRALQANDCSIAVAGGVNVMT
DPRWSQNLAAASFLSPTGASKAFDAAADGYCRGEGAGLLVLRPLDAALRDGDPIHAVITGTCVNQGANCSPITVPDSNSQRSL
YMKALAQSGLHPDAVCYVEAHGTGTQVGDPIEYESIRCTFGGPQRTETVYIGSIKDNIGHTETSSGAAGMVKTILMIQKRRIP
KQANFSCLNPRIVTHERDQIAIPTQSLEWKAAKRVALVTNYGAAGSNAVIVVKEPIKPGVDRSTWPARVPFIITAKTEESLRE
YCRELQHTLLAQQQGSGSATHHLAYNLAAKQNRCLEYQLSFSCEPAEVATRLQDIAAGRSKPTRCTAPSPPVVLCFGGQTGDT
ASISPTLVENCDILRSHLTQCDEVCHALGLPGLFPTIFSPEPRTDLVSLHCILFSIQYASAKAWLDCGLVVDRMIGHSFGQLT
AICVAGGLSLIDGLRLVSKRAALIQEKWGPERGVMLSLKVTKTQVQELLCAASGTVDVACFNGPQSFVLAGNEKSIAQVETLC
VQRGLEHHKKRLRNTHAFHSRLVEPLLPELSQVADTLDYSPLRIPVEACSEEPDHWARLTASKVVRHSREPVYFHPAVQRTLR
HIPGPCVWLEAGSASPIVGMVRRVVKASGPGGEHTYLPIDLQESTAECNLADVTKVLWSKGVPVQFWAFHGSPAGYKWINLPP
YQFSKTRHWIDYDPYAFHPTGALSEEEKHDDGLLQLVERDANGCLFRINNQDPAYRMCTEGHAVVDQNLCPASLYVEIVVRGA
MTLSANGQAAAMAHIEALSISAPLVVDMPGSVCLSVTQIANNNDGGWMFSLFSQDGDRTPITHATGKVLLDPQAAGSAASARF
HSLKRLLDPGQFDSIPKSPSSNGLKRATVYQAFRRAVNYADYYRGVEEVYAVGHRAAGRVLLPSSPTRMAACDPILMDNFLQV
AGIHVNCLSETDEDEVFVCSSVGEVSLGSRYLNRDAATPQAWTVYSTYERESGKRVTCDVFALDEDRMLAVTIMSATFTSVSI
QSLKRTLSRLNGHSSSLGQHEPQLQEKLAPAAHITVSDDHHLRAVQSMLGDLLGVSPGELPGNAPLAEIGVDSLMSTEVLAEV
DKRFGVNITNTELADIADVRGLAHRISPSSSVVHVETSKESSVANDISVGGQQPIIESPPVTHQEDSPRFADRAITAFAATRG
STKYIDLTQFSAFCTSVYPQQMRLVTAYVAEAFQALGANLESMLPGQSIPSLAILPQHNQVLGQLIGVLEYAGLVEQKSTGLF
RTGKPVDVGPSAVLHQTILGDYPQHASEHKLLRTTGARLAECLTGTADPLSLLFQDAQARALMQDVYSNAPMFKAATMQLAQY
LQDLLLDSGCDREIEILEIGAGTGGTTAFLVSQLAAMPGVKFKYTFTDLSSSLVTLARKRFGSYSFMRYSTLDIEKMPRDELL
GKYDIILSSNCIHATRSLAASCMHIRKMLRPHGLLCLIELTRNLPWFDLVFGLLGGWWLFNDGRSHALANESLWDTRLREAGF
NWVDWTDNPLEESDILRLIVASPTMASPPLPVKQPITPARVETVRYGERAGVQLMADIYYPHTVDAKGTKRPIALLIHGGGHI
MLSRKDIRPYQVDLLLDAGFLPVSIDYRLCPEVSLLEGPMPDVRDALAWARADLPRQSLSRTDIQPDGDRVVAVGWSTGGHLA
MTLSWTAPEHGILPPQAILAFYSPTDYTDPFWTTPNFPYAGAVSEEDTKLTRPLDALRDSPITAYNPPANKHALGGWMAPSDP
RSQIALYMNWTGQALPVLFNGCNYKKLAAAKGPTTSEVILPAPPLADVQRACPLSQIVAGRYKTPTFLIHGGLDDLIPVQQAE
RTQDAMRAAGVESTLRVVQGGLHLFDLGIDISAVDEDGPRAVREGYEFLRQHVAV
>NvfA
MEPSDTERCDVGILFGPQSSDMDEALSCIRSYVLEQPAVRYLVDLVLELPSLWPEIKNAWPALSQVPGEEQLVALGRFFNGGP
FPASDEAMNVITTPVTVIRHIVEFYKVKETMKGFQARDVQGFCVGFLAATAVAASCDETAFRALVSKIIRLAVCIGGLVDLDE
LAVHRARSMAVRWDGEEDYDRLEQVLAAHPEAYIACVTDANRATLTVPKSLAPQMIQDLANHGLSVREIRLCGRFHHPDHTAA
VEQMSRLCERDCRFQLPDASSLSLPLRSNINGEVIRTGQLHTIALQSILCFRSQWLITVTAALASITMTDESIRLVSIGPHQC
VPRMAQSKLIRTVTSSPVDGCYEAINGTGAAPVRPIAVTGMACRYPQANSVEELWEMLELGKCAVKPLPNDRLKMVELLREPK
GPYWGHYLEEPDMFDHRFFGISAREAATMDPQQRLLLQVAYEAMESAGYCGLRSSQIPRDVGCYVGVGSDDYTDNVGSHHANA
YSAPGTLQAFNTGRISHYFGWSGPSVVVDTACSSAAVAIHLACQALRTKDCSVAIAGGVNVMTSPKVTQNLAAASFLSPTGAS
KAFDADADGYCRGEGAGLVVLRPLEDAISDVDPILAVITGTAVNQGSNCSPITVPVSESQMSLYGKSLAASGIAPEDVTYVEA
HGTGTQVGDPIEFDSIRRMFGGRHRSEELYVGSIKDNIGHTETSSGVAGLVKTILMMQKGRIPKQANFSRLNPKIPAPEGDRI
VIPKQSTDWKSARRVAMVTNYGAAGSNAAIVLRQHTITTNTGSSWLSDVPVFVAAKSPESLRSYCYKMQAFLRQTAGLLGCTM
RDITYNLAIKQNRDLDFLVSFPTPSQDPMTLLSQLESVAAGVTDLQQRPAQAPSVILCFGGQNGNTAHISQDLFAGCHLLQAH
LADCEKICQSMGLPSLFPTIFQEEPIHDLVNLHCILFAIQYASAMCWIHSGLQVKRMLGHSFGQLTALCVAGGLTLIDAIRLV
SERARLIETSWAGDHGVMLSVDASEAEVRALVNRAGDTVDLACYNGARSYVLAGDEISIQVVEKLADGMRIKRLPNTHAFHSR
LVDSIVPGLRKLAQSLKYHPTTIPVEACSEDGSAWTCVTPDQIVAHSRMPVHFDSAVQRAANHVQGPVVWLEAGSASPIVSMV
RRVVEESSSSRAHLYQASDLKSPQAQANLAKATSGLWANGIPTSHWTEYDPLAFLPASAPIAEASSEPMGLVQVLEKRPSECL
FSVNTKDPLYRTCTQGHAVVEQNLCPASLYLEMVVSAAGCLSSAGLITAMPHLQELSISAPLVLEPDGDVLLRLSQSPAEKTA
WTFSLFTQAGQKAPVSHATGRISLHPFDSTSTILSRFRSLDRLMNPSRPDSIASLPSSSGLKGSAVYQAFRRVVNYADYYRGV
ESVFCVSTEATGRVFVPLSLSRESACDPILIDNFVQVAGVHVNCLADVPEDEVYVCSAVGEAFIGEVFMKRDPAAPQPWRVYS
NYDRLSKGQVACDVFVMDQKSGQLAIAILAATFTSVSIRALTRTLAKLNNHQPSMLATNEPSAGHKEVNSILNVVDRPPPTAA

TVDTNKFPAIQAMLSDLLGVGLDELSPYSSLMAIGVDSLMSTEVLTEIKKRFGVNITSAELGEIPDIQCLVQAIFPGASVAQK
QATTSKMPPLSDLAESVFNGPAPPDALMLAQKAYDLFGTTQANTDYSQITKWAGFCESVFPKQMALVTAYVVEAFRALGYPLE
LLHAGQAVPLIPVLPQHESVRNQLYEVLKFSKLICRKDDGMFRTAEAVPSDTSLLLHEDIIKEYPHHASEHTLLRTTGSRLAE
CLSGSADPLALLFQNADARRVMEDVYTNAPMFKSATMHLAQYLQDLVILLRSRRDIKILEIGAGTGGTTKYLVSQLAAVPGLR
FEYTFTDISTSLVTLAKKKFNGYSCIQYATLNIEQDPPDDLLGQYDIVLSTNCIHATRNIAHSCDNIRKLLRPDGILCLIELT
RNLFWFDLVFGLLEGWWLFNDGRNHALATEQFWNESLRQAGYNWVNWSCNDSRESEILRLIVASPTLPHGASQILFRSPLVTE
ETVKYDEKDGVQLLADIYYPSEVDDAHRRRPIALLIHGGGHVMLSRKDIRSQQIKMLLNSGFLPVSIDYRLCPETSLTEGPMR
DVRDALVWTRRTLPRLSLKRPDIRPNGDQVVAVGWSTGGHLAMTLSWTASLCGVRAPEAILSFYCPTDYSDPFWSQPNFPYGR
DIAPQMKCTIFGMQ
>AndM
MEASQGIAVLFGPQSADIEEAVSCIQAFVRENPSATYLIDIIQSLPSIWPAIQDSWVPLAQIDGKRQLETLGSLFGGGAATVP
PKSSNILITPLTVLQHIVELCRLRKRGSSLQIKDVQGFCVGFLAATAVASAHDETQFRSIVAKVIRLAVCVGALVDLNELERG
RAASLAVRWSGEEGYSCMEKVLAAHPEAYISCLTDTNRATITVPQELQRDIIREMANQGLSVRSIPLCGRFHHPDHAPAVRQL
MQLCRSDDRFQLPDSKTLQFPLRSNIDAELVPEGPLHDIALQSILCLQSKWHATVSAALESLTERNGNVPIAAIGPEQCLPRT
ARSRLRQTWGSTLVPMGTGHTVNGNLTPSRATSPADSSSELIPTVQPIAITGMSCRYPQADSVEALWELLELGKCAVGPLPNK
RFRMDELLREPNGPFWGNYLEEPDVFDHRFFGISAREAATMDPQQRLLLQVAYEAMESAGYCGIRSSQVPEDVGCYIGVGSDD
YTDNVGSTHANAYSAPGTLQAFNTGRISHHFGWSGPSVVVDTACSSAAVAIHMACQGLYTGDCSIAVAGGVNVMTSPKVTQNL
AAASFLSPTGASKAFDASADGYCRGEGAGLVVLRPLDQALQNGDPVLGIITGTAVNQGSNCSPITVPVSRSQMSLYRKALSAS
GVSPDDVTYVEAHGTGTQVGDPIEMDSIRNTFGGTHRTEQLYVGSIKDNVGHTETSSGVAGLIKTILMLQKQTIPKQANFSRL
NPKIPALDMDNVVIPTRSTDWKVSTRVAMVTNYGAAGSNASIIVRQPFTYPGGTRTTLSHAPIFIAAKTAESLRTYCDNLLAS
LRQIPVLPDHLVSDYAYNLATKQNRTMDCYIGFATDSPASLTNQLAAVASGAREVYSRSGKALPVVLCFGGQNGNIVHISQDL
YTNTHLLQFHLADCEKACQSLGLPSLFPTIFRADPIEDLVSLHSALFATQYATAKCWIDSGLEVDRMIGHSFGQLTALCVAGG
LSLVDALRFISERARLLEKHCSGKRGLMLSVEASEEDVRGLVNGAGQAVDVTCYNGPRSFVIGGEKVAIEAFEKVATGFKTQR
LANTHAFHSHLMDPILPALREVAQSMTFQPTTIPVEACSQDMEWTFVHAAQLVTHTRQPVYFESAVRRIANKYPTGAVWLEAG
SASPVIPMIRRVVESSPNNHVYQPIDIRPPQAIANLSKATSGLWSNGTHVQFWPFHKSQSNCYNSINLPPYQFTKTRHWIDYD
PLAFMPSSAPKDAGPEEAKKLVQLLKQQSGECLFTINTKDLLYQTCTKGHAVVNQNLCPASLYLEMVISAASHVCPVELSSTM
PHVQNLCILAPLVLNPQGELQLRLSQQNGERDQWTFSLFTQQGQTSTVHATGTIWLHAITNSSAIVSRFRSLNRLMIPSRPDS
IENSPRSSGLKGAAIYQSFRRVVNYAEYYRGVENVFALTNEATGQVKIDTALVKDSCCDPILIDNFIQVAGIHVNCLSEMSED
EVYVCSAVGEIFIGEAFMQRDCGASLSWRVYSNYDRLSRNQVACDVFVMNQESGQLAIAIMGATFTGVSIRALTRTLARLNNQ
QSSAPEVHDAPIPQDTVRADAMPAATPAPLPSTLQEVDNLPAVQGMLGDLLGVGLEELAPSCSLLEIGVDSLMSTEVLTEIKK
RFNVNITSAALSEIPDIGGLVQVIFPGTSVSQTTVSQTAPNDSNAVVVESTPVGELSMLVQEAYDAFDAIRSMTDYSRETKWT
GFYDIVFPKQMALVTAYVVEAFQALGHPLQSISAGGEVPLIPVLPQHEKVRNQFYAVLEFSRLVSRTTDGRIVRTKEQIPTEA
ASNLHDEIIRQFPHHASEHMLLRTTGSQLAECLSGDANPLALLFQDAEARRLMGDVYTNAPMFKSATMHLAGYLQGVLERVGS
GRTVKILEIGAGTGGTTRYLVSQLAARGLRFEYTFTDISSSLVMLAKKGFKEYDFMRYMTLNIEQDPPQELLGQYDIIISTNC
IHATRNIAYSCSNIRRMLRPDGILCLIELTRNLFWFDLVFGLLEGWWLFDDGRKHALASEHLWNETLDKAGYSWIDWSRNDLK
ESEFLRLIVASPSKPEMPLVTQETVKFDEKDGVSLLADIYYPSVADDANQRRPIALLLHGGGHIMLSRKDIRADQTMLLLDAG
FLPVSIDYRLCPETSLIEGPMRDARDALRWARQTLPTLQLQREDIHPNGDQVVAIGWSTGGHLAMSLAWTAAELAVAAPEAVL
SFYCPTDYIDPFWSQPNFPFGKQIAPPGEIDIRAGMSPNPITAYNPPRSKRALGGWMSTTDPRSRIALHMNWKGQTLPVLLNA
AKTAPGEMLLDPTKEDIVRVCPTSQASAGNYRSPTFIIHGSLDDLIPMSQVRHTAKVMKANGVDVHLRELEGSIHLFDIASTY
GDKPDEVQAVADGIQFLKDRVQC
>Pro_AdrD
MAEPPKTPGRPVCVVFGPQSSEIDETLFYISRNIDENPSLGFLKDVLQELPSLWSPITDAWSSLSSIPGATQLTVLAECVQGT
TAAPKSAMNVFMTPLTVIRQIIDVWKFKEESQNRCRIVDAQGFCVGFLAAVAVASSNDSNEFEDIASTMIRLAVCIGAAVDLD
GILHGPARSVALRWKSDSEKEQLDRVLGSSSTAYISCFTDATSVTVTVAEDEVDDLTKELGGHGLSVKIIDLKGRFHHSRHVT
AVQYLADLCETDARFRLARTSPCVLPLRSNVDGHVIGKRFAIHKTALESILTKPSQWAITVSAAFEQARETDDDLAFVVIGTG
QFVPRLVRTRVLDHLNNKWSDTKQHAILPNGIHRSSSTTRSRPSIDMAPIGPTVIPIAITGMGCRYAQADSPEQLWEMLELGR
CGVNALPNERFKMENLLREPKGPFWGNYLANPDVFDHRFFGISAREAEAMDPQQRLLLQVGYEAMESAGYCGLRTSNIPTDVG
CYVGVGSDDYTDNVGSSNANAFSATGTLQAFCTGRLSHYFGWTGPSVVVDTACSSAAVSIHLACKALQTNECSIAVAGGVNVM
TSPRVTQNLAAASFLSPTGASKAFDASADGYCRGEGAGLVVLRPLKDAIHNGDPILAIIGGSAVNQGSNRSPITVPDSDSQIS
LYRKALVTSGVRPEDVTYVEAHGTGTQVGDPIEFESIRKTFGRPARTERLYVGSVKDNIGHTETSSGVAGLLKTVLMMQKHQI
PKQANFVQLNPKIPTLDDAAIAIPTKSIHWPSAANSSSTAVAMVTNYGAAGSNAALVVKQYKAKSGLSNPVSPLPSEVPVILA
ANTVESLRSYCKALLSSVCDAQLTSCQDTAYNLAIKQSRDMDYVSAFSIPVDRPNELIAKLESISRETTNLEKQPAARLPVVL
CFGGQNGNEATISEDLFNQCELLQYHLTECEKVCQTLDLPSLFPSIFQPGPIEDTVSLHCILFSIQYASAKSWIDSGLQVDRI
IGHSFGQLTGLCVGGGLSLSDALYLVSERAKMIHSMWGSERGAMLLVEGSEVEVQGLLNRAAEHMADVAVDVACVNGPRNTIL
AGDERSLQMIEKLSAKAPSILRTKRLKNTHAFHSRLVDNIVPPLTKVAQQLQYKPLSIPIEACSQYDDWTYVTPGKIVDHSRR
RVDFQTAVERVAQRIQGPAIWLEAGSASPIIPLVRRVIDTVAAFSNGHVYQALDLGGALAHRSLSQATCNLWSRGVKVQFWQF
HDSQAKSYNWINLPPYQFAQTRHWIGYDPNAFASLPEVKPTVPSSDAPKEFVQLLTKQPTECVFAINTNDPLYQECTQGHAVL
DQNLCPASLYFEIIVRAAGLIRPENDISPAMPHLKDLAISAPLVLNPRGNVMLSLTRARVGDSTWSFSLFTRESNKNKVTTHA
TGEISLHPFGQNTPLFVRLHSMNRLIDSSRVDSIANSRESSGLKGFAVYQAFRRVVNYADYYRGVEQVFATDHEAAGIVNLPS
SRTKDASCDPMLVDNFIQVAGIHVNCLSETKEDEVFVCTGVGEILIGEAFMTRDPNCSRSWAVYSNVDRSIKNKITCDTFVLD
RETDKLAVTILSATFTSVSIAGLSRVLKKLNNQPDDKKVPLGQSLRDDSKVALNPTPQNALAAVPAPLHSAPDSGHFMVVQEM
LCDLLGIASDELLPSSNLEDIGVDSLMRTEVLVEIKKRFNFTIDTSSFVEIPDILTLVQTIFPDAATAPLTNGVHPSLQIETT
EAVDSESNTHVIPTPISDEEIHGLIDIAPGLFTDIQRSMVHSQSTQWDGFCESVYPRQMALVTAYVVEAFKSLGVSLESFEAE
HLIPQVPVLQQHSKVRSQLYSILQFSNLIRATDHGFVRTSVPVSTISSDVLHEEIIRLYPQHTSEHNLLRTTGSRLSDCLSGA
ADPLSLLFQDAEARRLMEDVYTNAPMFKAATNHLAQYLVNLLGRVDTTREIKILEIGGGTGGTTKALLSQLTAVPGLRFQYTF
TDLSSGLLALARKKFKHYSFMKYQVLNIERAPTPDMLGQYDIVLSSNCVHATRSLVQSCSNINKLLRPDGLLCLIELTRNLFW
FDLVFGLLEGWWLFEDGRQHALATEHVWKQTLSQSGFQWVDWTYNDSQESNVLRVITASPTSAVILPPSPRSPLYLMNEETIV
YGKNDGVELSADIYYPRDLQPIGKPRPIALLIHGGGHIMLSRRDIRSKQVRMLLNAGFLPVSVDYRLCPEVSLTEGPMHDVCD
ALCWARHVLPSLTLGRPDIQPDGTQAVAVGWSTGAHLAMTLAWTSQQRGIAPPNAILAFYGPTDYEDSFWSQPNFPYGKNAAS

PEMRYDLWEGIYETPITAYNPPVDQKALGGWMSPADPRSRIALHMNWKGQSLPMLLHGGRFWSDHKDGDCGEELPVPTLEEIQ
AVSPLAQIRNGHYKTPTFIIHGTLDDLIPVEQAQRTSQELVTKGVEVQLRVVDKAVHLFDIYPGFEKDQAASRAVEDGYEFLR
DHVRY
>Pru_AdrD
MVDLSQTSPGRPVCLVFGPQIAEIDESLFYISRNIDENPALHFLKDVLRELPSLWSTISDTWAPLSSIPGAAQLTALADCVQG
GPIATHENPTNVLLTPLTVIRQIIDAWKFKEKSQNKCRIMDAQGFCVGFLAAVAVACSNDAKEFADIASTMVRLAVCIGTAVD
LDGISHGQARSVAVRWKSASENEQLNRLLTSSSTAYVSCFTDTNSATVTVAEDAVDDLIKELGSHGLSVKIIDLKGRFHHASH
ITAVQYLSSLCDTDDRLRLAGTGTCVLPLRSNVDGHLIGKISDIHKIALESILTKPSQWAMTVSAAVEHSRETNDDLSLAAIG
TGQFVPRLVRNRVLDHTNNSLWDTKHEMLPNGIHKSSFPTESMQSTNMAAMAGTATPIAITGMGCRYAQADSPEQLWEMLELG
QCGVSALPNERFKMDKLRREPKGPFWGNYLANPDVFDHRFFGISAREADAMDPQQRLLLQVGYEAMESAGYCGLRNPNVPTDI
GCYVGVGSDDYTENVGSTHANAFSATGTLQAFCTGRLSHYFGWTGPSVVVDTACSSAAVSIHLACKALQTNECSIAVAGGVNV
MTSPRVTQNLAAASFLSPTGASKAFDATANGYCRGEGAGLVVLRPLADAIRNGDPILAVIGGSAVNQGSNCSPITVPDSNSQR
SLYRKALLASGIPPEDVTYVEAHGTGTQVGDPIEFDSIRKAFGGPGRSEKLHVGSIKDNIGHTETASGVAGLLKTVLMMQKQQ
IPKQANFVQLNPKIPALDDAEIAIPTKSIHWPSAATSSSNAVAMVTNYGAAGSNAALVVKQYKAPSEPSNRASLLPSEVPIIL
AANSVESLRSYCKVLLPSVRNAQLGSCQDIAYNLAVKQSRDMDYISTLTVPADQPNELIAKLESMSTETTNPKKQPSSRLPVI
LCFGGQNGNETTLSEDLFNQCELLQYHLMECEKVCRTRDLPSLFPRIFQTGPIEDTVSLHCILFSIQYASAMSWISSGLQVDR
IIGHSFGQLTGLCVAGGLNLSDALYLVSERARMIQSMWGSERGAMLLVEGTEADVQSLLNRATQQMADAAVDVACVNGPRNII
LAGDERSLQMIQKLSAETPSILRTKRLKNTHAFHSRLVDSIVPSLSKVAQQLQYTPLSIPLEACWQDGDWSFVSPDKIVAHSR
GRVDFQTAVERVAQRIQGPAIWLEAGSASPIIPLVRRVIDTVTASSKDHLYQSLDLGGPQGQKNLSQATCNLWSRGAKVQFWQ
FHGSQAKSYNWINLPPYQFAQTRHWIAYDPNAFAPLPEDKPTVPSSGGPKEFVQLLTKQPTECVFAINTKDHLYQECTQGHAV
LDQNLCPASLYFEVIVRAAGLVRPENDTSPSMPHLQNLAISAPLVLNPTGNVLLSLTRARAGDSPWSFSLYTREPNTNLVTTH
ATGEISLHPFGQNTPLFVRLHSMNRLIDSSRVDSIANSRESSGLKGFAVYQAFRRVVNYADCYRGVERVFATEHEAAGIVNLL
SSKTKDAACDPMLVDNFIQVAGIHVNCLSETNEDEVFVCTGVGEILIGEAFMTRDPKSSRSWGVYSNMDRSVKNKIACDTFVL
DRETGKLAVTILSAEFTSVSIAGLARVLKKLNNQADDEKASPDLSLRNDSKVDVNPTPQNTAPVVQPTRQAAAEPGYFVVVQE
MLCDLLGIVSEELLPSSNLEEIGVDSLMRTEVLVEIKKRFNVSIDASTLTEIPNIQALVQTIFPDAATAPLTHGVHPSLEIET
TDVPDSENNTHVIPTPISDADVHGLIDIAPTLFTDIQRSTSHSEMTQWNGFCESVYPKQMALVTAYVVEAFKSLGVSLDKFEA
EGVIPQVPVLKQHGKVRNQLYSILEFSNLIRATDRGFVRTTIPVPTISSDVLHEEIIRLYPQHRSEHHLLKTTGSRLSDCLSG
AADPLSLLFQDAEARRLMEDVYTNAPMFKGATNHLAQYLVNLLGRMDTTREINILEIGGGTGGTTKALLNQLTAVPGLRFQYT
FTDLSSGLLTLARKKFKHYNFMKYQVLNVEQTPTPDMVGQYDIILSSNCVHATRNLVQSCSNINKLLRPDGILCLIELTRNLF
WFDLVFGLLEGWWLFEDGRQHALATEHMWKQTLVQSGFQWVDWTHNDSEESNVLRVITASPTSAVILPPTPGSPLRVMNEETV
PYGKNGAVELSADIYYPRDLQPIGKPRPIALLIHGGGHIMLSRRDVRSKQVKMLLDAGFLPVSVDYRLCPEVSLSEGPMHDVC
DALSWARNVLPKLSLCRPDIQSDGTQVVAVGWSTGAHLAMTLAWTAEQRGIEPPQAILAFYGPTDYEDPFWSKPNFPYGKSAA
SPEMSYNLWEGMHETPITAYNPPANQNALGGWMSPADPRSRIALHMNWKGQSLPMLLHGGHFWSAHKDGDCGEDLPVPTLKEI
QAVSPLAQIRNGCYKTPTFIIHGTLDDLIPVEQAQRTSQELVTKGVEVELRIVDKAVHLFDIYPGFEKDHAAAQAVQDGYEFL
RDHVRY
>InsA2
MGSQSPCSEHQPPVSVFFGPVYPELTESSSHIRQYLSDEGSAGWLDDTLQGLPSVWEDIVRQWPALRKTSGEPQLRQLTQYLR
RESSSPVRENLNLLLVPVTVLRHIVEFRKLKDERKNLEIKDVQGFCVGVLAAITVCWEHDDVDFAKVVSTVLRVAVCIGALVD
LDELHGAPSKSMAVRWKTKCENRQLGEVLERYKGYIACMIKTNGATVTVPSENYRSVTEDLESYGISVKSIPLRGRFHTPDHI
PAMEQLLALCAGDARYQLPIKKNPHLLPRSNVDGTRIPSNSLVAVAVESILAKQANWMLTVAEALNSDGPADEKHAVIIGAGQ
IIPQRSFLASVEHIGNQMAPNDTSPPLPNAALDLRSTAQCNGAFPKPTTRALSTPIAVTGFACRYPQADSVEALWTLLERGQC
TVSPMPNHRLKADSLQRQPAGPFWGNFLQSPESFDHRFFGVSAREAESMDPQQRLLLQVAYEAIESATYCGLRNTELPDDVGC
YVGVGTDDYSENVGSHDATAFSATGTLQAFNSGRISHFFGWTGPSITVDTACSSAAVAIHLACQALHTNDCSVAVAGGVNVMT
DPRWSQNLAAASFLSPTGASRAFDAAADGYCRGEGAGLLVLRPLDAALRDGDPIHAVITGTCVNQGANCSPITVPDSNSQRSL
YMKALAQSGLHPDAVSYVEAHGTGTQVGDPIEYESIRSTFGGPQRTEKLHIGSIKDNIGHTETSSGAAGMLKTILMIQKRRIP
KQANFSRLNPRIVTHERDQIAIPTQSLDWKAAERVALVTNYGAAGSNAAIVLKQPGRASNEPAVDRSRWPARVPFIITAKTEE
SLREYCRELQHILRAEQQESPAATHHLAYNLAAKQNRGLEYLVSFSCEQAEVSARLQDIADCRSKPVRCIQPPPTIVLCFGGQ
TGDMAGISPSLVENCDILRSHLTDCDETCHTLGLPGLFPTIFSPEPRRDLVSLHCILFSIQYASAKAWLDCGLVVDRMIGHSF
GQLTAICVAGGLSLIDGLRLISQRAALIQEKWGSERGVMLSLKASEIQIQELLRAASDTVDVACFNGPQSFVLAGDEKSIAHV
ETLCVQRGLQHQKKRLRNTHAFHSRLVDPLLPGLSQVAETLDYRPLRIPVEACSEEPDHWARITPFKIVRHSRDPVYFHPAVQ
RVRRHIPGSCVWLEAGSGSPIVGMVRRVVEAAGPAGEHTYLPMELQDSTAEGNLADVAKVLWSKGVPVQFWPFHRSQVGHKWI
NLPPYQFSKTQHWIDYDPYAFHPTGAVAEEKKNNDGLLRLVKQEANGCLFRINNQDAAYRMCTEGHAVVDQNLCPASLYVEIV
VRGAMTLSTSGQPATMAHIEALNISAPLVVDMPGSVSLRLTRTAKDNDGGWMFSLYSQDGDCPSITHATGKVLLVPQSTGSPA
SARFHSLNRLLDPGQFDSMAKSPSSNGLKRATVYQAFRRAVNYADYYRGVVEVYAVGSKAAGRVLLPASPTRMAACDPILIDN
FLQVAGIHVNCLSETDADEVFVCSSVGEVSLGDRFLNRDTATPKAWTVYSTYERESEKKVTCDVFALDEDRTLAMTIMSATFT
SVSIQSLKRTLSRLNGQTPALSSSSSVGQQPPQPQPKVHEQIAPPAHITISDNDNLRDVQAMLGELLGVSPGELPSKASLVEI
GVDSLMSTEVLAEVDKRFGVKITNSELTDIADVRALAYRIFPSSSSVVHVETFKESTVAIDISIGGQKPIVDSSPIVHQEDSL
QFADSALTAFASTRGSTKHTDQTQFAGFCTSVYPRQMQLVTAYVVEAFQALGAKLESMLPGQAVPSLAILPQHTQVLGQLISV
LEHAGLVERKGTDVFRTTKPVDVGPSAVLHQTILADYPQHASEHKLLHTTGARLAECLTGTADPLSLLFQDAQARALMQDVYS
NAPMFKAATMQLAQYLQNLLLGSGCDRDIEILEIGAGTGGTTAFLVSQLAAIPGVKFTYTFTDLSSSLVTLARKRFGSYSFMR
YSTLDIEKIPGEELLGKYDIILSSNCIHATRSLATSCTHIRKMLRPHGILCLIELTRNLPWFDLVFGLLEGWWLFNDGRSHAL
ANESLWQARLREAGFNWVDWTDNALEESDILRLIVASATRPSTALPLGPSVAPARVETVKYAERDGLQLMADIYYPHSIDPKG
TKRPIALLIHGGGHIMLSRKDIRPAQVDLLLDVGFLPVSIDYRLCPEVSLLEGPMPDVRDALAWARTDLPHRPLSRSDVQSDG
DHVVAVGWSTGGHLAMTLAWTAPEHGIRPPQAILAFYGPTDYTDPFWTTPNFPYAGAVSEEHTKLTRPLDALHDSPITAYNPP
PNKQALGGWMAPSDPRSQIALHMNWTGQALSVLFNGCNYKKLAAAKGHSAGEVTLPAPPLADIQRACPLSQIVAGRYRTPTFL
IHGSLDDLIPVEQAQRTQDALRAAGVESTLRVVEGGLHLFDLGIELETNGTTGSMVDEEGWRAVREGYDFLRQHVAV
>DtbA
MEQPALVIFGPQISWPSNSSAERIRRDLVQEPSLAPFADAIRKIPELWASLVEIEPRLRETQGRRNLEQLRDWVEIGSLPRRD
SPDEILPNGFATPFTVIAQSIEYWRCFPRGGLQWLHSRSEAGPALEGMCTGFLTAIAAATSKDSTEFFTMAAIAVRLAACIGT

FVDLNGNTSDGTGEARALVVRWSSPIQESCLLRILEEQPNAYVSVLKDETSRTITVPASEIPSTAEKLLMNGLRVHALELKGR
FHHASNEDIANKLLQMCQLNADLHFPDAQGLLTTLRRNSDGRRLSKGPLHIIAIRSLLLDQVDWLSTMTSALESAVIKDKPPK
ILVFGSVNVVPHCLIQRSGARVCVSNDMIQPLSNITTTVKDFVPEPDMTKPGVKSLPDAPGCDNHDNSIAIVGMACRFPGADS
AEEFWELLECGKSMLSVLPESRFPTKGLRRSADDTVFWGNFIQDPDQFDNRFFQISSREAASMDPQQRLILQVAYQALESSGY
FDGQRKPTHTNIGCYLGVGSVDYEANIYSHQPNSYSALGSLKAFVSGRVSHYFGWTGPSITYDTACSSSVVALHSACKALLSG
ECTSALSGGVNVITNPALYQNLRAASFLSPTGATKAFDAAADGYSRGEGCGLFVLKRLENALADGDQVLGVIAASAVNQTQRE
YGTSITAPVSKSQQSLYRHVLSQAGLEPSDVTYIEAHGTGTPRGDPIECESIRTVFGSTNTGRQQTLYFGSVKGNIGHLEAGS
GAAALFKSVLMLQKKKIPKHMNFNKLNPQIPPLEPEKLAIPTKTISWDAPFRAICISNYGAAGSNGALVLREAPEPLKSTCAR
PTGNGPLRCQLISIAADSAESLQAYCNQLLRYLADVNERHFPDVAYKLARIQNPAHKYRLSFVTRSISELRSFLSAQSHVTPT
PSFSGTVKPVVLCFGGQSKSSVGINKQLYDSISLFRHHLDRVEAACQALGKTIYPFIFSKEVIQDSVTLHCCLFASQYACARS
WIDAGIKVATVIGHSFGQISALCVCGVLSLEDAVKYVIERALLFDINWSAEKGTMLALKGDTQIVTRLVSTSQVEIACYNGPR
DVVVAGSIQAIEGFQSRAKAAGIDVRCLDVARAFHSSLMDPALPQLRRIANALTFYRPYIPIQTCTQGHGRDSFDPEFLVDHS
RQPVFFHDAVQRIESQLGPCTWIEAGSSSFGTTLASRIASQLNSFIPVDIGGPSPAESLANATLKLQALGYNVQFWAYHRSQR
DAFRVFNVPPYQFAKSKHWLEFKEFNSLAKESSHTDDNDQHSKLLLFVQSRDREKGIAEFKVNANSDYFRACVSGHAVLGHSL
CPASLYLELAAQAAVYICENESPVLPQVEDLQISAPLGLNPSATIKLLLSLKEPKVWRFSLLSTTGAEDIHHASGTIQVPANA
DFLVSESSLYERLIGADRCNSILQDASIKSINGLVYNVFGAVVDYKDFYQGVQRISATKSEASGVVSLPVAAAGIMQDSVYDP
LALDNFLQVSGIHINCFRDFGRQEVYVCTSIGKLKVYQSLEKVRDQSWLVYSNTKQGGPGVLESDIFAFNKTTGSLMVALFGV
RFAKVSVNSLTKTLSALNHGKPVQRLKDSTPPSTPLSNLGLGTSPKTELDGNVLLRLQRLLSRVTDCPVEDILQVNSLEALGI
DSLMRSEVTAEIRQEFGLDISTDTLAKASTLFHLTTLISSSQKWPVQIVNTPSSSESHTTLSMRDSTSSTLKDILSAITDIPV
DEIGNDSTLEALGIDSLMRKEVQSELRKNLGRADIPENLHEYSVTSLAAYLADQDCGKRNDLSCGTPPGSVSLGRHCDIKALG
SHTQLMAPEMISGLEERQELVKRNDRSQLFQLSSPPKPLCSSTVGGFTLKDLAASFEEIKGAFDQLSDESQFLNFYAQVHPRQ
MKLATAYVVEAFRSLGCPLSSMSPGESLSLSPVSYEPRHGNLMKQLYRLLQDAGLISLDNLGEYKRTSKPVDCTSSADLLDAI
IQDFPQHQLEHRLLSITGSRLASCLSGELDALDLIFGDRTAKDLVSDVYLKAPVFVTGTKLLCKFLLSCLRQRTQPIRILEIG
AGTGGTTADVVRCLISSNIPFEYCFTDLSPSLVAAARKKFKWCPNMEFNVLDIEQEPPSVNQYDLVVSTNCIHATRNLTATTK
NIHKLLKEGGILCLVELTRNLPWFDLVFGLLEGWWLFNDGRQHALAHELFWKDSLRQAGFSYINWTTGDTKESEQLRLIISVK
MDHQYDLSKWETGSEKCTGDNFEIVTSRQPLSVYSTPDLLKEPGLSGSVVLLTGGTGNLGTHVLHQLINRADVRRVICLNRLT
TNDDPIQRQRRALRDKGIDLKERQWEKIEVLEAKSSHTALGLQTEQYQRLRDQVTHIVHNAWPMSFKRSLHTFEPQFKTLQNL
LKLCHEAKYGARLLFISSIGVVGRHPNTFANKPVPEDPVRDCQSSLGFGYSQAKYHCEQIINRALEQDTRLEASYVRIGQITG
SQHFGLWNTEEHVPALLRTSQTIGALPHLDGLASWLPIDIAAATVSELLLDTAHLRMVYHVENPVRQPWCELLGYLSAHLRLP
IIPYKEWLSRMETNGDSVTGSPNPAKNLRDFFKNDFLHMSCGSVVMSTTSTASVSAALRSAGPVSPGTLLLYIEQWRRTGFLG
>DrcA
MASPWSSNPLPSLAIFGPQSKTPSPRYLSELRSYIRSKAVLVPLVQAIKNLPRVWAAFRPLHPGFGTLEDGPACLQTLSDWIS
SDEEGIGALDGVPSGMLALPLLLVIHLAQYFQFLDQTNRTHHEMINHFRSGGGIQGYCGGMLAAVSVACAHDEEEIVAHACKM
IRVAVGIGAAGDVGDDNPSKMSTIMVVRLKREGQAEEIVKSFSGTHISAITDARTVSIVGPARCLLELQQWAVAQKLTVQLIP
LRGKLHHPDNLQLVEALSKFCDADEQLRIPHAPSALVPFRSNRTTKISTEGSFSLEIARTILVDKCEWYQLLSEVAMDLSHTG
RTTHSLAAFGIGDCVSLKPFRDAGIQMSKMDMFTLLGTTPTRAPELEPSPGIPTDQSSHAIAVVGVACRFPGANSVEELWEVI
SSGKSMVQEVPTERINFQDSFRAQSDSKWASKQKFYGNFIDDHDCFDYSFFRVSSKEATYMDPQQCLLLETAYQAMESSGYIC
STQSRDAGDRVGVFLGASFIDYLEHTSAHAPNAYTATGTITAFLSGKISHYFGWTGPSEVIDTACSSSLVAISRACRALQRGE
CTTALAGGVNFLGTATHFLDLGRAGFLSPTGQCKPFDAAADGYCRSEGVGLLVLKPLAEAMANHDNILAVIPGIATNQGSDPK
SITVPHTASQLQLYKDILQEANMNPSLVTYVEAHGTGTQVGDPVEIEGIRRGFAGPTRDHALYVGSIKANIGHCESAAGVAGV
IKAILMINKGLIPPLANFQRLNPKISSIEPAKMVIPTATQPWKAPFRAICVNSYGAAGSNAALLLCQSPQQDKLDAALHPRLP
STYPIILTAASRNSLSANARSLKKYLAASTPNIGDVALTLFQCRKPQRFAWTCTAEDISTLSGSLDALVNVTEVPQIPKKLVL
AFSGQAKQAVGLHKGLYESCPLLQQHLDRCDQYLVRTGSPSLYPWIFETKPRFDVVLLQCSLFALQYACAMAWIESGLKVDAV
VGHSFGELTAMAVAGVMSLEDAMDLIRTRAQLIQSKWGPEKGTMLAIHGTEAVVRDIIARLPDGSGEVEIACYNSPTSHVIVG
SADAVAAIENVLLQEPSFRGIASSKVDTTHGFHSRFSESLLGDLDQAAERCHFSSPQIPVESSTRDPVSHISFQRIAQHMRRP
VFFYHAVHRLEQRLGSCIWLEAGVDSPIIPMIKRAVSAPQEHVFLPIKLGTRQNPMDAVSEATAQLWREGVPVSFFNFKTAGD
KPIWLPPYSFERTKAWLAYVDPVQEALRTRNLTAPEPRTESKEVSLKLVTSYTDAQQDPNTLCFGVGTQTERFRSIVAGHNVI
GHPLCPAALYLEFVIMAATSVLDSGDRPGFSFWDFRIDAPLGIDTRRAVHLTLRGKHSADWTFTVESSAVGNLKSKSTVHAKA
SFRLFAALDGHQERQFQYQQRVVQARLSNFLASAPPTETFRTHRAYKLFSRVVNYSSILQGIDVIVMAGTEVMAELALPAASD
TEESTAVGACDTIALDNYVQVLGLLINTSDLCADDEAYLATGADTIVVHPGCDFKAMRKGRVYASFCAVGDAKATGDIFVLDN
DEALVVTITGMHFSKLPLSTLHKILGPVTQEDTSSRSRLNQQVSSSPAIHLPTPSSTAAKVLNEGPSTSSTQNGIDEAGIVAL
KQVMAEFCGLSPQEISINTAIADLGVDSLAAIELVDELRSRFHVEIDSSEILATNIEGIVRLLPKMQSRTPDLDKALSGTNPS
RSASTFHSPASNQTSINSGLQEHMLTLIAEYAAIDVHSITPDVTLEALGVDSLSLIELRSSVQEAFGVELDFDLFTTVTALVS
LLNQTSDHHMLQPMRPPLADAIQVPSNPNWGTSALKDAVFKVLAEYAAVDMSSIVETASLDAIGFDSLSLIELKSALEIYGGE
LELDLSTTVEDLLSTVGAASNAGYEPLTPSSSPPTSSATSLETPATPFSQEDEGLGNTVLDNNSSPSRDPLEALNDCQGLFTA
AADRHGLTGYWETVVPQQDELTIAYITEAFEQLGVRLRDLQEGETVPELYCSPKHSRLVSRLWDILTEYGIVKRRGSQITRWT
RSLPSLLPEALLDRLITSHPKYTTDAQLISVTAPHLARCLTEQEDAVRLLFGTPTGKDVLNKFYSDSPLFKPSADLLLDLVDR
VIPRGSPEAPFRILEVGAGTGGTTKQLCELLQSRNKAVQYTFTDISPTLVNAAKRKFSQQYPWMDFCTLDLEKEPPATLQAKY
DLVLGTNVVHATSHITRSCTRIRSMLRKDGFVVLLELTRNINWFDLVFGLLSGWWSAKDGRNYPLQAPHAWVGYLQEAGFGSC
GLTTGSTKESTTQQIIIGSTRLQKIGTKYETQRYELRTVPYKVVDDLPIEADIYYPPPQDRSRNKPMPIALMLHGGGYMTLSR
RAIRPHQTQYLLTNNILPISLDYRLCPEVDVLSGPITDICDALVWARTTLPGIAKEQGVLVDVDKLVVVGWSTGGHLAMTTGW
TCFAKGVKPPTAILSFYAPTDFEHEYWTRRDLGAFPAPTMNVESIERALSGKVISHYDGPKGIGASEENLGLLRQGDPRSELI
LNLFSGGNGLSLLLNGFTGSLNRLQPPTTIQIQSISPLAHVRDGSYSTPTFIIHGEKDEVVPCQMSVSLIEEMKARGLDGEVL
VVPHRRHLFDLTLKPGDAQWEKWIEPGYQFLFRHLLVEGTGANST*
>Atr1
MASNHEELPSMVVFSPQSKAPKEGYLDELRSYLCGKAELRPLLDGIENLPNTWSIFAQRNSDIAALTQGIRYTQALSDWAKHG
TSSGISNVMSGILSLPLLTIIQVVQYFQFLEVKKLRHSDFMERLRCRGGVQGYCGGLMPAIAIACSATEAEVVTNAVKAMGIA
LGVGAYGELGDDENVLGPTTIVVRLKQEGQGGDIIKDFPDAHISAITDPKTVSIVGSAPSLAEIQARVKSNGMQTQAMHLRGK
VHNPENANLALELCMLCDEHEELSLPNASHLTAPLRSNKTGKKLLDVSLTHEAIETILASCCQWYDLLKGVCKDLEKTGTQSH

LFASFGIGDCIPLTPFHQAGLQITKLDVLSFVKALMPPVLPMSGHNHQYAYPTDAVAVVGMACRLPGANSVEELWDLISSGGS
TVTPVPEDRMDIAGSFRAMQDPKWAAKQQWWGNFISDIAGFDHSFFRMSPREAASMDPQQRILLETAYQAMESSGYLGSHRRE
SGDPVGVFLGASFVEYLDNTSSNPPTAYTSTGTIRAFLSGKISYYFGWTGPSEILDTACSSSLVAINRACKAIQNDECPMALA
GGVNLITGIHNYLDLAKAGFLSPSGQCKPFDGAADGYCRSEGAGLVVLKRLSQALTDGNQILGVITGASTNQGGLSPSLTVPH
SAAQVKLYQNILHQAGMRPEQVSYCETHGTGTQAGDPLEIESVREVFGGPKRQDSMHIGSIKGNIGHCETAAGVAGLLKALVM
VNKAAIPPLASHKSLNPKIAALEPDKLAISSCLEDWRVSPRAALVNSYGAAGSNSAVLLCQAPDIDNAPLHRVATTEHTYPII
LSAASKPSLLSNAENIASYLRKATSKCTIADVAFTLVKQRKRHPLQWITMESSIDGLVKSLGSLQDPSNAPQPKKVVMTFSGQ
SRQSIGLNKEWYDSFPLFRRHVNECDDLLQQSGFPSCKSAMFDKEPARDVVPLQCAMFAVQYASALSWIDCGLQVEAVIGHSF
GELTALAVSGTLSLKDALNLVATRATLMQSKWGPHKGTMLLISAATEMVRKIIAGNRDVEIACHNAPTSQIVVGTQAAISEVE
KVLENNTEYRGIQSQRLNVTHGFHSQFTEPLLENLSESARSLVFHEPKILLECCTLEELNHVGPDHLARHTREPVYFYHAVRR
LEQRLGTCLWLEAGFDSPIIPMTKRAVEFPERHTFLDMKTPSGTNPTKMLTTATINLWQNGATSSFWGFHPIETTNIKQVWLP
PYQFDRTSHWMPYTDHALEMSKIQAVISNSEPLVELSTKPPRLVEPRTKPSEKGEFSMNTQARRYTEIVSGHAVLSRPLCPAA
MYMECAIMAAQLSIGNIVGQAPWFENLTFEAPLGMDPDNDTTVVLKDDGSKSRWSFVARSTSRSNPKRKPVLHAKGDFGFTTQ
TQVHRYERLVTDRMRHLQHSKSETLKSKRAYGLFSRIVRYAELLKGISSITLGDSEASAIIDVPLGASTEDSSATGLCDCVAL
DAFIQVVGLLINSGDDCAEDEVFVATGVENFSMSLACDFDRCRTWLVFAMFTPSGNGKAMGDVFILTRDNVLVMTIMGVQFTK
LPITRLEKLLDSANPKAHNTPILKSSQQDSIVSASSSSSTEHSDDDSEDDGSRSPSHSDTSVDSESEAPADNGAAKKLKSLIA
SYVGIAEDAISDDANIADLGVDSLAATELADEISNDFAKEIDGGELPMMTFGELCRIVAPEMAAKPAKAKKKIPYKGKDEATV
VESHPGKSQSEIKDLKAVVEPLPRSTPMLSDTTVVRSDPTQVLRQIDTMFQSSADTFGFTDYWTAVAPKQNKLVLAYIGEEFR
KLGLDLWAVQPGATLPHIEYLPKHEKVVQRLWDILADHGIVYNYDAAKVRSSKPLPDAPSTALLNELNALFPNFANENRLMSV
TAPHFADGLRGKTDHISLLFGSQRGQECLNDFYNNSPQLAVMTDHLLTFFKQLLKEAPLEGSLRILEVGGGFGGTTKRLAEML
EALGQPVEYTFTDVSSMLVKEARKKFSKHSWMDFQSLNLEKDPPASLQRTYDIVIGTNVVHATSNIVNSTTRMRSLLRKGGFI
VLSEVTRIVDWYDLVYGLLDGWWAFKDSRTYPLQPADDWVRDLMKAGFETASYSRGDSEESNTQQLIIGSTRPSKVASTSGLS
EARLSKSYRIETMPYKIIDDTEILADVFFPEHEVASEAMPIALMIHGGGFMTLSKTAIRPYQTQFLVENGYLPISIDYRLCPE
IDLIAGPMTDVRDALTWVRKQLPAIARTRGINVDPTKVVVIGWSTGGHLALTTAWTCEDIGEEPPVAVLSFYGPTNFESEDID
RRRAEQYPERTMSFDRIRKSLSTKPITSYDCPTGTDSTGLGWVRPGDPRSELVLSLFKESHGLQVMLNGLSAADLAKPPPLAK
IQAISPMAQLKAGRYNVPTFVIHSDCDEIAPFRDSEAFVEELARRGVKTGLGRVRGKKHIHDLALKPEKDGWVDGAGVGYEFI
FDVVGRGVRG
>RosJ
MTISVGVFCPQSRAPSASYLQSIRQFILTHPILQCLITEVVTLKEVQTLLAMKNAAIDRLPRALQYTDYLIQWLVTGDGDAAA
STQSGIVALPRLVILQVAQYFQYLESQQITHADVIAQVRPAGGLQGYCGGLPAALTLSCAASETEVGPLICTAIRLAYAIGLY
AELGDDSTVPGATTIVVRLKSEGQAEDLVRHYRHTYISAITDPRSVSLVGPVDELAALQERASDLGLLLQAMDIRGKTHNPEN
MDLARELVGVCRESEMLQLPGPEHLKSPVRSNRTGDLITSGGSLSEEIVQTVLASRCEWYQLLCNVARGLQETGQTAHQIVSF
GVGDVVPLMPFNKLGLRIEKQDWGAPKRRLGQEKLPSYPDDAVAIVGASCRLPGANTMEELWEVLSEGQDRHEMLPRSRFDLH
GAYRARQSGPFAVQRQFYGNFLDGVDQFDHAFFGISKREMSNIDPQQRVLLELAYEAMESSGYLKSHRRDRGDSVGCFIGASF
VEYLDNTNAHAPTAYTSTGTIRAFLCGRLSYYFGWRGPAEVIDTACSSSLVAINRAVKAIQRGECSIALTGGINLITGINNFL
DLARAGFLSPTGQCKPFDERADGYCRAEGAGLVVLKPLRQALMDQDRIMAVIPGAATNQGGLSCGITVPEPKAQVELYRAVLD
QARLDPTQITYVEAHGTGTQAGDPLEVQSIRSVLGGSTRSTDLTIGSVKGNIGHCETAAGVAGVLKVICMLERRSLVPQASHT
SWNPTIPALAPDRMRLCSTLQPWTAPLLAALVNSYGAAGSNAAIVCCESPRQPAPLQQQAQIQRRWRYPVICSASTESSLRRY
QQALAAFLRRKTPPPAMAALVYTLSEKRPLHKFASIIESEGVEDLARQLADGAPTPIIERTSSPAPIVLVFGGQSRETIGLRQ
DLYQHCVAFRAYLDLCNNTLQELGYPSILPAVFDTADLSDIVVLQTGFVSVQYASALTWIDAGLDVTGLIGHSLGELTCLAVS
GMLSLRDMLRLVAGRATSMKVRWGGDRGKMSAVFASLEEVKALIEGQPLEIACYNAEKSYVVSGETRAMATLQARLLASPVRS
VAVDTSHGFHSMLVEPVLEDLSRLGESLHWSAPQIPMYPCSREARDTPLPYNPGDHAREPVFFAHSVRRIEAQLGACIWLEAG
MDTPIIQMTKRAATSRTHAFCAMATKDGVQGVNALSHTIASLWSLSVPVTHWLFLQPHDQVWLPPYQFEATRGWLDNIDRAAE
IQQHLDQHTAVAAREDARHAKEDAIPRSKLVSLLPSTEDRTEDRVRHFRIDGMSERFRKIVSGHAVRQRPLCPASVYLECVTM
ALDLIGEQEGGHPMKGTEWAFEDLSIQAPLATGAALIELTLEDSPDAAGRRYMFLVRSRSQPLSTNHTLHATGCVTREPSSNL
QPVGRLVGRHVQALQDSGDVERMQAKRAYGLFSRVVTYAEFLRGISQIRIRGSEGLATVIIPGGQPGLDESTAIARCDAVSLD
NFIQVVGLLMNTSELVGVDEVMVCTGIDNCTLSRECDLPLGDGDKHNPAQVYVSYKDCGSGKAVGDVFVFSSRGTLIAAITGC
HFTKIAISRLERLLDTANRTTAAAHQARAVAPVAVDGRLMTDSSEAEMPMTPDLSSGSGPEEDEDEDENEDEQSLPDVRAMLE
SYTGATASKILNTAVLTDLGLDSLAAVEMLGELAALCDVTLDSQDLLTITVQELETQIGHAHSARRSPPRRQSPKATAQKSCT
APARPSRHTTRGVKAIAQVKQVISELTGIEVDQVQHDMTLEQLGVDSLALVEVVAAVSDAAALPIPSEGIVLSSTIVDLLALL
PTVGQEASKPEPPSNNCPAVTALVPFTPTPIRPSLEQGPSGPRIRLDPMRGMRYSNAKLADHAARRGYTSYWDDVAIRQDQQT
LAYVVQAFATLGVDLQSLRPGEPIPHIDVLPQYRRLLQRLWAILMKHGAVAQGGQGVRIRGPKEDLIPPPDHLHAEFVAQCPT
YAIEARLLALTAPYLATVLQGSVDPVKLLFGSPAASQIMEGYYGCSPMLSTATDQMVDFVTGCVGGSQPKAAVFRILEVGAGT
GGTTVRLVEALAKCGVAVHYTFTDISASMVAKARRRFAKVKWMHFERLDLEQSSLPHMRESFDMVIGTNCVHATRNRVLAISR
IRDILAPEGLIVLSEVTRIIDWYDLVFGLLDGWWLAADEGLTYPLRAASEWVKSLHRAGFGSVLVSEGRSEDLNTQQLLVGCK
ACDMPGPNDGQSPVLRAPATTQMAMIKESVTYKIVHEVELEADVFLPAPSMADVNLKNPMAIALMVHGGGHMTLSRQSVRPAQ
TAYLLANGFVPVSIDYRLCPEVNIIDGAMADVRDAYLWARHTLPKWTMLRQRGIQLDAERIVLVGWSTGGQLAMSTAWTVAAA
GERLPSAILSFYSPCDFLSTADVFASPQLPPVHAFTDQQVMEIPLAMNPLTQYEVNESESGCQTGSFGWLKPGDPRSELLLSL
CRQVKGYGLSLMLNGGRSGAERRRHRRVGDLIAEGWPSNERQAWICPTAQLLAGNYRVPTFIIHGRQDEVARFESAERFHQEL
QRQEIASGMLQLEHARHIYDLHLKPGMVEWDEQVAPGYNFLFNVVG
>MollE
MSTKVTAPSVAVYCPQSKAPQKEFLDEIRAYILSNPHLQRLAEDAKRLIETWNLVAAKREDIAELSQGPRYMQAISDWLTHGR
SGPVANIMSGILSLPLLVIVQMVQHFQFLEVSGMTHPEFLEGLKNGKGAGAQGYCAGLLPAFAIACAWDEESLITSASTAMRV
ALTIGAYGELGDDKVLDGPTTIVLRLKYEGQGDEIVKGFPGAYVSAVTDPKTISIVGPVLTLEAVSEYARSQGLLVQSMHLRG
KVHNPENMDLAAELCALCDQHENLQLPSWDKMQIQVNSNKTGKPLKEFNPTHEAVYTILATRCEWYTLLQEVAEQLKPAKTGT
DKHTIVLFGIGDPVPLSPFHQARLNITKVEAHRVVKESKLSAYTPSDDSIAIVGASCRLPGANNLEELWDLMSNATSTHEPIR
PDRVPIRASFRAAQDQSFARKDFFGNFVDDVEAFDHQFFKMSPKEVISMDPQQRVLLELAYEAMDASGYLRHHERQRFDNVGC
FIGASFTEYLENTTAHPPSAYAATGTIRAFLCGKISYYFGWSGPSEVIDTACSSSLVAVHRACRAISAGECPMAVAGGVNIIT
GVQNYIDLGKAGFLSPTGQCKPFDGKADGYCRSDGAGLVVLKKLSDAMADGDEILGVITGVATNQGGLSSSITVPHSESQIEL

YKRVLAQSKMQADHVTYVEAHGTGTQAGDPLEIASLREVFGGSDRISNLHVGSLKGNIGHCETAAGVAGLLKVLAMLKQGKIP
PLASHKSLNPKIPALAPSKMVLDKRVEAWDAPIRVACVNSYGAAGSNAALLCCEGPKSYTPSKAITSHPILALSTVPILLSAS
SKDSLVAYAKRLATHLTATPSITLPSLAFTLSQRRKHHRYIFSASVSSLPDLLSTLSSLTPTSPAIHDTTPQPTQKKKPIVLA
FAGQAKQIVGLSQSLYDATPALQKHILACDAALTKLGFPSILPGIFSKAPLSDVVVLQTGTFAVQYAVAQCWIEGGLAVEAVI
GHSFGELTALVVSGILSLEDGVKIIGTRARLMATKWGVERGSMLAVHSSRDVVRDVVGVVGEGEIEIACFNADTSQVLVGSDG
AVARAEELLKRESRFSGIRYQRLDVSHGFHSRFTEPLLEDLERVAGGVTFRKAEIHLETCTTELKKGVLAADHIVQHTRKPVY
FVDAVQRLEQRLGACVWLEAGTDSVIIPMVKRASRESGHVFQGLKFGEGHDSEAILGSVTMDLWKEGVQAAYWPFLSPQESGL
KPVWLPPYVFNKTKAYLENIDRAIEAQKLAASMPALPAGAAAAAVVAPSLLVTPLEKKADSADFTVHIDTKRFTKIVSGHAVR
ARPLCPASMYMECAVMSAQCHHGDSDFSKHALHFENLSFESPLGTDTSRQVQLKLKNTANPTAWDFLLTSSPKAKQGSGKLTT
HGKGSLSLTSQPRLATYQRLVSSRLGEIASQPDAETLMSKRAYGLFSQVVTYADLLKGMHSITMYGNEAVATIRVPDGHAGES
ESTAIDCCDTVALDTFIQVVGLLINSSEHCSPGCCFVASGVDTATLGVGCSFQKPASWGVYATYTMLGETKAVGDVFVMTPDN
TVVGAILGVAFTRLPIDALERMLDSANPSSAPKNAAKKAQPTAVAQVVQDSRVSTPPSRQAGDSGYASDSGLAASPKETGQLK
LAQLIASYTGADPEAIDVNAQIGDLGVDSLAAVELAADITAQFGKEIAATDILETTTAALLKELGASVVPTISAAAAVRAVES
IQSTPAVATPSSSGTATPPTQETSGQRKALYAILEDISGADMTTVSEDQTLRDLGVDSLAVVQLQGDLEAAFSIELESEDIDL
DLTIKQILKLAGISGSDSAPVAAPAAAAASEPAPQVNAYTPAPPTVVKPTPAPEPATPAYLGHLADQLSAAETTLPQAAERCG
FKSYWTVVARRQDEIVVAYILEAFKSLGVDLWAMRASSPLPHIQYLAKHEKVMKRFWQILQKHGIIERGPHSPSSWVRTPVSW
PRTSSSELTEKFIAEFPRYASEARLMALTGPKLAECLTGKADPIALLFTNKTSQAALQDFYTNSPMLATATEFLSEVVRRSIA
ASRGPTVRIVEIGAGFGGTTNRLLETITGLNRKIEYTFTDVATALVSRASKVFGSQHSASTTGVTMDFKTIDIENDPPAAMKG
RYDLAISTNCVHATHDRTTTMRNIKSLLNEEGQMVLSEVTEIVDWYDVVYGLIDGWWYAPDGAYPLQPPQNWMQCFRKVGLNG
TYSNGPTRDLTSQRLLIGAHRPPSSSTVLPRGEIAPKSKVETVVYKVADGVEVEADIYLPLTPPEEAMDIALLIHGGGHMTLS
RKAIRPAQTAHLLSQNILPVSIDYRLCPEVTLIEGPIADVRDAYVWAQKQLPGMVKPMGITVKADNIAVVGWSTGGHLGMTLG
WTHKEAGVSPPKAVLSFYGPTDFESGDLDVRRAEEYPERQMSMANIIASLPKQPITNYATTTIDATNMGWVRPGDPRSELVLS
LFKEGNGLPLLLNGLSSPDSLQTPPDPNKVAYISPLSHVRDGSYTIPTYLVHGTQDEIVPYQTAVKFVAECREKGVECGFLTV
PGARHIHDLEARTGMVGWEEGVAGGYEFLVGKLNA
>DepH
MSDPITASAAFFNPQSRAPSPEDLSALYTYLNGHRHGRTLLRHVSSLSNILPIFGQLRADVRNLPNAEHYAGLLVSWAKGGPS
APVSQARTGIISLPLLLILQLGEYLKYLEYHSISHADFIAQVKDAGGIQGCCGGEPPALSIACAKTEEQVMDNALVLLRIVMG
VGAYIEALDDWTSSESTIIAVRLKYAGQGEELMRMFPHTYISAITEPRSLSFVGNAKSIAALHEYCQANGLPADKMDVTGKAH
NPENAHVVPEFLEILGWHPALFQLPKESRLQVTVRSNRDGVALTDEGIMEDMIPMMLASCCDWYQLLSNVAADIKKTNRTDHR
VVIFGLNDSVPLSPFNRQRLKIFKFKANELLAVKPGQPDLSGPDLPQEPSPPFQFPESSIAITGLSCRFPRANNLAELWELIS
SGTSTAERVPLSRINPAQSYRASQDEVMNKRTFFGNFLDDIKRFDNTFFGINPKEAASMDPQQRMLLELAYEALESSGYVANH
VRTNEDPVGCFIGGSLNEYLTNTSAHSASAHSATGTIRAFMCGRISYYFGWTGPSEVIDTACSSSLVAINRACRAIQAGDCTM
AIAGGVNALTGTVDNFLDLGKAGFLSQTGQCKPFDEKADGYCRAEGAGLVVLKKLSDALREGDKIYGVIPASATNQGGTSSSL
TVPSPSALKDLYRGLFKASGLSPAQISYVEAHGTGTQAGDPIEIESIREVLTDRTRSSPVAIGSIKGNIGHGESAAGVAGLLK
VLAMMQHGRIAPVANHNRLNPKIPPLEQDGFEIPRQVRDWTGPRAALVNSYGAAGSNSALLCCESPNSSPAVVGDNVSIPVII
SAASRASLVDYAKDLAGYLSTHPDLTVAEVAFTLNQRRKRNRFCFETSSTSLPSLIQTLKQVDSPSFELPRQNSPVILALSGQ
FDNKVALDRTLYETYPAFRASIDACNATLIQLGYPSIESAIFQKTPISTALSLQCSIFAVQYASARCWLDGGLKPAAVIGHSL
GEIVALAVSGALSLSDCLKLIAYRASLIDKQWGPERGAMLAVHSSQNDAKRLIARVSSANARLEIACYNAPTSIITAGTARTV
DLAQRILETDPAFAGVKYTRLSTTHAFHSELAETIMPSLGEFAGSLKWNEPSIRLETCTSAPLGSIREWDPARHARDPVYFVD
AVQRLEKEFGSCVWLETGMNSPVIPMLRRAVGDPATHSFHPVSVRDSSGPEAAIGNVVSELWRAGVSASHWAFLTPGAPEAWL
PPYHFERTQHWLENVDWAMQMHEKMASHAETPAAAAPPPAPIPVTQLLSRKTSSNNPALFAINTRCERFQKVVSGHAVLRQPL
CPAPLHIESVTNAIQLLVGDEILSTHDISFSDLFFQSPLGLDPSREVELRLEEAAGDKTWKFTVRSSSSGSWTGPSVSVHSVG
MATLSPKISLSTYTRLVDGAVSSIHGSKSLEHFGATRAYGLFTKVMHYAPFFKGIQSMTVNGDQALASIKLPAAQPGRSESLC
WKRIDAVLIDAFISVVGLLLNSSDDAAEGQILIAVGIEKVVLTAACAADSDQEREWNVYAKFFSAAENQRVGDVFVYDSKGEV
VAFMSGVQFNRLEMAKLTKVLVAANANTVGQQQQQQQVLPVQAAPVKTVPVQTAPALPIQTSTSSSSSASNTIFTPAEDAESE
VEEEEESPDMVIKNLISNYTGLNPDDILSDAVLPDLGIDSLSLIEFSEELNGAFGSELGSDELRDMTLEDLVSRLSKKSNPKP
KPKSQPTEPTLTNGINGAQKEQPVVNGHSHIPAPEAITNGIKPTNPFDALLSTDKHFQSAAKTRGYTDFEFLARPVQDLLVQA
YILEGFAALGVPVLSFSPGSVIPRIPHIPKHAKLVAQFWDILQVPGIVAKQGNGSIILRGRNTPTQRSAADLYEEFTTRFPAY
LPEARLIRLAGENLAKCLTGELDPVTLMFGSPASSRIMEDYYANSPMVSTLTDQLVTFVTELVRGAGGRTVRILEVGAGTGGT
TMRLAQNLDAAGLAGGIEYTFTDISGRFVTKAKDKLKQYPWIKFDTFDLEKDISPTFSNRFDIVIGTNCVHATSSRVQSTRRL
NECLVDDGVVILSEGTYPLAWFDLTFGLLDGWWVAENGTEYPLQNAQKWMQVFHEAGFKSAGFSRGEAPGSEATMQQLLVGCK
KEWPSETDTSPESDLGLSLIPESQTADYRLETLIYKEVDGVQIHADIYLPRNPPNKPLAIALLIHGGGFMMLSRKCIRPAQTK
HLLAAGFLPISIDYRLCPEVNLINGPMADARDAYVWARTQLPSILSLQGLRLDTERVVAVGWSTGGHLAMSLAWTAAEAGMDP
PKGVLAFYAPVQFEDGDLDAHARKSAPRPKQSREAIIKNLGRTPITTYTPNNTTEATGFFGLQPGDPRSDLILSVANPNNGSA
LPLLVNQIDTPGYLDYPPAERIARISPLAQVQGGRYMTPTVVVNSRLDQVVPFAAAERFVSELEKKGVRCRLVGLDGVPHLHD
LYVRPGTQEWEEGVAPGYRFLEGVVGDML
>Pr_MapC
MSLNTEHSKGDLRLLFGPQCSEIEDSIAHIRDAVYKDSAGLGFLSDILDELPSLWPVITSAWPALRKVQGEKQLAALGRRFEN
GSPDSEAEPSSLIMTPVTVMKHLVDFWNLQNVATHPAFPSSPLSRTTAPRIIDSQGFCVGILAAIAVACSQDTREFQSLASNA
IRLAVCIGALVDFDEIVSGKAKSIAVRWETPADHDYLEQTLTKSPNVYISCYTDVNSVTITIPGDTAQRVKFKQELSGRGLHT
KPIPLQGRFHHQQTHREGIQHIMNLCVKDPRFQLPHSNALILPLRSSHNGQVLINAAMLHTVALESILSVKADWWGTVSALLN
SADMEVDESRLLSIGQEEFVPRSARGRLVARSNLDVYGAGVFANGNTSARSAVSLQNGTNTLNGSPQAAEMPPIAITGMACRY
SNADTTSELWDLLELGVCTVEKAPGNRFRMPDLQREPKGPFWGHFLDRPDAFDHRFFNISAREAESMDPQQRVLLQVAYEAME
SAGYCGWQHTELSDEIGCYVGVGSEDYTENVASRNANAFSATGTLQSFIAGRISHHFGWSGPSITLDTACSSAAVAIHMACKA
LQTKECSIAVAGGVNILTNPRVYQNLAAASFLSSTGACKSFDVSADGYCRGEGAGLVVLRPLQDAIDNGDPILGVIAGSVVNQ
GSNRSPITVPDAESQRSLYRKALSLAGVAPDEVTYVEAHGTGTQVGDPIELESLRKAFGNPLRSQSLHVGSIKGNIGHTETSS
GVAGLLKTILMLQKQRIPKQANFRQLNPKVMPPLENDRLVIPVESTKWASARRVAMVSNYGASGSNAALIVRDHTPSLSGQGK
AMAEYIRDMPILISARSEESIRAYCGALRTTLLRHPYSNTVVRELAYNVAMKQNRTLPFTLTFSTSSDPTSLSTRLEAIAAGK
SADIIQKRESNEPPIVLCFGGQNGVTSSISQELYDSCVLLQTHLMACEQAGQKLGLPSLFPTIFTSDPIVNTVYLHFMLFSIQ

YASARAWIDSGLRVDRIVGHSFGQLTALSVAGSLSVQEGIRLVTERARLIQSNWGPESGVMLAVEGTQAEVQRVLEQTGHRAE
IACYNGPQQQVLAGTGECIRAVEDALATNPLTSNVRVRRLENSHAFHSRLVDSIVPGLTELAESFVYQAPAIPIEACSATGDW
SIVTPAKIVEHSRMPVHFQRAVERIAQKLQVPAVWLEAGSASPIIPMVRRVLEKSSATHTYHRVNLDGSDGSGNLATVTSALW
GQGVHVQFWPFHHSQRGTFGWMNLPPYQFAKNRHWVDFDPTAFSFSGSSAEPQCGSQERAGLLRKLSDGPEEYLFAVNTQDVL
YRSCTKGHAVLDQTLCPASMYMEMVLRAATSVFTLGESSTLTMSHIEDLVISSPLVLDPQGSVFVRLIPEAVASSQTWSFSIF
SSSGTGNESSIHATGSVSLCNERSRALSHFQSMNRLMDPARARGIEDHLASNGLKGSTVYSALEQVTNYADYFRGVRQVFANG
REAAGLVSMAPSATETTCNPILLDNFLQVAGIHVNCLSGREAEEVFVCNAIGETYVSDSLFKKEDGAIPLSWKVYTNYVRPSK
NEIVCDIYVMNSQGDGLTAAIMGVRFMSVSIRSLTRALAKLNNNFPDVPQLPPTIQPAIVTADYDEASDNVNVDSDLVAVQEM
LCELFGVSVEEVSPSVSLIDIGVDSLISTEVLSEIKRRFHKDISYSTLVDIPNIQGLTEHIFPGHSHLAPSQIVIKPVRQQTV
IPQTVTSLPVPANAGPSLVSVAHQCFYETHAAVSHTHNADWAGFFNAIYPQQMTLITAYILEAFRALGSPLESSQADEVLPII
SVLPRHEQLKKHLYTILESVNLVRQTPTGQLVRTATPISPLSSHALHAQIRDEHPPYALEHDLLQITGSRLAECLTGQADGVS
LIFQDSQTRRLVGDVYTDSPVFKSGNLYLAQYLTDVIQTLGNGRQVKILEIGAGTGGTTKNLLEQLSALPGMATRMEYTFTDI
SPSLVAAARKKFSKYDFVRYETINVESSPPSLLHGQYDIVLSTNCVHATRNLVESCSNIRKLLRPDGILCLVELTRDIFWLDL
VFGLLEGWWRFEDGRKHALATEDLWDQTLRQSGFEWVGWTNNEAVESNALRVIVASPTKAPSALEICSKPANMETVVWGERNG
LQLLADIFYPDVVDTTQKRRACALMVHGGGHVMLSRKDIRPAQTQTLLDAGFLPISVDYRLCPEVSLSEGPMADVRDALGWVR
RILPNIPLLRPDIRPDGNQVVAIGWSTGGHLAMTLAFTAPAVGIAAPEAILAFYCPTDYEDPFWSRPNFPFGQTVASNDIEYD
VWEGVQSAPIKGYNPAFKERPLGGWMSTSDPRSRIALHMNWTGQTLPVLLGGMHKEFRIPDELPRPTDEEIQAVSPNYQIRIG
RYRTPTFMVHGTSDDLVPCAQTESTYNALTQNGIEADIRVVQGAIEVLLYLQHPERARKWICSGSLTLEI
>Pb_MapC
MNFHKGQPKEDLRVLFGPQCPDITDSITHIRDAISKDPTGLGFLTNILDELPSLWPTIAGAWPALKNVEGESQLLALGRLFEH
ESEDRVEASNLMMTPITVMRHIVDFWNLQDVATHPAFPSSSLSETEMPRIVDTQGFCVGLLAAIAVACSRNTQEFQYVASNAI
RLSLCVGALVDLDEILCGSTTSLAVSAERVKQEIHDHGLRTKQLSLRGRFHHEAHREGIQHIMKLCTNDSRFKLPRSDALLTP
LRSSQGGEIFQQEALLHTVALDSILCAKANWYDVVSALINSTEMTVDQSHLLSIGPEEFVPRSARSRSVARRELQSYAMQGFS
NESPQPSTASLSNSVQTFDSRPQAAEASPIAITGMACRYPNADTLAQLWDLLELGRCTVKSPPESRFHMSDLQREPKGPFWGH
FLERPDVFDHRFFNISAREAESMDPQQRVALQVAYEAMESAGYLGWQPNGLSRDIGCYVGVGSEDYTENVASRNANAFSITGT
LQSFIAGRISHHFGWSGPSISLDTACSSAAVAIHLACKALQTNDCKIALAGGVNVLTNPRVYQNLSAASFLSPSGACKPFDAS
ADGYCRGEGAGLFVLRPLQDAIDNGDPILGVIAGSAVNQGSNNSPITVPDAEAQRSLYNKAMSLAGVSPDEVTYVEAHGTGTQ
VGDPIELDSLRRTFGGPQRRNSLHIGSIKGNIGHTETSSGAAGLLKTILMLQQQRIPRQANFNQLNPKVKSLTPDRLVIASES
TEWASTERVAMVSNYGASGSNAALIVKEHAPIRSEQNGTAPEYIQNVPILVSARSEESLRAYCGALRATLLSHPPSETLVQKL
AYNLAMKQNRDLPLNLTFSTSSDATSLSARLEAISTGASADLIQKRPSNEPPVVLCFGGQNGLTATISKEVFDASALLRTHLE
DCEEVGRTLGLPSLFPTIFSSAPITNIIHLHFILFSIQYASAKAWLDSGLRVSRIVGHSFGQLTALSVAGSLSVRDGIHLVTE
RARLIESSWGPESGIMLAVEGTDIEVQQLLDQTGHIADVACYNGPRQQVLAGTAESIAAIENAAARTPSASKLRLTRLQNSHA
FHSRLVDSIVPAIMEVAGSLVYQTPIIPIEACSASGDWSTITAAEIVEHSRMPVYFRRAVERVAEKLQAPAVWLEAGSASPII
PMVRRVLESSSVANTYHKIDLGGSSGAQNLANVTSALWAQGVHVQFWPFDRAQHGSFKWMNLPPYQFAQNSHWVDFDPAAFSS
AGPSSGKQSAGQEAGLLCQLSESPDERLYHVNIQDALYRACTQGHAVLNQTLCPASMYMEMVLRAAASIFPTGNASEPAMSHI
EDLTISSPLVLDPQGDVFLRLTSDGAGPTRPWLFSIFSSESNDHTSVHAEGTVCLHQERSRALARFQSMDRLLDSARSKTIEA
DPASNGLKGSTVYAALESVTNYGDYFRGVKKVFANGREASGLVSMMPSASETNCDPILLDNFLQVAGIHVNCLSDRRSSEVFV
CNAIGETFVINSLLKQKNGASPSTWKVYTSYVRPSKTEIACDIYVMDCQTDTLSAAMMGVRFTSVSIRSLTRALAKLNNNVLE
TAEAQSVVEPAIPAEKSVVTATPSAPAADGGGAKDLATVQEMLCELFGVSVAEVSPSVSLVDIGVDSLMSTEVLSEIKKRFQV
DMSYTTLVDIPNIQGLVEHIFPGHSHAAPSQPVVETAPVQSVAPQAVSHVPTPANNGPPLVSVARQCFDTTHAAVSHTSDAHW
TGFFHTTYPKQMTLLTAYILEAFRALGSPLEASEPNEVLIPISVLPRHEQLRKHLYKILESVGLVRQMPTGELVRTTTPIPLS
QSHDLHTQIRAEYPPYALEHDLLQITAPRLADCLTGKADGVSLIFQDANTRRLVGDVYAQSPVFKSGNLYLARYLLDVVQSFG
SSRTIKILEIGAGTGGTTKNLLEKLSTIPGLSTRLEYTFTDISPSLVAAGRKTFANYNFMRYETLNVENDPPSALSGQYDIVL
STNCVHATRNLRESCTNIRKLLRPDGILCLVELTRDIFWLDLVFGLLEGWWRFEDGREHALATEMMWDQTLRQSGFEWVDWTN
NETVESNALRVIVASPTGNSSTATMSPSKLTKMETVVWGERDNLQLRADIYYPETVDTTRKQRPIALMIHGGGHVMLSRKDIR
PAQTQTLLDAGFLPVSIDYRLCPEVSLAEGPMADARDALSWVRRVLPNIPLLRADIRPDGNQVVAIGWSTGGHLAMTLPFTAP
AAGISAPNAVLAFYCPTNYEDPFWSNPNFPFGQTVASNEMEYDVWEGLQSMPIAGYNPALKERPLGGWMSTRDPRSRIALHMN
WTGQTLPVLLKACTIKGNTEKCSPDDLSRPTEEEIQAVSPNYQIRVGRYNTPTFLIHGTSDDLVPCAQTESTHGALTASGVEA
ELRVVQEAAHLFDLYPASHAGQEAKAAVAEGYEFLRRHVQL
>FncE
MGSLPETVVLFGPQRSDVDDSVCKIRRALREHPGLHFLGDVVDELPSLWPTISDALPGLTQIPGETMLVTLARSIREEDAPTT
EIDPLNMLLTPLTVIRHIVEFWELGQGLDHPAFVAEASQRSRVTDVQGFCVGFLAAVPVACCQSLKEFAVLTAAVVRLAVCVG
AAVDLDEAMHGASASIAIRWKSPGEYESLQQILARHPEESYISCVIDVDSVTVTVSSDVEQLLTQELMDHGLILRPIALRGRF
HHESHADAAESILELCDRDSRFQLPSGSAACLPLKSNTDAQGIAQDMLHTIAVRSILTRQCNWVRTLSPMLGQLQNNKPQRSV
MSIGSGQFVPRSIRTRLQHTNHFSQRNRAPAAPLIAITGMAGRYPHADSLEELWQLLELGKCVVQELPDSRFKVRQLQREPRG
PFWAGLLQRPDAFDHRFFGISGREAESMDPQQRLLLQVAYQAMESAGYCGLHAKDVPRDVGCYVGVGSEDYTPNVASRHANAF
SATGTLQAFISGRISHYFGWSGPSVVVDTACSSAAVSIHLACKALQTGECSIAVAGGVNVLSNLTVTQNLSAASFLSPTGASK
AFDAAADGYCRGEGAGLVVLRRLDDALSNGDPILAVIMGSAVNQGSNSSPISVPSSDSQRALYEQVLSRAGVAADEISYIEAH
GTGTQVGDPIEFQSLRKVFGGRHRAEKLFVGSLKDNIGHTETSSGVAGLLKTVLMMQKRRIPKQANFTRLNPKISPPPEHDRM
VIPTQSSPWEPKQRLAMISNYGAAGSNAALVVKQYCAPGRKLQDDDISLPSEVPVFISAKSAESLQLYCRRLHAFIKDTPTLE
VLESFVADLAYNLAVKQNRDMDFCIAFATSSASPAALLTRLESVFSSLVADIQKAPKNAPPVVLCFGGQNGNTATISQQLVNS
CELLRFHLMECERSCQLMGLRSLFPTIFQADPVEDIVHHHCMLFSIQYACAKSWLDSGLQVARMIGHSFGQLTALCVAGSLSL
SDGLRLISERARLIQRNWGAENGVMLSVECTTEELQRFLRDAENTVDVACYNGPRNQVLAGDETSIRAIEERINAQSGRIRMK
RLTNTHAFHSRLVDPIVPGLMEVAQSLQFRPPSIPIEACSMDEEDWSSSGPSASNIVQHSRMPVFFQSAVERTLQRLQGQIVW
LEAGSASPIIPMVRRVVEASSARSVPHVYRPLDLGSPQAPATLAKATCELWSNRVSVQFWPFQGAAQRRSLNWMSLPPYQFAE
GNHWIDYDPSAFTAPREVVSETPSEQPAGLMRLLESNPHGESLFQINADDPLYQSCTKGHAVMDQSLCPASLYVEFVVHAASC
IFDDDVSSALPHLQDMAISAPLVLRPQGSVFLRLSRTKAGHPEWSFLLFSRKEDDSSVTHASGMLSLHPKNKPSAIKARLHSL
NRLVNPSRGAQIEASPESSGLKGSTVYQVLQKVTDYADYYWGVQKVFAQGHEAMGYVSRPEHSLSRKSACDPVLIDNFLQVAG
IHVNCLSQRRDDEVYVCNGVGDIFGELLIRAEHDETGAWDVYTNYERPSQNQVSCDIFAIDRKGSGLVLAIMNVVFKSVSIRS

LIRTLANLNSPGEARPEPAPTWTDELAAKLPQKTVPQTPVLGADNLSAVQTMLSNLLGVGMEELVPSSPLGDIGVDSLLAIEV
LSEIKNRFQVNISTATLVDIPDIQGLVECIFSGFPTPESTQASTPTGPGPVQRQENGVDTEDPSTSDSSYNLTNFAGQCFDPV
REDAAPLYDTQFVGFLRSVYPKQLALLTSYILEAFQSLGVQFESLAPGEAVPEISVLPRHEKLLRQLYSILERSHLVSRTASG
TIVRTISPVPTTPSGILHNELISEYPQHAIEHQLIRVTGPHLAQCLTGETDGLSLIFKDADTRALLENVYTNTPIFKFSTLHL
VRYIADILGQLGDGHDRKLRILEIGAGTGGTSKYLLNHLTETAGDSRHPSIEYTFTDISSSLVALARQKFAMYPFMKYATLDV
EKSPPSTLQGKYDIVISSNCIHATRDLGESCRNVRRLLRPDGILCLTEFTREVPWLDTVFGLLEGWWQFADGRKHALASERLW
EQTLRQAGFQWVDWTGNEMEESQLMRLIVASPSVPQLDSPPNITQQTVVFAERDGLELQADIYFPSDADSDRTRPVALMIHGG
GHIMLSRKDVRAAQTRILLDAGFLPVSVEYRLCPEVSLIEGPMQDVRDALIWVRQTLPTLNLKRRDIRPDGERVVAVGWSTGA
HLAMTLAWTSLDSSIIRPPDAILAFYGPTDYEDPFWSQPNFPFERDLAVPAIPDYDLLEGVYDQPIIAYNPPVEKRALGGWMS
PTDPRSRIALTMNWKGLTLPVLLYGWQWKSQPEGQRRVEDLPQPSKDEIEAISPLAQIRSGRYKTPTFIIHGTLDDLIPWEQA
KRTYEAMVENGVDAGLRIVDDAVHLFDIQPRGRMKDGELRAVRDGYEFLAQYAVL
>Esp3
MINTFTPPSLVVFGPQTPHPTADQLERIRATLLNNAHLQSFAAAIEDLDSFWELLVNADPNLANLAGSEQLHLLRTWLDQGTF
PPTSSNPLSNAVLTPLTVIVHVVEYLDSLNDQALGCSHSELLQSVTDAGIQGFCSGLLTAVAVACSSTESNLIRLACVSLRLA
VVIGAYVDLDESSGELEKEAACLAVRWTNPEQERSVRSSLEDTPEAYISVVTSADTITVTLDARSVNQFKQAIATAGASFTTI
PLRGRFHHSDHTEAVKQIRTLLSSVPGLQYPADSLPLASIRDGSGHLIAQGQSLEDCVVTSMLLNLSDWHATITRSIACISDR
SPGQKAQVLALGLNECIPRSLAAEHELEVRNVLAVKSAKAGHNTRSDISHALPEDAIAIIGMACRFPGADDLEEFWDVLQSGS
TMVGTLPKDRFETIGLRRSPETDKVFRGNFLRDGFAFDHRFFGKSSREATSMDPQHKLVLQVAYETLESSGYFNKATRPTDVG
VYVGVAASDYEDNVASHSANAFSVLGMVRAFNSGKISHFFGFDGPSLVFDTACSSSAVAIHTASRAIQTGDCSMALAGGVNVI
TSPILHQNLAAANFLSPTGASKAFDSKADGYCRGEGSGLVMLKRYSAAVADGDEILGVLAGSAVNQNDNSAPITVPVSESQSH
LYRTVLKSSGLSAKEVSFVEAHGTGTPKGDPIECASIRQVFGRHPDRKLHFGSVKGNIGHTEAASGVAGLIKTLLMMERRLIP
PQASFQSLNPNIPPLELANMEIPRSIKKWDAKRMVAFVNNYGAAGSNAALAVTQAPKRIDSKHYTDTLTQEDLSVNSAVYPVM
ITAKSAASLQAYAQALEDFLSHHRYIDQSKLLADVTYSLAYRKARDMPYSFGVKVKSIPELRQQLNTCKSQPLCAPSAPPQDK
PIVLVFCGQTDTTVKFSKDAFNVLPVVQKHLRQCDTILQSKGLNSIFPTIFQNDPISDVVDLNMALFSLQYSMAMSWLDAGLR
VHTIIGHSFGELTALAVAGYLSLEDAIHLIAGRARLMQTEWPAERGAMLAIDATKSKVLELIASAQQEHEDAYLEIACYNGPS
SHVVVGSKAAIQALEEITSSSSPPVRSSALNVAYGFHSAFVDSIMTEYTKVVSEVVLRKPKISVQLCSSNEQSVKRHISYERL
ASQSRRPVYFSDTVGRIEQFYGACRWVEAGVGTAAMALVRRALQGSSVDGHAFHSVKLSDDDALKPLADLTLDLWNAGVKLQY
LPFVSPPYEARFQLLNLPPYQFEKHTHKLEYIDRHESAETKLIDNELSNQTPTLVSLAKYQSSDQRSAFFNVDQSVEDFVVSI
EGHSVLGNPLCPVSLYVEVATRAAKLIAVDIDPSKYTPRVENLVISAPLGRDMDRKIDVLLEGLDGEKNAWHFRVSSSPRDSD
GSTNHATAVIRLCSTQTTSLEPRMKQIRRLVDYDRVNAILADPAAAGVHGSMVYKLFDRVVNYSNIYRGVTRIASKGNEVSGI
VSMPISVSSALRETSCDPLAIDNFTQVAGLHVNGLDDCNEDEVYICSTVEELYDLRDAKRTGHNSWLVYSICSGNGGKELSND
IYVFDPDSKTIAVMILGVKFHKTNINALKRVLSRANASGTAPITTKSSPLAADKTRSIRRPTLPYPKQKQAEVYSRPKPTAGI
RSQVAKLLNEVADIPIDDLTDTAKLEDIGIDSLMATELLAAIRKHFGIDISTGEFESIVDFKSLCEALDGSGGSDNSSSETDS
GSESVETSATTPLSESIGETFQNDIIYKLATLVKEHLEFDGELFVDTKLGDAGLDSLMGIELGNDIQKQFGAAIDVMKLDSDT
TFGQLSELVFPSSRDKTAQSLSEELLRVKILGGNFDEETPKETLQYRIDCVEESEIVMQSPDMNLNGIDEDFRTIRSDYSRFA
EETGWAGFRTKVYPQQQQLVLAYVLEAFSELGCNLSSLKVGDFISPVPCLPKHDKVVSQYLAVLRDASIVARKGDGYVRTNVA
VPRITSAELLEKMLISFPQHSSELKLLHSTGSKLAKILAGSIDPIHIIFRTKADRDLLEDVYTNSPMFATGTKVMGNFLQQAL
KHRGSGKLRILELGAGTGGTTKHMVEILSSLGVDFSYTFTDLSSSLVAAAKRKFSQYSGMKYTVLDIEKNPPEDMLGEYHIIL
SSNCVHATKSLLVSSTNARKMLRSDGVLCLLELTKNLYWLDCVFGLLEGWWLFEDGRQHVLASETLWQQTLLKAGFKHVDWSD
DASEESDQYRVVVGFASAASHGAGSVHVAKETVEFQRVGNTVLEADIYYPRKPDDGEKRRPIAIMIHGGGHIMLSRKDVRPKQ
TRLLLDRGFIPISIDYRLCPEVTLTEGPMADVCTALEWARNSLPTMTLSRPDVRPDGSKVAVVGWSTGGTLALTLGWTAPQRG
IAPPNATLAFYCPSDYESDFWKSPNFPENTTPADAAVDYDLLEGIRDQPFTAHKVPRDQGAVAGWMTLKDPRSRIALHMNWKG
QSLPTLLDGLPCGSTVDPAEAKQYLSRPQPSSERIREVSPFAQVALGNYQTPTFFVHGTLDDLVPCDHTEKISAGLAARGVAT
GLSIVEGAHHYFDLYPETERRFRDAVARGYDFLCDQLGMV
>AstE
MRLCTECREGSLRLLFGPQCTEIGNQITRIRDALENHPSGLQFLHDIVNELPSLWPVIVGACPALDRVPGERGLTALAQLVNN
ESSASPVGDQLNIVLTPLTVMAHIAEFWQLQNVAAHPALPSGSSITSPAALPRIIDVQGFCVGFLAASVVASSEDAREFQSTA
SNAIRLAVCIGALIDLDETVSGEATSIAVRWESQESYEHLQQILTQEPNVYISCETDVNSVTITLPDPAAKWMKQQLNGFGLR
SKQLSLRGRFHHPEAHDQVVRHIMQLCSRDCRFELPNSDTLPFPLRSNKDGEVIEPGTMLHTVALKTILCERANWWKTISSLL
TDWELGVDESRLMSISTTEFVPRSARGHLVSQGSFPTACRKHGPVNGHTPSAPTTSFLKEEDNVDTRPQNAQVAPIAITGMAC
RYSGADCVEELWDLLEQGQCNVQRVPENRFRMSELQREPSGPFWGHFLEHPDVFDHRFFGISAREAESMDPQQRVLLQVAYEA
MESAGYCGWQKTKLTQDIGCYVGVGSEDYTENVASRHANAFSATGSLQSFISGRTSHFFGWSGPSITIDTACSSAAVAIHLAC
KALQTKECSIAVAGGVNVLTNPTVYQNLAAASFLSPTGACKPFDAAADGYCRGEGAGLVVLRPLQDAIDHGDPILAVIAGSAV
NQGSNNSPIMVPDAQSQQTLYDKALSLAGVTPEQVTYVEAHGTGTQVGDPIELDSLRRAFGDRHRRQDLFVGSIKGNIGHTET
SSGAAGLLKTILMLQHRRIPQQANFSILNPKVTPLDHDRLIIPVESTKWEAEARLALVSNYGASGSNAALVVREQITKQGHAT
PRYLLDVPILISAQSQDSTRAYCGALRSALLSNSITVQDLAYNLAIKQNRGFSFISAFPISSHAESLGARLEAIVTGESADIL
QKRPASEPPIILCFGGQGGQRPCISKTLFDNCVLLQKHLLECEKVGEMLGLPSLFPMIFASDPIADIIHLHFLLFATQYACAR
AWLDSGLCVNRIVGHSFGQLTALSVAGTLSLRDGIFLITERARLIQARWGADSGAMLAFEGPKTVLDELLTQTGHCVGIACYN
GPQQVILTGTEESIRTFENSVEGNTSDNNIRLRRLDITHAFHSRLMDSIMPDLMEVAQSLTYLKPAIPIEDCSMNGDWSIVTP
AKIVEHTRRTVYFQHAVERIAQNLRGPAVWLEAGSASPIIPMVRRALENPSELHTYCKVDLRGPDAARNLATVTSDLWAQGIH
VQFWPFHRLQADAFSWLNLPPYQFAKTTHWIDFEPAAFSSAPSKVSNAASQQRTGLLHQLSDGPDEYLFAVNTQDPLYRSCTQ
GHAVLDQPLCPASMYMEIVLRATACLSSLHATSTPALSHIDDLTISSPLVLDPLGDVHVRLSPEGPSCSQVWSFSIFSNSEQA
RDTVTHAKGTVSLLQDHSSSMASFHSIGRLVNQSRARAIMEEPTSSGLKGSAVYSALRQVTNYADYFRGVREVFANGCEATGI
VTMAPLTTENTSNPILLDNFLQVAGIHVNCLSDRQEDTVFVCNAIGKTFIGELLFRTASEALLPSWTVFTNYAQQSKNQITCD
IYVKDSVTNGILVAMMGVFFTPVPIRTLARTLAKLNNNSLKDAKPVAICSVPPQTQSPDDMPAHERATAEINVDRDLAALQEM
FSDICGVDTDELSPDTSLIDIGVDSLLSTEVLSEMKKRFQIDITYSTFVELPDIQSLAQHIFPGRSKAALSRPVGDEIVPNGP
AMSHAGPDVTMDASDEPSLAIVAHQCYEATRTAVSHTDDAHWTNFFHSVYPQQMRLITAYVVEAFRLLDCPLESYQEGEVVPT
ISVLPRHEALRNHLYVILESVNLLCRTPEGKLVRTATPLSPLSSHGLHTQIRSEHPAYKLEHDLLQITGPQLANCLSGRADGV
SLIFRDAQTRCLLEDVYTHSPVFKSGNLYLARYLMDVIQRFGNNRPIKILEIGAGTGGTTKFLLDQLLNLPGATTRVDYTFTD

ISLSLIAAARKKFANYNFVQFNTLDIEQEPPTRLHGQYDIVLSTNCIHATRSIAQSCSHIRTLLQPNGILCLVELTRDIFWLD
LVFGLLEGWWRFDDGRQHALASEQLWHQTLHQAGFDWVGWTDNETVESNALRVIVSSPSGVSGLTQGISTKPVKMETVVWARR
SDLELKVDIYYPNEVDTLRTPRPIALMIHGGGHVTLSRHDIRPSQTQILLRAGFLPISIDYRLCPEVSLVDGPMADVCHALNW
IRNTLPSLPLLRPDIRPDGNRVVVVGWSTGGHLAMTLPWTAPAAGIAAPDAILAFYCPTNYEDPFWSRPNFPFGKTVAPNDME
YDVWEGVRDTPIVGYNIPPQERPLGGWMSTRDPRSRIALHMNWKGQTLDMLLKGWRHKDKADDIPRPTEEEIQAVSPHYQIRT
GRYGTPTFLIHGTMDDLVPFEQTESTYDVLVQNGVEAEIRVVREALHLFDLYPSFHASKEARDAVADGYEFLKRHVRL
>ATEG_03629
MTPTLLLCGSQAIQWSEDYLSSLREMLLADSALQPLVHAIRDLPQLWATLLEADTALHKMPGKQTLDRFTRWLDGERLLEKDS
PSDLNMIMSPLTVIMQLVEYISHLHQSNLTHLQILDGAKHGGIQGFCTGFLAAITLSISRDESDVAELGTVALRLATCIGAYV
DLDQCNSSGFACLAVRWPTAADERKVKDILETYNGAYLSVRSDVASATLTVPRAAKSSIIDELSNIGAHVKDIPLSGRFHNQV
NRELFAKLAALCKSTIGLQFPGHCRPLVPLRSNADGELLSGNEALHVAALNSLLLHVSDWHKTVSKAMDSLSQTTAEPEVSVL
GLGDCIPRTIRQSRALHVSHIKTGSTQSHDDPYQYPGDSIAIVGMGCRFPGADSLEEYWKVIESATSMLGDLPEGRFPKTNLR
RDPNGKIPLNGNFLRHPDLWDHRFFKRSSREAASMDPQHRLALEVAYEALESAGYFAQRSPAKDIGCYMGVAASDYEDNVASH
APTAFSVLGMVRAFTSGKISHFFGLSGPSLVFDTACSSSLVAIHTACRALQANECSMALAGGVNVITSPTLHQNLGAANFLSP
TGGSKSFDDRADGYCRGEGAGIVLLKRLDRAIAEKDRILGVIAGSAVNQNDNAYPVTVPVSMSQTALYRRVLDMSGLSPRPVS
YVEAHGTGTPKGDPIECASIREVFGGQVNRKLYFGSVKANIGHAEAASGVAGLIKVLLMMQKRSIPPQALFASLNKSIPPLEP
DNMAIAQRVTPWSEEFYAACVNNYGAAGSNAALIVTQPPNIRRGSHAGVALKNCPILLSANTAGSLRQTTVVLREFLAHNRAI
SENDLLKSTAFHLAKRFNPSFKYRHSFSVASLNQLDEKLQICSQLPDSEFLLPPNHRPVVLAFGGQTGNVVHLSEGVYRGSSI
LRKYLDKCDMQLRQLGLTSIFPTIFEQKSIEDTIQLHCTMFSLQYASAMAWIAAGLQVQTVIGHSFGQLTAMCVAGVLSLVDA
IRLIAGRATIIQEKWGAERGCMLLVQGELALVQKLISQAREATSHVVEIACFNGPNSFVLVGSEADIDAFDGLAASSLKTRKM
AVTHGFHSRFVDTIMDDYQKLADSLEYKSPTIAIETCSSGETWDMFTADKVAKQSRQPVYFAEAVERIAQRLGSCTWIEAGSG
SGITSMARRALNDTTNHDFHAVNLGGPEPWAAFADTTVSLWQAGVQVDYWPFHKEQQLEYLPLNLPPYQFERSRHWLAYVDRP
GADGLIQSKETQSVETKPKLVSFVKYLDSNRQTAEFSIGQDCEQYQALVRGHAVLANTLCPAALYVEMAYAASLLVPDFSPS
TYTSRVEDLHMQSPLAIDLKRGLRLVLSSSGSGTWQFIMQSFSLSDSDNATQHASGTVNISSLTSEKLQSRFSRYKRIVNYER
CESLLSDSGTSAIQGSLVYKMFDKVVVYSDIFRGVSKIASRGHEVTGQVSLPSAGLELVKDSVCNPLVVDNFTQVAGLHVNSL
DDCGSNEVYLCNGIEQIDACKPLDASGSWLVHSSFDRVGTRELVNDIFVFDASTKELVMTLFGLRFAKVPTASLKRALERANT
VQNPVQTPSLKVTEPSANVPKAQPVSTYPKPMKPAPAADAQIRTATMALLNEVADVPLSDIADGAQLEDLGIDSLMAAELLSA
IRERFNLDIPTSTFASIVDFKGLYQHIASGTDAGILTPSSSGMESDDSILEVQYTDTSTPFSEIAYPLEDKDAGDSAQAGQIA
QLSQLFAEHLECPLPIPSGETLRDIGLDSLVGMELAADIQQAFGRKVDLATLDPECTFGQFCDMVIPKPTLSVPTVSEKVDKT
VRWASTEIAYTAKRENKMQDPVEMQSDGGNVNYLAHCAEDFAQIRKNYTTFAKQTGFADFRANVYPQQKELVCAYVTEAFAAL
GSDLKTIPSGSPLPPIQHIARHAKVMKQYYKVLEDSGLITITDNGPIRTAKPVSPVKSEDLYQMIYSAFPQHRGEHKLLNSTG
SKLASCLKGETDPLQILFGSKASKDLMEDVYTNSPMFATGTRILGDFFVKTFSKYKGPEKLRILELGAGTGGTTKYIVEKLLE
HNIPFTYTFTDLSPSLVALAKRKFSHYGCLEFLVLDIEKTPPEHLTNSYHAILSSNCVHATKNLLNSTTNTRKLLRADGFLCL
LELTRNLFWLDCVFGLLEGWWLFEDGRKHVLADEYLWKETLLEAGFRHVDWSDDDTEESDQFRVITGFVADIGHNALDQAKPV
TTKLPTMETTSFATVDGIPLLADIYYPTKPDAPGVKRPIALMIHGGGHIMLSRRDIRPKQTRLLLERGLLPVSIEYRLCPEVS
LTEGPIPDACAALNWVRTVLPTLRLQRPDIHPNGDKVAVVGWSTGGTLSMMLAFSAPQRGIRPPDAILAFYCPTDYEAEFFRT
PNYPEDTSEVVPEIYDILEGVQERPITAYNVPAHQGATGGWMSLSDPRSRIALHMNWRGQMMPVLLDGLPSKKTLLEAGGDAS
PSKWMDLPQPSVDRLRAVSPYAQIVQGNYRVPTFLVHGTRDDLIPWEQSVRTKDALTSQGVAAGVAVVDDAVHLFDLYRDPEG
RYWNAVLEGYEFLLRHL
>SetA
MSTRLPAGVFCGAQSIPSSDICESIRLCLRKDPGLQHLAQTVVELPALWSRLTETHAFCDAAVGDPLKPLSDWITSDNPRSLT
IPESLPNVLLAPLTVVLQLVQYTQYLREQHHAAISHEQVLQSVSSPSAGVQGLCTGFLTATALACSRDLAEVNTNGEVAIRLA
MCIGAAVDADERKREGGNRTQCLVAHGSPHLDCREVMAALEQYPRVYVSVKLDAHRVTITAVDEDVPSLSQDLIQHGIKLKPL
SLKGRYHNAVHEEIVAALKEVCNAEPGLRFPEESKPLVPLRCNTTGDLVQPGEILHDVCLRSLLMEPADWYSTVTASIAGMTH
SGSENALSHTVLELGFVPCLPRSLVTRSCLDVLSASLDADDRPFSYPDHSIAIVGAAGRFAGAESLDQLWNLIRTGSSTVSTC
TQDGSMPVRDSQTQTGNYLSNVDAFDRGLFGYSPNEAQFMDPQHRVALQVAYEAVEGAGYLSPGSCPRKDIGCYVGIATSDYE
ANVACHRASAFSFTGTARSFVSGRISHYFGWTGPSVAIDTACSSSAVAITMACKELMTKGCKMALAGGVNIISNCRTTRNLAA
ANFLSASSGPCRSFDEAADGFSRGEGCGFILLKRLDDALAENDQILGVIVGTATNQNINCDPITVPVHESQVDVYQRSLAASG
IDSLRVSYVEAHGTGTTKGDPIEYHSIRQVFGGRNERSQPLYIGSVKANIGHTEAASGVASVLKVLLMMRHGELPPQGNFTRL
NPAIASLESDQISIPTALQPWKSNFRVASINNYGASGNNTAMIVCQPPNVLQHPKWQEDTALRYPFLLSANTKTSLRKMCESL
QQFIALALQRTARRPLRLADLAIWIGSKQNQRLRYRIAFDAGSIPELQDHLHRYITDDGGTAVIPGPSSTTARPVVMVFAGQT
GNTVHINKDAYNCSALLKSHLDRCDAVLRETGRPSLFPNIFQSKPEADVSLLHCMLFAAQYSCAMAWIDAGLPVQRLIGHSIG
QLTALCVAGCISLRDAIALIAGRASLIKEQWGSETGVMLAVNMDRLSTETLVRLVAEGDPDCAVEIACFNAPARHILVGTEAA
IRKVEGLLSEYGNPRRLKTTHGFHSRLADSIMRSYEAIAQSISYHEPTIPIETCSPGSSWARVSAELVAQQTRQPVYFVDAIR
RIEKRFGPCVWLEAGTGSSGITLARHALQNSQSHSFHAVQLASSEPIASLQHTTQQLWSEGMAVQFWLYHQIGRSSSCLSWCV
PGYQFDESRHWVSYKEPEKAAVEYEDSHTGSACTSLITLITPPAIGGDQSQVAKFSIDTHNEEYQGWLSCRVVLGSYLCSIAS
YVELAARAAMLLSRGLEHHHCLKVSNLSLCAPLGIQVDSPLTLTLRSTTVTSEWEFSIDSDIQHAKGHIGIMPMPSTSALSSA
LQRLIDYRYCQRILADPDASSLRGSLAYKILERVVECAPSYQGIQSTTIRDLEAVAHVILPPAAHSLQGQGQEKSACNWQTLD
HFILAAEMHILSQKECSHHDVFVCSELTEIIPFVTAPLDGRGPWMVYTQLSWANEHTILCDTLVFDTATQQSIMALLGARYTR
VSTSSLQKAISLASSHQEQQHQQNMLPVSPCHQPVTTARPLAPLIPPTPVTPTGEAVEEKLKYIVSDLTGIPPSQLSPMTSLA
ELGVDSLTTIELRRSFKESFQVDLPNDIDNGHYTIGDLSAAAAAAAAVNLSQLQPQLSGKLKLCQEMFLPPTGLITPSSGPTT
PSSPGGGRTNTSETLKHSSVCEIITRLIRIVAAQLNCPDKIVPSIQLQHLGLDSLGAMDLEAELAKAFGRKLNLQKMSPASTF
GDLMDLVLSSHGQPSLDQICTPTGISTASGTGSPSILLSNPRFLFEALSWFAGIRQTYSQFARMAGFSGFYTHVYPRQMRLLT
SYIVDAFGALGCKLPDIPAGQLVPRIAHIPRHEKLVDHYYRILKDSGLIQRDKVNGSTWIRTSNAIHPAPASETYQAVWTDFP
AYRPEHELLHCTGTRLADCLSGQADPLYLLFQDPKAHELLEKVYADCPMFNTGTRLLSHFLHQVAPPSSPEPLRILELGAGTG
GTTKPVLEQLRSRKINFHYTFSDISAALVARARDQLAGYQGPGAAIDFTVVNIERPPLHEHVQAYDVVISANCVHATKDLGVS
CANICALLRPKGMLCLLELTRDVYWLDGVFGLLDGWWRFDDGREHALVDERQWKDCLQRAGFAHVDWTDDESRESDVFRLIFA
MKQ
>BrvA

MSAPSSSPALPSIVICGSQTIPPRSQLLDELGHQLRSDPEILGLKLAIIGLPDLCTRLIQEFPVLQVNEARYESHLRSLARWI
AEDDYELVIPENLPNVILAPLTVIIHIVQYFRHLNVVCTDDYGDSHTLTVQSVQHGGFQGLCSGFLTAAALTSSANKAELIAN
ASTAIRLAFCIAAYVDLGETTSLSPDGTRCLIVERRTELGETEANCEIDTIIGSFPQAYISVELNERSLTITVPASDVAPLRK
ALSDKGARTTLIPIRGRYHTENHSRSRQELLAFCETETSLQFSRQDKPLAPLRDNASGRIVTGEEPLHEVCLRCLLTERANWH
AVMSQSESNLLTCKLESPSFLELGISGSLARGLGGVTNLTSTPHSSVNTRTEDPQIFDYPEHSIAIIGAAGRFAGADSLDQLW
EIIRSGRSEEGPAPDSRSTTTNNGSLANYMTTVSKFDHEFFGISPREAQYMDPQQRIALQVAYQTVHSSNYFAKSQQPPDANI
GCYVGVAGSDYEHNVASHKPTAFSYTGTSRAFVAGRISHHFAWEGPAMTVDTACSSSSVAIHQACKDINLGECKMALAGGVNV
ISSPVINDFLHAARFLDPTGPCRSFQEGAKGYCRGEGCGFVLLKKLSAAMADGDEIMGVIAGSGSNQCDPASSITVPSCAAQM
KLQKKVLSQAGMSPTTVSYVEAHGTGTAKGDPIECQSIRKIFGRSSDDPKLAFGTVKANIGHTEAASGIAALLKVLLMLQHKA
IPPQPKFSGLNKNIAPLCLDNIEIPVQERVWDAKFRAACVNNYGASGSNAVLLVCESPNRPVQHGHGYTAKRQPLLISSHSTR
ALHEYCKGLETFLANSQLAASDLAFGLSRRQNLALSHKAAFKFETVGELREQLSRYILEPHIPKQRQRPVVLVFAGQSRDTVR
FSKAAYEGSGLLRYHLNQCDELLRSLGRKSMLPEIFSSEPIEDVVLLHSIIFSLQYSCAAAWLDSGLPVERVIGQSIGQLTAM
CIAGVVTLSDALKLIVGRASLIQNKWGPEKGSMLAVDIDRDGAKALAAAHDVEIACFNGPSRHVLVGTEAAIQDVEASTGCKV
QRVRTTNGYHSALMDCLMDDYLQLAQSITYASPRIPIETCSEGSGWSEFTPELVAQHSRGPVFVWDAIERIERDLGECVWIEG
GSFSSGVSLVKSALDAASRAGHSFHGAQLGNSNPLDHLADMTVKLWDEGIDVSFWLYHHSERHRYSPLALPGYQFDETDHWVS
YLDKGTSKSAHDLVEQPLISLTQPPAQSSARFEFVINQCHAHYVEMLQSRQVLGERLFPVAFYLELASQAVLQLSFAGIPLAT
SLQWQNIRLHAALGCRIPESLHLRFEKSSSDGWEFGIFSSEGSEKTTHCTASLSSPNVAHAMSKAAQYSCFEDVLSNPEAAAV
QGHLVYKLLGKVAEYNIDFRGIKSLAIAGLEAAAVVEVPLAARNRPRPLGCHPAHIDQFFLVAEIHALALESCGPSEAYLCRE
VGMVSSYLHTVVTQGSWKVYTRCTRKCDSELVCDTFIFDINESLVMTINNAIYVKVPLSTLRSIFDRAKGIITPEPTDEDKCG
DNSTDEGPDLYEATAELIYETTGIPPERLSSSTSITAAGVDSLAVTELESRIREVFNVEIRVDLSDPEYTFGQLCGEIQACGV
KCNDIAIQFTPSSGTSSSSYERQIPNHTFDALIPILGQYLSTASTLHAESELKSMGLDSLVSIELEANLHEAFGVRIDLMGQS
DSLTVGDLAVLLLPSVEENSASVASTGGLSGPFLGNQAVELFGHVRHEVGSCARLSGFEGFYETVYPRQTELVLTYVMEALSA
LGCNLQSLEAGQEVPAIEHMPRHIKVVSHFWKLAEESGLVARSAAGFLRTSKTVHLSSSSTLYPDMLRDFPQYKAESQLLSCT
GSRLADCLSGQADPLHLLFGSPQAVQLMQDVYKNTPMFKMGTLMLGRFLTQILPHAGQSRVPITLLEIGAGTGGTTEYILEQL
TACGIPFQYTFTDISPALVARARKTFAKYPNIEYTTLDIENLPDTAHQTYDIIISSNCIHATKDLTQSCHNCYKLLNAGGIVC
LLELTRDLWWLDCVFGLLEGWWLFEDKREHVLASESFWKQTLLAAGFGHVDWSNDSSRESDAFRLIVAQKD
>SubA
MRVNTPSLLICGPMISQADAAYLPQVRSSLVHNKDLSYLREAVSELPNLWLRLVREEPSLGEIDVALFLDNLSQWVKGNSTQP
TASRDSRNTQWAVLTVLVHIVEYMEYLDNFSSRDEDGCGHLDAHAALLDHLHDGGIQGLCIGLLTALALACAPSHAEIAKYGA
VAVRLALCCGAYIDLNEAKSPAKTICVTTRWPGDDGDDKGDIDRKCDEQLQAILDTYPDAYKSVQTDVSTVTITTNEGDVLAL
LGELEKGGATSKRIDLHGRYHYGGNQAALLKLLQLSKALPMLQFPRGSRLVVPVRNNCSGSIVEDNTALHEMALRCILVENAE
WLKTISSSISANTRQAQLLVLGPVNCVPRSLLLRSPQPISLSVSGKADNIYPDQSIAIIGSSCCFPGAENPRQLWEFIRTKQT
HGVVDAAGSFDCSFFRKSPREAEYMDPQQRLGLHLAQEALESGGYFSPSSSATKNVGCYLGISSCDYEDNVNSHPPTAYSFTG
TARAFASGRISHFFGLTGPSMVIDTACSSSGVAINTACRAIQSGECTMALAGGINLISREARTQENLAAASFLSPTGECRPFD
SKANGYRRGEGGGLVLLKKLSSAVADGDVVLGVIAATAVNQSEGNKSITLPSSESQTSLYQRVLESANLKPRHISYVEAHGTG
TQKGDPIECQSIRTVFGGTVRPACRQLHVGSIKSNIGHSEAASGIAALLKVLQMLHHRVIPPQANFEELNPAISPLHDDNIEI
SRHTKPWEERFRAALVNNYGASGTNAAMLVCQPPSIQHRLPLFPNRPCHYPILLTSHSNESLQLYCRNILRFIENQNNVVSDE
EVLANTAFHLAQRQDHSLSFRLTFSVSSIEELKLKLQQQSTSQSYKDGPIQKHSAQPVVVVLAGQTGRRVRLSHEIYASSELL
QRHLGRCDRALQTMGFASLFPGIFDTEPLEDLVQAHCMLFSLQYSVAMSWIDSGLKIDALVGHSLGQLTALCISGMLSLQDGL
KLISGRASLIQSKWGAECGAMLSVDADAETVQNLADSLPAGYKVEIACYNSSQSHVVVGTKAAITAFEKAADLRGVSLRRLAI
SHGFHSEMIDCILPDYNKLVQGLVLHPPAIAIEPCSQSGHSWANATPEIIARQSREPVYFANAISRLEKRFGSCIWLEAGWGS
AGVNMARRALTHGPTRSLSTHSFYPAALGEPDSVKALADTTINLWNAGIRVQFWLYHRSQTGSPAPLELPLHPFMKSEYLLPV
VKHSKKAQSEKDGQPIIQEKATLVSLIGKTQNAGVQTVEYSINQNSEEYSVYVRGRTVFEHLLAPVSMYIESATRAFRLLSTH
KLVSFSTSASMELKNLKLHAPFGFDLQKSLRMILRKLGEDAWEFRVESHPIHEKERGSVLQATGVITLQEVYSHLAPHRPLLR
RLYDRCEELGKDVSASVVQGDFIKKIINSVARYDDRYIGVRSITSKGFETVAHVFEPEIASQFTPTTPFNPLLLDNFLLIAEI
QANNLGGVTPDEIYVGNGFDAATAYTNAEDSEPSTKGHWVGLYSFDHQENDGILCDIFIFCAERKILSMTILGAKFQKIAISS
LKRALKTINGVPQTSGGRTPSSSITEFISGDDASPCLPIPGADKPIFIREDDFGFMTTSGHMDEENHLIPEYDVISGSSRSTS
SSPPSLESRSQAMDTEEITEGAGSALFNLLSNHLNYPKGLSPDTPLGALGLDSLVAIQLQSDIEQMFGKNSQLMDINESSTFS
TLFHTIFPQQQTDQFGFVPLHDQTGKDRLESAVPLRLGYSHIKHAAPSFNDSLDRSNTLFIRQVPHAMDALKQNISSTIKAAG
FHDFFSDVHPRQRSLVLAYIVHAFRELGCDIRSLEVGDELPSVQFKPKYQNVMNRLFDILGSEGVINVLNKRYLGGLASFPER
SAEDMHKAIMNDYPSYHPDHKLLHTTGARLADCISGKVDPLQILFQNATSIKLLEDVYVKSPMFGTGNLLLGEFMNCLFSYNK
TPDRLNHIRILEIGAGTGATTQLVVDRLLACNVDFTYTFTDVSAALVASAREKLTSRYGQHQRFDMEFETLNIEKEPPASFAQ
SYDLVISANCIHATRDLRKSCSNIEKLLRKDGGVLCLLELTRPLEWLDCVFGLLDGWWRFDDHRTYALAGEQDWKTILLQSGF
GHVDWTDDGSREAQQLRLITAWR
>DpmaA
MRVNTPSLLICGPMISQADAAYLPQVRSNLVHNKNLSYLREAVSELPNLWLRLVREEPSLGEIDVALFLDNLSQWVKGNSTQP
TASRDSRNTQWAVLTVLVQIVEYMEYLDNFSSRDEDGCGHLDAHAALLDHLHEGGIQGLCIGLLTALALACAPSHTEIAKYGA
VAVRLALCCGAYIDLNEAKSPAKTICVTTRWPGDDGDDKGDIDRKCDEQLQAILDKYPDAYKSVQTDVSTATITSNEGNVLAL
LTELEKDGAISKRIDLHGRYHYGGNQAALDKLLQLSSALPMLQFPRRSRLVVPVRNNCNGNIVEDNTALHEMALRCILVENAE
WFKTISSSISANTRQAQLLVLGPVNCVPRSLLLRSPQPISLSVSGKADNIYPDQSIAIIGSSCCFPGAENPRQLWEFIRTKQT
RGVVDAAGSFDCSFFRKSPREAEYMDPQQRLGLHLAQEALESGGYFSPSSSATKNVGCYLGISSCDYEDNVNSHPPTAYSFTG
TARAFASGRISHFFGLTGPSMVIDTACSSSGVAINTACRAIQSGECTMALAGGINLISREARTQENLAAASFLSPTGQCRPFD
SKANGYRRGEGGGLVLLKKLSSAVADGDVVLGVIAATAVNQSEGNKSITLPSSESQTSLYRRVLESANMKPRHISYVEAHGTG
TQKGDPIECQSIRTVFGGTLRPACRQLHVGSIKSNIGHSEAASGIAALLKVLQMLHHRVIPPQANFEELNPAISPLHDDNIEI
SRQTKPWEERFRAALVNNYGASGTNAAMLVCQPPSIQHSLPLFPNRPCHYPILLTSHSNESLQLYCRNILRFIENQNNVDSDE
EVLANTAFHLAQRQDHSLSFRLTFSVSSIEELKSKLQQQSTSQSYKDGPIQKHSGQPVVVVLAGQTGRRVRLSHEIYASSELL
QRHLGRCDRALQTMGFTSLFPGIFDTEPVEDLVQAHCMLFSLQYSVAMSWVDSGLKIDALVGHSLGQLTALCISGMLSLQDGL
KLISGRASLIQSKWGAECGAMLSVDADAETVQNLADSLPAGYKVEIACYNSSQSHVVVGTKAAITAFEKAADLRGVSLRRLAI
SHGFHSEMIDGILPDYNKLVQGLVLHPPAIAIEPCSQSGHSWANATPEIIARQSREPVYFANAISRLEKRFGSCIWLEAGWGS

AGVNMARRALTHGPTRSLSTHSFYPAALGEPDSVKALADTTINLWNAGIRVQFWLYHRSQTGSPAPLELPLHPFMKSEYLLPV
VKHSKKAQNEKVGQPVIQEKATLVSLIGKTQNAGVQTVEYSINQNSEEYSVYVRGRTVFEHFLAPVSMYIESATRAFRLLSTH
KLVSFSTSASMELKNLKLHAPFGFDLQKSLRMILRKLGEDAWEFRVESHPIHEKERGSILQATGVITLQEVYSHLAPHRPVLR
RLYDRCEELGKDVSASVVQGDFIKKIINSVARYDDRYIGVRSITSKGFETVAHVFEPEIASQFNPTSPFNPLLLDNFLLIAEI
QANNLGGVTPDEIYVGNGFDAATAYTNAEDSEPSTKGHWVGLYSFDHQENDGILCDIFIFCAERKILSMTILGAKFQKIAISS
LKRALKTINGVPQTSGGRTPSSSITEFISGDDASPCPPIPGADKPIFIREDDFGSMTTSGHMDEENHLIPEYDVISGSSRSTS
SSPPSLESRSQAMETEEITEGAGSALFNLLSNHLNYPKGLSPDTPLGALGLDSLVAIQLQSDIEQMFGKNSQLMDINESSTFS
TLFHTIFPQQQTDQFGFVPLHDQTGKDRLESAVPLRLGYSHIKHAAPSFNDSLDRSNTLFIRQVPHAMDALKQNISSTIKAAG
FHDFFSDVHPRQRSLVLAYIVQAFRELGCDIRSLRVGDELPSVQFKPKYQNLMNRLFDILGSEGVINVLNKRYLGGLASFPER
SAEDMHKAILNDYPSYHPDHKLLHTTGARLADCISGKVDPLQILFQNAASIKLLEDVYVKSPMFGTGNLLLGEFMNCLFSYNK
TPDRLNHIRILEIGAGTGATTQLVVDRLLACNVDFTYTFTDVSAALVASAREKLTTRYGQHQRFDMEFETLNIEKEPPASFAQ
SYDLVISANCIHATRDLRKSCSNIEKLLRKDGGMLCLLELTRPLEWLDCVFGLLDGWWRFDDHRTYALAGEQDWKTILLQSGF
DHIDWTDDGSREAQQLRLITAWR
>EsdpA
MPLPLPSAIVCGPQTNLPSQRNLDWLRSYLTQRPDVEHLLQAVLELPDLLSALQEHDHDLRLIPASSLKDLREWCLDHSIILQ
IPETLPNVLLAPLTVLIHIIQYLEYCDHLSSEDVHARVRQSIRNGGFQGLCTGSLSAAALACSTTRPEIGQNAAVALKLAMCV
GAYVDLGLALEADSPMTCFIARWRHGSQREDVDQILNNFPQAYISVNMDECSATITASKASIPALRDSLRSKSIQFAKVQVNG
RYHTASNTKFLENLLKFCRYQSNLRFPPTDYQNIPWRNNTTGKVVGRADQRHEICLRSILTEPASWYLTMSETVKTIVSATPD
DCLIPILELGLISCVPSSLSSLPSVHVSRVVVPDEAQNIPELCDYNYTDECVAIVGAACKYPGAESLDELWRVISTAQTMNGQ
APPQRYDPTDLRQGPSQSMDLSGNFISGVDQFDYSFFGISPREAMYMDPQQRIALQVAYRAVESSGYFGYGAQTTDFGCYLGV
GGSDYEHNVNAHSPTAFSFIGTSRAFISGRISHFFRWTGPSMTIDTACSSSAVAIHQACRDILSGDCEMALAGGINVMSNSTT
HQNLATANFLNATGTPCRSFDSGGNGYCRGEGCGLVVLKKLTAAVADGDHILGVIPATATNQSDGSSSITVPVSKQQISLYRR
ALSRAKMIPEDISYIEAHGTGTPRGDPIEWRSIHEVFGQARESSLNIGSVKGNIGHTEAASGVAGVLKVLLMIKYKQLPPQAH
FTSLNHDIPSAQQKHVTVNKRSLQWNRRLRAACVNNYGASGNNTVIIVCEPPKAETTKTLIRDERGKQAGTSHPFWISGHSLG
SLQRNIAVISKFVDMCHPTLDDVAFNVARMQNRSLRHRVVFGASSLEELQHRLKDPSHLSLDTHTTEDGPKPVVMVFSGQSGM
TVHLHKAVYDASCLIRKHVDQCDTILRSIGLPSLFPGIFSQNPISDIVQLHCAIFTIQYACAAAWLDSGLSVQRVLGHSFGQL
TAMCVAGIITPSDGLKLVSGRAKLIESKWGSEKGAMLSIKADRATAIALAQSEAKLGVEIACFNGPTSHVLVGSNAAIETVAS
KVSKSTVRKLNTSHGFHSRYIDSFLDEYLELAQSISYSTPTIPIETCSEISSWESFTPALVAEHSRKPVYFVDAVRRIKENLG
SCVWLEAGSGSSGVTLAKSALNGSDSDSFHGLQLGTADALESLTETTLRLWKDRVSVQFWKHHRGENSLFKPLSIPGYQFDET
SHWLPRAERVRSEPLSKKLPPMLSLENFSSPERHLSVFTIDQLSPEIAEILQARKVLDELLWPLSLYIELVSRAAALLTPALP
REFQRLRVENFEIKSPLGSRVDARLELRLKLTEQRTWEWSLEGQTSAQTLPYATGRVVLEDRRRSGTEIQRSYLSILESRHCR
GLLDDDTVFSASGSIAYKLLEKVAEYDLPYQAIASIKMNEQEALAQVSVPQAAGEWVKRKSVHPVLLDQFTLVAELHALSMIK
CKRSEVFTCSEVRETVVYEELSPVSTASWTVYTCQSSLHGRVATYDIYVFDPASKTIIFSVLGAKFVKISSHILQEIVHRANS
TPDLSENIFQIKAASPAMQTIQAADPLPTLATTLHSLPHVWSVAAQLVHELTGYAVERITAETMLCNVGMDSLATTELEHKIR
EILEVDINVQSFGKNVTFGSLVDIVSSQGSSRQAENIGSSVCSTPFTSSAGRSSPISEVDSCRILSDSMIKLCKIVEEYLGST
ESVQPGMQLRSLGLDSLVTMELESELYKTFGQQLSLMQLGEEVTINELHDLMQSHPATVADERTESKQSIAREASQSPHFVDE
AADRFAQAQDKIGEYAEAAQFLGFFDRVYPQQMSLVLAYVTEAFAALGCDLSEISPGSLIPCIPHIAKHDNVVAYYHNLLHQV
GFIIPSDNGFIRTSEPCKRVDPGPLHLDIVHSFPYHGSEHKLLRRTGAQLADCLTGKADALRLLFSDKTTGELLQDVYTNAPM
FKMGTLILGHFLPQALESFAATCKEPVRILELGAGTGGTTRYILDQLVTRGIPIRYTFTDISSTLVSRARDTFRAYDCVEYMT
LNVEQINPEVAGSYDVILSSNCIHATKDLRHSCQGLYDLLRPKGMLCLLELTRDVPWLDLTFGLLDGWWRFDDGREHVLASEQ
MWKRLLQEAGFTHVDWSNDESRESDVFRLIMALKK
>DpfgA
MATDSPSLLICGSVISDPDHAYLSRIRSSIIHNPHLAELQDAVIELPELWSLLVEREKSLQRVDAARVLRNLVEWIKCGNSSL
PLEGRTSRNTQLAVLTVLAHFSEYMIYLNSHDMSEEDGRGNLDAHTSVLEGVRDGGIQGLCVGLLSAIALACSPTITDVAKYG
TVAVRLALCVGALVDLDETELSEPTACIFARWPQSEDDDREELLKAVLENYPSSYVGVRLDVCSVNITAPKGVAMSLMRSLEE
TGAVAKQINLQGRYHHPGHEAMFQKLTDLCASLQMLQFPHHSHPLVPLRWNDSGEVVTDQTPLHEVALQCILVKRADWYTTIT
KSVTDMAQRTVASSADSKARVLALGPVDCIPRSILFITPLQVVRPMANAAFYHGYPDDSIAIIGVSCRFPGSETLPQFWEEIR
AKRVNSCLEAAGSLDCAFFRKPPREAEHMDPQHRLGLHLAYEALQSGGYFSPSSSVTDNVGCYIGMSSCDYEENVNSHPPTAY
SFTGTARAFSSGRISHFFGFTGPSMVIDTACSSSGVAIHTACKAIQSGECSMALAGGINLMVPEARSHQNLAAASFLSPTGQC
RPFDARADGYRRSEGGGFVLLKRLSAAVADNDCILGVLAASAVNNSKGSRSITLPSIESQSHLYRRVLQAAGLHPHQVSYVEA
HGTGTQKGDPIEWQSIQNVFGGRDRSGLPPLRLGSVKGNIGHCEAASGVAALVKVILMLQNRQIPPQANFSVLNPALPSLEEA
NMDIPVCLEPWEAPFRAAMVNNYGASGTNAAMLVCQPPLASPERLMSTGQPHQCPILIASHSESSIRQYCRTLMSFVETQRCV
LGDSLLPSIAFHLGQRQNESCRHRVAFSATSADELKVRLHSQARNNHDESKASKPQGRPKPVVLVFAGQTGRQALLSREAYLS
SSLLQHHLDRCDRILQTMGLHSLFPRIFETEPVDDLVDLHCMHFSLQYSVAASWIDTGLEIKAMVGHSLGQLTALCVSGVLSL
RDALKMISGRASLIQNKWGSERGCMLSVEADAPTVETIAQSMPGAGKIEIACYNAALHQVIVGTEAAIAAFEEVARSRNVSVK
RLLVSRGFHSEMMDCIVPEYQQLIQQLTLHPSVIPFEPCCKLGDNWDNITPELIARQSREPVYFSDAIRRVEKRLGPCIWLEA
GSGSAGVTMARRALTNPGTPSFPSHSFHSILLQGQNPIKSLADTTINLWNEGIRVQFWLYHASERRRFMPLELPPSPFEKSEH
WLPVLQKHKDSELANQNQDQKQEAPELVSLAGPTDGETVEFFINQHSNDYSTFVRGRTVFGQVLAPSSVYIEAVTRAFTLLPM
YLSTPSSSPPSVEVKQVRMHAPFGLDLQKRLRLTLRKETMSSWRFVVESHPIDDGDNKARKIQASGTINWQGQGCAYLEPSRP
LLRRLYDRCDELRDDRSASTVQGLFVKNILARVATYDNRYLGIQSITSKGLEAVADVAMPTIMSQACAGTVLSPPIFDNFLLI
AELHASSLEDLAEDVYICNGFDAVIPHAHPGDMVSKCEGPWMVLSYLNRENDKTVSCDIFVTSADRDILLLEIIGASLKRIPI
RSLQKALESINGIQQIQGSTARGTASTVVIDSDSDLPDSEANSPRVGSDLHADFPDLHPTYVPRISRVTSSDYPMDSSSFSSA
QPPSSASSVLSDHDQESTALLSLLSEHLNCSQGIPPDTRLGEIGLDSLVAIQLKSDVEKAFGKRLSLDTIDENLTFSDLYRMV
LNHDLPNDRGSTVLSDKAPKSKSDSSLHGQSYHVTPIRETTVSFQDSTLFTTQARLEFAQIKQETSSFAQMTGFAGFYTDVHQ
KQTSLVLAYILEAFSTLGCDLSALQAGDPLPPLRYTSKYQKLVSRFHKILEGAGLISVCEGQSVRFRTAEPLPQFGSSADTYR
ELLNECPKYRPDHQLLNVTGSRLSDCLSGRADPLQLLFRDAAAVKLLEDVYVSSPMFATGNKMLGEFLHRVLSRLGSTKRLRV
LEVGAGTGATTRNAMDQLLASNVDFTYTFTDVSIALVTSAKKKFGALYNSQRRQSNMEFTVLDIEKSPPANMLESYDLIISSN
CIHATRNLGQACANIEKLLRRDGGMLCLLELTRPLSWLDCVFGLLDGWWRFDDDRTYALADEHKWKSTLLDAGFIHVDWTDDG
YRESEQFRLITAWR

```
>DpasA
MPAESTSMLVCGSLIASHNVGSLSHLRSSLVHDPSFAGLRQQLTELPDVWSLLVDREPSLAAVDAAPLFHSLSSWLQGNSSSE
ALSLPEGAPKNILYAILTVLTHILEYATFLDRSNPTTAGDDDAHSRRLEDFQDGGVQGLCIGLLSGIAIACSKSRVELGKNAA
IAVRLAICAGACVDLAELQSAEPTVCLSARWSRHEESQTNNDCVVAATLKYYPGAYISVRSDVCSATITTNKGSVPALIKALE
EKGAVAKRINLSGRYHHSMYTPMFEKLLDICGSQPIFQFPQTARPLVPLRRSDSGELVAQDETPLHEIALRCILVETADWHKT
MVKTLETMAAKAASKSAGAESLKLQKFVLGPMDCAPKPAFAPLPIHTIRPAAVPESSYSYPDDAIAVIGLSCRFPNAETPAKF
WEMLKSKQTSTLLGPVDSFDCGLFRKSPREAEFLDPQQRLGLHLAYEALESGGYFQPSANSTDNVGCYVGVSSCDYEANVNSH
DPTAFSYTGTARAFVGGRISHFFGLTGPSLAVDTACSSSGVAIHTACRAIRAGECAMALAGGINLMTEEGRAHANLGAASFLS
STGECRPFDAAANGYRRGEGGGFVLLKRLSAAVADNDKILGVVAASAVNNSKGNKSITLPASGSQSDLYEQVLQAAGMQPSQI
SYVEAHGTGTTKGDPIECESIRKVLGKSQRPNAPPLIFGSVKGNFGHSEAASGVSAFIKTILMLQRGQIPPQANFTVLNPAIP
CVEEANMEVSTRMQSWETPFRAALVNNYGASGTNAAMVVCQPPPQRVAKQQVIEAQSTRTHKYPVIISANSPTSIRKYCESVL
ELVDTKQAALGESIVPAIACKLARSQNHAHAYRRVFAAGSIEELKAGLRGDGQGRSAAAIFQMPPGSVAKKPVVLVFAGQTGR
EVRLSEEAYLGCALLRRRLDACDRALQSLGLGDLIPRIFRAEPIDDLAYLHCMHFSVQYAVAMSWIDAGLQVSALVGHSLGQL
TSLCISGVLSLRDALKLVAGRARLIQTKWGPESGCMLSVDADAATVEALIQSMPGDDRVEIACYNSSVHHILAGTETAIAAFA
EIAHAKGVSFQRLEVTHGFHSHLVNSILPEYLELIEGLTLRKAKIPIEACSSSQQCWSKVTPQMIANQSRQSVYWSQAIARVE
ERLGPNCVWLEAGSRAVGVTMARRALAARPATIPESNSSFHSARLYGADSLDHLTQTALDLWREGVQVQSWMFHGAQAHSYAP
LELPSYCFENSHLWLPLIENSKGSDGINTAAARPVQFVSLSELSERGSEQVAKFEINQDNEEYSLFVQGRTVFGQTLAPSSVW
MEAASRALDLLPDQSADRTPAVVHQLRLHAPFGLDYQRKLILVLRRSNTSSPAWEFTVESQLLKDSSNSSDLHASGTVGRPAR
QTDTRQYQSLLRHLRERCQTLRQDPDASVVNGAFISKMMAQVADYDKSYMGIRSIACKDFEAVGEVDIPAIAVEKCATTAFIP
PLFDNLLIVGELHASSLEGLVRDNFLERESAKDLVSDMIVFRPDQKAPVLSILGARFTQISTRSLRRALESVNGAPAETSNEF
SAPTTTRFGLPSGINSGSQAYSNPGMIESDEGVRPRLNNPIRSHSASDLILDNCSDTSALSSTTSPSSVGIATPEDEENVRIL
TNLLSDHLNCSQGIPPDTPLVMLGLDSLVMMQLKSDIKKAFGSHMNVSKIDENCTLSDLCSMLFPNEPTTQLLSSTTITEKKA
VLSQSASKYEENPMPLMRALGTTSHTRSAFIERAAQEFATLKQSTSAVTRETQFANFFAEVYPDQQRLVTTYILEAFSKLGCD
LRNMQAGEILPPIAYLPKYEKLMSRFYAILEAAGIISAYNCQKLRFKTINNGGGDKASSVDLYRDILAKHPYYHPDHKLLAVT
GPYLAECLSGQDDGLQLIFQEAESRKLLEDVYRSSPMFATGNALLGHFMTQLLEQQATFADAADDVLRILEIGGGTGGTAYLM
MDLLLAQPNVKFHYTFTDISAALVASTRKQFEARYGRSCLEKHMEFTVLDAERPPPTERVGVYHMVVSSNCIHATRDLRQSCG
LIERLLRPDGGVLCLLELTRPLPWLDCVFGLLDGWWRFDDGRSYALQDEGHWKKALLESGFGRVDWSDDGTRESQQFRLITAW
R
>DpchA
MSAETPSLLVCGPLISDPDATHLARIRSALVHTPQLAELRQAVTELPGVWSLLAGGEPSLERVHAAPLLQAFAEWINKGNSSG
LLAAGQSSRNTRLAVLTVLSHLAEYVTFLRGLDAGAEEDLAGELDEHTKTLGGLRDGGVQGLCVGMLSASALACARNTSEVAE
LGAVAVRLAMCSAAFVDLDQIQSSDPTVCVSARLPRNAGEGDEQDRQPFQEALESYPEVRIQHSQSSQSWVQKEKAWLTCALR
RQAYIGVRMDVAGTTITATKSITGPLTRHLEGQGAMTKEIDLKGRFHYPGHEDALQKLVRLCESTPMLQFPQNRYPLVPLRQT
ITGDVVTDETPLHETVLRCILTETADWHTTMTRAVSALAGSPETKSSPAGNRTRLLVQLGIAECIPRSVLAGIPSIRVLQPTT
GKPARPGHYPDDAIAVIGMSCRFPDAETPEHFWEIIQSGAKAGSVFPDVGSFDCGLFRKSPREAEYMDPQHRLALHLAYEALE
AAGHFSPSSSSTDDVGCYVGMSSCDYEDHVNARPPTAFSFTGTARAFASGRISHFFGLTGPSVVVDTACSSSGVAIHTACRAV
LSGECAVALAGGVNLMTDEGQAHQNLAGASFLSPTGQCRPFDAAADGYRRGQGGGFVLLKRLSAAVADNDRVLGVIAASAVNN
SKGNRSITLPSSESQSRLYRQVLGSANVHPSHVSYVEAHGTGTQKGDPVECQSIRSVFGGSSRAGSSSPIRLGSVKGNLGHGE
AASGIASLVKVLLMLQHGVITPQANFSMLNPAIPPLEGDNMEIVVSPASWRGPFRTALVNNYGASGANAAMVVCQAPSTHLSQ
IASTRTAAMTTTHRYPFVITANTASSLHRSCLALLRFIETLPAGLNNDSLPSLAFHLAQRQNHALVHRIVFSARSVAELKAHL
LAQVEGGNIPSDTSSQAQKTGTKPVVLLFSGQTGRRAHLNRDAYTSFHLLRHHLDRCDRTLQTLGLRSLFPRIFDADPLEDLV
DLHCMQFALQYSVAASWTDSGLEIKALVGHSLGQLTALCVSGALSLADSLRMVSGRALLIQTAWKQERGCMLSVDADAATVET
LAQSVSAEDKVEIACYNAALHQVVAGTEAAVAAFENAAGSSGISTKRLAVTHAFHSKLLDDILPEYHRLLRSLEFRPAKIPIE
PCSEFGGGWDSVTPDIVGRQSREPVYFAAAMARVEQRLGSCVWLEVGSGSAAITMARRALESQKPSSQAMVSHSFYAAQLHSP
EPVSSVADSTVGLWNEGVRVQFWLYHASQRQSFVPMDLPSYQFDKTQYWLPFIERNKGSDSNDQSASSQAAPDLVTLVGSLGS
AEPQAYEFSINQESDEYALFVKGRTVFGHFLAPGSVYAESAARAFALLPTDNTPQPPASVELGQMKLHAPFGLDLQRRLRLVL
RQQTASSWEFVVESCPLHDDKEISPKPQASGTVRLQGQGRSPFGTSQSILRRLFDRCGELREDRGASVVQGAYVKKIMSRVAS
YDDRYFGIRFVASRGLEAVGDVDALPIVSECRAGTALSPPVFDNFLLVAEMHAGSLGDLADDHLYICGGFEAIVPGDQTSQTD
GPWTVLSTLERENDKTLVSDILVFHAGSKKLALSILGARLTQIPARSLQKTLDDMNAARPAKVSNSEDVLTPPLPAVESPPTD
LSNGQIHCDLRSALSPLIRSTTSLSQLTSSDDTKDSLGLSSRPSITPASSATSENDQDTTALYNLLAEHLDCSNGIPPDMPLG
NIGLDSLISIQLQSDLEKLFGKSPALKLIDENTTFLELCGMVLQQDLSSQLKSRLSPVDSANARTTLNEPLQAYGGYGHAMSV
VAPAPVSPQDTPPFLPLAVEAFKRVKKDTGAFAQKTGFAGFYPDVQQKQTSLVLSYILEAFATLGCDLGTLQLKDRLPGIPHP
AKYQRLMGRLHDILEEAGIISPPDAQLFRYRTDAPLPPPTPSEDLYRQILDECPLYRPDHQLLGVTASRLADCISGRADPLQL
LFQDQASLKLLEDVYVSSPMFSTGNSMLGEMLRGLFSQARFQREGGSEKLRILEIGAGTGATTRRVLDQLIQSGVDFSYTFTD
ISLALVNGSKRKFTASYGRQRVDSDMEFTVLDIERPPPASMLQAFHLVISSNCIHATRDLRTSCGNIEKLVRRGDGMLCLLEL
TRPLAWLDCVFGLLDGWWRFEDGRKYALAHEDEWRARLESAGFQNVDWTGDGSRESEHFRLITAWH
>DpmpA
MSVELPSLVVCGPQLEEIPDATYLARLRSSLLHDPYLRSLKQEALELHEMWPLLSATEPSLARFDAAPLLHSFAEWIRTGDSH
VLRLAGGTLRNTQLALLTVLAHLLEYTTYLQHRHHHDHAQSEHAVLTAVHDGGVQGLSIGVLSAIAISCSQSRMHLARYGAVA
LRLAVCAGAWKDLDEMHAAEPPVCLEARWEGGGARAFKAVLDSYPQAYAHVREDASNATVIVPESSAAAMARKLEEDGIEARQ
VGLQGLSHRPDHFAACQKLFNLCSSLPMLRFPEHCHPLVPLTRNGNAEAVDDGASLHEMALRCILLEKPDSAAITAQCVAAIS
RQAGEPRVLLLGRVECIPRSVPARLIRPMAAGHSLYVYPDESIAIVGASCRFAGSETPAGFWDTLRERRSTLGKAPVWRGYGS
EEEPFWGNFLASAGAFDHAFFRKSPREAAYMDPQHRLALHLAYEALESGGYFNPAAGTQTYDVGCYVGVYSSDYEDNVNARPP
TPFSFTGTARAFASGRISQFFGWTGPSLIVDTACSSSGVAIHTACKAIQSGECTMALAGGVNLMTSPKSHQNLAAASLMSRTG
QCKPFDASADGYCRGEGGGFVLLKRLSSAVADNDRVLGVLAASAINNSKGSLTITAPSLESQAALYQSVLRKAGMQPDQVSYV
EAHGTGTQKGDPVECHSLRRVFGRSSRNSPPLRFGSVKGNIGHSEGASGVASLVKVLLMLQHGLIAPQANFSVLNPAAPNLEE
ANMEIPLYLQPWDAAFRAACVNNYGAAGNNTAMIVCQPPATQPVSRPSSVQKRHQYPFMLTAHSDASLRQHCRILLQFVEDQQ
AWAGDDLLASLAFHLAQRQSHQLGYRTAFSARSIDDLKARLGEQNTQTRGNCNPVVLVFAGQTGHRPRLSEEAYHSSFLLQHH
LDRCDRTLQTLGLRGLFPQVFGTQAVDDLVDLHCMLFSIQYATAAAWIDAGLDVRKLVGHSLGQLTALCVARVLTLRDALKMI
```

SGRAALIQSKWGPEQGCMLSVDSDAVTVRALIDSMASEEKVEIACYNAPSSHVVVGKAGATAAFESAALSAGVRTKRLAITHA
FHSPMVDSIMEDYESLLRELQFHSPTIPIEPCEQSGGSWENLTPERVARQSRAPVYFGAAVSRVERELGSCVWLEAGAGPAGV
TMARRAASSSSHAFLSARLGSPDAMDSLADTTLSLWREGVRVQFWPFHPWQRHCFRLLELPPYQFETTHHWLPFASAPESAAQ
QPTTANDVAPQLVSVVRSSGGADPEAAEFTINQHSEEYALFVGGRTVLGHALSPPSVYLESAARALGLVSAAAGGPAALPPHF
EQVQLHAPLGIDPRRRIRLRLQKHNTSAWEFVFDSQAPALDGGQTFQLQASGIIKSQEQDRAVAGPYRPLLRRLIDHERCRVL
LEDSGASVVQGAFVKNILGRVASYEDSYFGIRSITSKGHEAVGVVDVPEIAHQRCAETRVNPPLLDNFMLVAEMHAGNLDACG
SDQMYVCNGFDALVPHSNDGSLRGPFTVYSKLERESDRVFVGDVFVLTGGQKTLSLAILGARFSKVPVRSLQRALEAANGSPN
VRTAEALDHSAVAIEARDSALTSPPIRPHAPPSSDAVSTISLNEVKTTTERVISETTGVPRERINDATLLGDLGVDSLMATEL
QVRFSDVLHVDLAIGTLCEHGMTAGRLCQEIHSRLSGVPQLSPHDTDRSSDLSAGQPPSTPKASTQEQEHFIVELSKLLAEHL
NCSPDIPPETPLALMGLDSLLAIQLASDMESRFGKKSSPMNIDENTTFSDLCRVLSGADLPGFPRTSDNRRSEEGSVGHVGPE
KSEASFFREREDVIKLEFDRAKQRYGVFSEQAGLAGFYARVYPRQMALVLAYIVEAFRTLGCDVATLRAGERLPPIPHEPRYE
KLVRRYLQLLEDAGLITSSGEHPPAHLRTAKALEHAESSRLHRAILADFPAYRPDHRLLQLTGPRLADCVSGKVDPLQLLFHG
PASRQLLEAFYVSSPMFATATRMMSEFVGQLLRKHGGCSERLRVLEVGAGTGATTQLLDQLVASGAAFTYTFTDVSSSLVAA
ARRRLEARYAAAGHEMHFAVLDIERPPPQRLLHSQDLVVASNVLHATRSLSDTCSNVQRLLRPGCGVLCLLELTRPLPWLDCV
FGLLDGWWRFADSRTYPLVDEWRWKACLLNAGFRHVDWTDDESREADLFRWILALA

>MPAS

MALPSLIAFGALAPWPASDRLDQLRNALQHHNSLKPITKAIQELPLLWKALSNQDQSLHSIAGEAAADQLAQWISGAGTAQLV
DDKGNVTRMPLTTIAQIAQYVSYLCQYEEPLRHESIIKSAAIGGGIQGFCIGLLSALAVASGKTEDDVGNFAAMSVRLAFCVG
AYVDLDRHRNGGDSKASTIAVRWKTPTTLEDIQRLLSRHPDTYIAVVRDIRDVTITVPASVMEHLIEDLSQIGASLRDTGVSG
RYHVAIHEGIPQKILETCQAQFSPTINGQPLVRSNTDAHLFSGEDTALLALECILGERADWYSTISTAASALNQISANPFILS
IGTDPVPQSVARSFPVVKATMIADRVNGIVEPEIPALAPDPFGSVSQGYPKDAIAIIGMGCRFPGADSIDEYWNLLTEGKSML
SEIPEARFGRGRPARSNSSLRFWGNFLRDIEAFDHGFFKKSPREAVSMDPQQRVLLQVAYEALESSGYFADSSRPEDVGCYIG
ACATDYDFNVASHPPSAYSAIGTLRSFLSGKLSHYFGWSGPSLVLDTACSSSAVAIHTACTALRTGQCSQALAGGITLMTSPY
LYENFAAAHFLSPTGSSKPFSADADGYCRGEGGGLVVLKRLSDALRDNDHILGVIAGSAVNQNDNCVPITVPHTSSQGNLYER
VTEQAGVRPSEVTFVEAHGTGTPVGDPIEMESIRRVFGGLHRVAPLIVSSAKGNIGHLEGASGVAALIKALLQMEHHLAPRQA
SFKTLNPKIPALEPDNLCIPTSNLALSGERLAACINNYGAAGSNAAMIVLEPPRKSVTYHDKSKMSISSRPKIHPIQLAAASL
GGLLAYCVALDQYCQRLRFTQDTSEQPQVLSDLAYSLSTRLNQELPFTLTMTVTDLDQLQAQLRQQTVTSNNIKQRSKAPPVV
LCFGGQVSDRVALDKCLWQESTLLRSYLDICDNTLRVLGYPGLYPSIFQNEAVTDVVLLHSMIFALQYSCAQAWLESGLKVDA
LVGHSFGQLTALCVSGILSLRDGLRLVAGRASLMQKHWGPESGKMIAIETDQQTLEELQKVICESNASYNFEIACFNGPTSHV
VVSDRCSASELETKLMERAIRHRSLDVPYGFHSRFTEPLLPHLEDLASSLTFHEPKIPLETCTDMGTWTEPTSKLIAAHTREP
VFFGKAIQRLQARLGPCTWLEAGSDSSIVNMVRRALGQASATANNFVSLQLNKPNSSKLVVDATVALWDASHRAQFWNFHRLQ
RRQYDHLRLPPYAWEKSKHWLELDMSAALNSDKTNTPPPTNTAAQVELPAVLIRLKSFDSQGHHFVINPSSEEYQTIVKDLES
LGSAVCPSTLYVELASRAVRVAEEDKGNGLLSIKDLRVHSLLGVNVHQTISLDLQRLAQSWRFRITNADGSISGSNPGESFCH
AEGTVNLKVADDSLEEEFCRYERLTGHNKIISIADDPRSESLRGNVLYNMLGRVVNYPDWYRGVKSVAALDLRVVAKVTCPVG
IPEIVSKESTTQLPILESFIQIASLHANCLHECRGGEVFQFTRADHMQWAPGFDLHGYGDSAEASWDVLAYNSTNAENVVYDI
FVHDAVTGRLVLLVLGANFTDIRRPVPISAGLNTSLASEKDIPMLKNANAERAEISLNSQLPAESHSQANLTRPGKDAKTSIY
EDICGLLEKLADIPGDQVSGEATFDNLGVDSLMMIEVISELSTLFRVDLPIHELEELTDINSLVDYLHGKGCVGSLYVEDSGN
ASSLSSSHAISTGASSPPDSSGASAMTTPPETLSLVDYPGSLTTKQESRAAPAISNGTGRQPLDMGPYGIQQVFTRLRFDFEK
YAEQTGAKGFWTNVYPQQADLVCAYVVDAYRKLGCDLATLAAGQQLPSMNTLPRHKHLVAQLRNILVESGLLELRGNQVHVRT
AKTVDSTPTAIRYEQMLQRHPFGASETKLLNVTGPRLADCLTGQKEPLSLLFGDKHNRDLLADFYANSQMLKAATRLLAEFVS
STFSAAQSGDTLCILEVGAGTGGTTRYLVDVLNRCGIPYEYTFTDISQSLVTQAKRNFASLPQMRFMTFDCDRPAPQELLGKF
HIVISTNCIHATSNITTSTTNILPTLRDDGVLCLVEFTRNLYWFDLVFGLLEGWWLFSDGRQHALANEWFWDRSLRAAGFKHV
SWTDGNTEEAKTLRLICAFRGEAKEDRNLAAPNGAITKRAGVPMEEVVWKRVGTLDLSADIYFPKTPDPPGKKRPIALLIHGG
GHFLFGRKDVPMKHIRTLIERGFLPISTDYRLCPETNLFEGPMTDCCDALKWATETLPTLPLSGPTVRPDPTKVVSLGWSSGG
QLAMSLGYTAPVKGIKAPDAIFALYPPSDMESNHWHQPCYPLAAEEEPTEILDILAGVRESPIVEYAPVSEKRTMALSLTLKD
DRASIILHMCWKSQTVPILVHGLPYKKNLPDTDKTDWKYRPPASAEQVQAISPLWQIRQGNYKTPTFIVHGNGDDWLPLSMSE
RTVEELKRRGIPASLAVPEQCGHAFDLFPVGDPLGVGWTSLEQGYDFICRQLGMS

>PkbA

MAPAPSALVFGSQTTLPSVEAASRLRAALLLDPRLYRMRTSIESLPEIWPALAISDPALERVAGPAEKSLRQLCRWLSHSEFP
DATEISELPATFVTPFTVILHTVLYMHYTDENGSRGHADVLRAVRNNGGVQGFCTGFLTAVSVATSPDLEALSRQASVALRLA
VAIGAYIDLDLLDSDVSSVAVRSRAGKKGLEETLAQFPGAYISVITDELNATVTAPRASLDALSQSLASNGLSAKRFDLRGRF
HHPAHQKALEGLYNLVASNPVFQFSHSELLAPVYSNIDGQLLSSDSIIDTLLQSILVQRCDWYASISTALHKRSNGEKTSVVQ
FSLVECIPPSVLRQARLSVQRITDVPVQNSPATVPAPLNAVRARIQTSEPIAIIGMGCKFPGADTLDEYWQLLAQGTSMCRTM
PEERFKTSSLRRSPGEKLKFWGNFVNDVDAFDHRFFKKSSREAASMDPQQRLVLQVAYQTLESAEYSGLSGIKASRDVGCYLG
LCASDYTDNVASHPPNAFSSLGTLRAFLSGKISHFFGWTGPSITYDTACSSSAVAIQAACRALQTGECSMALAGGVSLYTSPN
FYQNLSAASFLSPTGPTKPFDAKGDGYCRGEGVGLVFLKPLSSALADHDNIMGVIAAAAVNQNQNSTAITVPHSESQIELYRK
VVSEAGLHPHDVSFVEAHGTGTPVGDPIEFTSIRTVFGGSNRANPLAIASVKGNIGHTEGAAACINNYGAAGSNAAMIVTEAP
TGARSEKQGTLPKCPIYVSANTVSSLKEYCKELLRSLRGRSPDCLASLAFQLANSQNRGFPHALITSVTSKAELEDQLSAVVE
NRNNSLHTVAPTERPVVLAFGGQVARSVGLSRQVYDSSAVLRTHLSNCDDILTSAGLNSIFPAIFRKEPIADVVLLHSALFSA
QYACAMTWIEAGVIPAALIGHSFGQLTALSVGRVLSLKDGLGLITERAKLMRDAWGPEHGSMVSVQADVQTVARLMKAAETKD
PKDALEIACFNGPTSHVVVGSADAADRLEAALTQESIRYKRLAVSHGFHSKFTEPLLLGLEQCAERLTFRTPKYAIETCSSGS
SWSEFNASMIVQHTRTPVYYTEALARIEAKLGACTWLEVGTGGSVAGMIRGALNVPSDHLIQAVNLAGETGTAALADATVNLW
KSSHKLQFWAFHRSERECYQPLELPPYQFEKTRHWLDWKDTMTEQATVTQSTREETSVEEFLTFVKYKDSTKQQAEFRISTEH
EKYSFFVVKGHAVLAEPLCPAPLYIDLACKAGQMVYSDTSETLIIPSVEDLEIQAPLGVGDRVIILRLQQSPFLKTAWTFCFCS
RPVMGNSAEEQLHASGTVVLRENDTKTAAEFSRFGRLVSSKRVQEMKSDPDCHILQGPVVYQLFSRVVSYADYYKGVQSVYAS
GAEVTGRIRLPPTVKDADTRRPLLVDNFIQTAGIHVNCLTDVGAKEVYVCTKVDRVQSAAAFTEDLANVDASWIVHSSYHPTS
EKEVVNDIFVFNAATGELAMFILGAHFTRVQISSLGRVLSRANTADAAPIKVAVPVQSPALRAQPKRVLLPPLTKRSITRPTL
EISEKLKKTLSRVVEVPVADIHDGGILADLGVDSLLGTEVLTEINQVFNVSIPADEFALLTDVASISKCLASYLGVHDSGSQP
EDLADADSVESDSDMPTGAVTSGITTPDDAVSRLADLLAENLEYDGTIEASNNLADLGLDSILSIELANDIKKIFNCDVDMSQ

LNMESTFADLIALVPALNIEQSLSSVPASLTTQGSDFEMAQHAFEQIRFDYDIYTKETGFYDFWKRVYPAQSRLVLAYTVEAF
AQLGCDLALMHPGDRLPKIGYLPAHEKLVQQLYNILRDGMLVATSDSGFVRSDKPVDPTSSTRLLEEINTIAPQHASEHSLLH
ITGSKLADCLTGTADPLNLLFRSKANRDLLAEVYLNGPMYAAISRLLCSFLGNAFSDRQSSGTFQILELGGGTGGTTGHVLDY
LVRSGIPFTYTFSDVSGSFVAAARKKFAGRPYMEYQVIDIEKEPADSLTGKFHAVISTNCIHATTNLEISTGNIHRMLRPDGF
VALVEFTRNMYWFDLVFGLLEGWWLFEDGRQHVIASESFWNTSMRKAGFQHVSWTDGDSFEARTLRIIAGFRAPAVNEIYTPR
LDSRDTETAVESVMYKQADGIPLFADIYYPPSVSNEPRPIALMIHGGGHIMLSRKDIRPKQTAHLHTLGFLPVSIDYRLCPET
TLTEGPMRDVSDAMAWARSTLCSLPLLCPGLTLDPSRVVVVGWSTGGHLAMTTAFTSIERGLSPPDAILAFYCPTDYEDRVWT
QPNYPENTDVDGLSLMKYNLLEGVQDRPITAYNIPLTSRSNSKAGGTPAGGWMAPDDPRSRIVLHMNWKGQCLPVLLRGLPPS
NSLSPGDAEKLISQPQPEIEEIQRVSPYAQIRRGVYRTPTFVIHGTDDDLIPYEQSVRTVQALKDMGVRAEVSVPQGKAHLFD
MFKDADGSSWEVVKRGYDFLKEEVSGK
>Preu3
MNPPSALAFGPEERIPTASNLRLLKDVLQDDPTFAGITACLKQLPDTWKALLHQDAQLQSLASERRAAVLSNSLLNDEQHEGD
MDTNQVIMPMTVLVHMVQYRQFLQQSSPSSHATVMQSVAAGGVQGFCAGLLSAFAVCSMTNEDDFDACATYAIKLAMCVGAYV
DLAMESEKGDMASAIVRWSIPDGRNRVNKAVGRYQSAYISAISDEDNVTVTASRPDLDAICTSLGSTGMSSKILAMTGSFHHP
KNFDLLQRMISLLRAPQLAPSTKFTNALLRSNSTGELLTGAKTIENILEDILCKTADWRLTMANTSKALRSGNGSRPNIHTFG
LVEFIPSFVKNEFNILTQRLAPTAKEQTGASPSKSTLQYNDNAVAVVGMACRFPGADDLDEFWELLQSGKSMHERMPADRFST
TGLRRSNDGAPFWGNFLKDIDAFDHQFFKKSSREAAAMDPQQRLLLQCAYVAMENAGYFDPSVQHKIRDTGVYLGACSSDYND
NVASHKPTAYSTLGTLRAFLTGRISHYFDWTGPSVVYDTACSSSAVAIDAACKAILAGDCQQALAGGVSLYTSPNFYQNLDAA
SFLSQTGPCKPFDANADGYCRGEGVGLVVLKKLSDAIRCGDKIVAVIASTGVNQNRNCTGITVPHGGSQADLYRRVVAKSGLN
ASQVSYVEAHGTGTPVGDPIEFTSIKSVFANPDITRDEPLTIASVKGSIGHLEGAAGVASLIKVCLMLQHSAIPPQANFTKPN
PNLGGVDMRNIVIPTSSIPWKARNKVACINNYGAAGSNGAMIVCQPSEPASKTQTRLPSQSLSYPLFISGDGTDAVEANCRAI
AKYARQLQQKRAPSVVASLAYRLATSQNQNLSYAMVTTISENGDIESTLTKASATLTQPRSKAKQSVVLCFGGQVKAFVGLDQ
QLFDSSSILQKHLRLCDSTMRDLGYPSIFPAIFQSEPLKDPVQLHGVLFAMQYSSAKAWLDCSLQVDAVVGHSFGQLTALTVA
GVLSLKDGLRLVCGRAHLIKTKWGSATGAMIAIEAPLIRVQEILSKISVAGHEAEIACYNARESHVLVGTTTAIDAVRTFVLE
SGIKHKRLPVTHGFHSTFTEALLPGLRELAKGLQFKSPIIPIETCTEYKSWEQATADMITKHTREPVYFVHAIERLSARLGPC
TWVEAGTGASTPAMIKRCLPDSCADSFIHATLESNKAFGSLADATANLWRCSQPVQFWPFHASDRGRYVPLNLPGYQFRKTKH
WLEWQDTVALPAFLEKEPSTSEPKGHELLTFSSFEDTSKSVAAFKVDPESDEFMMLVKGHAVVAQPLCPAPLYCELALRAIKH
LSPETASNAPDIRDLQIHAPLGLKTNRNIRLVVQKNSIPGHWTFTVKSSMGSDDELTHAHGLVAFGGTVEQELASYQRLIGHQ
KIQSLMTDPECDALRGSATYKAFNRVVTYSSYYKGVQAIYGRQNEACGKIELSSGEEQMAQARGILTPLLADNFIQIAGLQIN
VLGDCEDHLVFVCTETQRIIYGPGLHQQPAARYEVYSTISQNGPKEVMSDVVVFDPATKNVEFVALGCRFTRVTVPGLRNALQ
AANGDARAQERPSGSRISPSPLAPELPAKIQIQSRENLDITEKSGRGKPPRVENIQIATPKVDYLAQVKALLHKVSDVPIDTI
QKDSTLDDLGIDSLMVMEVQTEVHSEFQLTIPNKDWATLETPGKLAEYLAKTLGGSVPDSAPPGVQRVPALVISDAEQSSDES
PYDSTDDSASGYGDLDIDTAATTPGIFATRDSSPFRKAALDSPNPVNKVAQRTFSDIRPKYDVFAAEEGFAGFWRDVYPRQKR
LTLAYVVEAFAVMGCDLSDLAAGQILPKIEYLPQHVSLIKQLYVILADSTLITIENGTYRRTRVSVDTTPASDLLADILRAFP
QHAEEHRLLDVTGSRLGDCLIGRADPLRLLFMDRANKELLDSVYANGPMYKAMSRLLGSYILDTMQQWQGQKPLRILEIGGGT
GGTTKHIVKLLHQQGIDFTYCFSDLSRALVTKAKKTFSIYPQMEYMVLDIEAPPSSEYLGQFDLILSTNCIHATKNIQQTTKH
MRQLLSSEGFICLVEFTRNIFWFDLVFGLLDGWWLFEDGRPHVLADENLWDQSLRAAGYGDVQWTEGQSEESKTLRLIAAFNV
SNEDAKAANALASALAVPGRKGRTSATTIRWKQEGDLDLMADVYLPSDLDASTVSRPVALILHGGGHVLHTRKHINPRHIKML
QDLGFLPVSVDYRLCPEVNIRDGPMTDACEAVDWARNILPCLPVCSELRVDKEHVVVIGYSTGGHLALTTAFTTRVRGFKPPS
AILGFYCPTNYSADWWRSPIYPELAQQSSSETFDLLEGVNEHAIAGYTPTVNNNVAALLMSLDDPRWRFVLHANWRAQTLPML
INGLPSKSRLARSGQTVDSVINREIPDAEDVASISPYDQIVRGSYSTPTFLLHGTKDDLIPWQQSIATVDALARRGVNARVEI
IEGAEHCFDVWSDKYDGMIGRALEWLVEQCRNA
>Sor2
MAASSTRTLLMFGPGAMSLNETYFASILSFISTDSASQWALSAVRDIESHWPSLCEAIPKLQHTSGVSNAQKLAEWLRTGTLA
PGSTIASLPNAILGPLVVIAQLVEYLRHVDSLSESGLRGGEGFQVPSAPDAETVGCCLGTFSALVVSSSSSWAQFCHNASAVV
RIVFVLGALSDAQDATDASGPSVSLIAFWRGGQSLSDLKKALEKFPEAYISVLYDENRVTVTTSTRTVAALKNHLQTVGITTN
ETEFHGRFHAAQLYQTELEAFLAYCRRFPTFQLPDSSCIVVPTRVNSENVVTSQESLLEISCRAFLVSQFDWIKTFRAAVSST
LQNRASRIIEFGPERCVPPTLLRRLNSQVTHFDFEESIKRAKASLSHDQELPAGVAENDIAVIGMACKVAGADDVNEYWELLL
QGKSQHRELIPNDRFVMETPFRPFEAGDDKKKWFGNFIGDHDAFDYKFFKKSPREALHMDPQQRLMLQAAYQAVAQSGYYNAN
LNVHGPTKVGCYIGVVANDYENNISHTTPIAFSATGALRSYIAGKVSHFFGWTGPAMTVDTACSASTVALDLACKAILSGECS
AALVGGTNFFSTPMFFQNLAAGSFLSTTGQCKPFDAKADGYCRGEAVGTVFLKKLSQAIADGDQVLGVISATAINQNRNETPI
FVPNSPSLTNVFRTAIEKSGLDAKDISVVEAHGTGTPVGDPVEYDSIRQVFGGAVRAGQDALQVGSVKGLVGHTEGASGVVAL
VKILLMLQRGQIPPQASFETINPSIKYSPSDNLEITKTPLPWNQEFKAALINNYGAAGSNASVIIKQGPAQLLRRLPPVVDSE
TEALLSKVGADDAQKAPFFISGLDEKAILAYAQKLRQFIYSHSNLDIQDLAFSVNRQSNWSLGRGAVFSAGSIAELDEKLASI
ETFPVPSSQPPVILCFGGQVSTFVGLDYQLFAKSAILRRHLDQCDAACKSIGAGSIYPRIFQSDPIDDPSVLQPLLFSLQYSC
AMSWIDCGIVPAKLVGHSFGELTALCISGVVSLQDGLKLVYGRSKIIKESWGAERGAMLAVEADLEELESLLTTVNSSLQEGR
ATIACFNGPRSFTVAGSSAAIDAVQQAISNTQPVLKHKRLNVTNAFHSVLVERLKPDLEALGRQLTFALPQIPLERATRSRED
HGLSPSYVANHMREPVYFHHAVERIAKEYPEAIWLEAGSNSTITTMASKALGLPKASTFQPVNVTNNSKASSQLSDVTMNLWK
AGLRITFWPHSRAQTYEYKHIILPPYQFEKHRQWLEFKPPQALQVVVQTDSSTDARNGTTEPQPVGLYTLLDRGQDKYRFRVN
TAAGQFVDAMSDHAIGKAQTLPAMFGVDLAIEALLSIHPELGDTSRFDPQIYNVVNQEYIHDSSRALFVLFERLGQDENSWAF
ELTSKGKDGAESLHMSGQLHFQASDDARSRLEFSRLDRFITHDRCLQVLESSGRSDEVIQGQTIYSVLSSSDVNYGQRLRGLQ
RLVGRSNESAGRLVRRRSGKLFVDFALGEVFTQVGSIWANCMAQHQRNTRNKGIYMATGLEQWSKSPKVLQKFNQGLYDNDPE
IEWHVLAQHKRNTSDDSFTTDIFVFDAASGDLEEVILGIKYAPVSLDQLFSGSAIATATTPVANGYVPLTTPFVPVPTTTKQA
AVPQPQHVAKKAAPRAAPKRDIKEELWLRLRPVLADISGLEPEEIQPTDALADIGIDSLMGMEMAREVETTFNCTVEQSELLS
IVDVPGILKFLQSTLGDEDVHDSSETMSTVSSDGNVHSPPTSGSEMASPNLKVSYGSAYGSSDLPISAVLEAFGESSAKTDQF
LKTYGCAGYLDGVSQKQTRLCLVLTSAAFKQLGCDLEAAKPGEVLQPVPFVERHRRFHQYLYSMLEETRIINIDGDVITRTAI
PLPSQSADAILQDLMRRHADNGSSHQLAYNVGSRMADVLSGKADGPQLIFGDAKNRELVANFYGELPFNKLYFELMADFLTRL
ANKLKLSSSRSGTPTLKILEMGAGTGGTTKVLLPILAKLGIPLEYTFTDLSPSLVAQAKRRFKEYPFMKFAVHDIEQPPSDPE
LVGSQHVVIASNAVHATHSLRDSARNIHKFLRPDGFLMLLEMMRTLHWVDVVWGTLEGWWLFDDGRTHAIVNEQRWEKELLAS

GFKHVRWTDGKLPEVHVQRVIIALAGDGDGDVSDIPALTSPALKDEEDHGSKLDGEERKRVANAYVESTIRDFAIPSYTGPIL
STAPGAGACVLVTGATGSLGSHLVAHIAGLPSVDTIYCLNRPRPGKKGQEDQSRDPLSRLTEVLASKSIQLSEAEISKLRVIE
TDLPLPQFGLDEIQYEQLLNNVTHIVHSAFPVNGLRSLKQNEPQFTLMRNLVDLAAGVSARRPTEFKFTFQFISSLSAVGMYP
KVHGEKRVPEQQWDVDSALPNGYGEAKVICERVLLETLGQHPDRFRAMTVRLGQLSGSMKTGYWNHMEMLSFLFKSAQTLRAL
PDVDGDVSWLHLEDASASLADLLLRDAPTCHAVYHLDNPRPRDWKDVIPVLADALDIPSSHIVPFEEWLRRVRAYPGEDPWDN
PSAKAMDFFEHKFKHMSCGGVTMATDHALEHSETLRAVQPVSDELVRKYIQAWKDSGFLR
>SorB
MAENSGRGYQTPVHRDVFFSKSAPQSGNTADDIPNAASQPDTTSTMAMPSAKTLLLFGPGAMSLDQTYFSRILSFVKDDAASQ
WAVRAIEDIESGWDALSESIPKLQQTPGADHARRLAEWLRTGVITPRTTVANLPNAILGPLVIIAQLVEYLQYVESSQSANGD
GKLFQLPSTAQTETVGCCLGVFSALVVSSSSSWAKFHHNAAAVLRKVFVLGALSDAQDISDVTGSSVSLIAFWRGGQSLSDLK
KVLEICPGAYISVLYDDNRATVTTPSRIASDLKGHLSRAGFTASETEFHGRFHAGELYNNDLEALFSFCRKDPLFQLPDASSL
ILRTRVNSEKILADNDSLLEVASRAFLVEQFNWVKTFRSAVSSSLQDRTSKVIEFGPERCVPPTLLRRLNSQVTHYEFQSTGQ
RHSNPDMPSGCIDNDIAVIGMSCQVAGAQDLEQYWNILLEGRSQHKNLVPNERFAMETVFRPGQDGEDRKWYGNFIDDYDAFD
YKFFRKSPREVLHMDPQQRLILQTAYQAVAQSGYYHRPGADRRIGCYIGCVANDYENNISHTSPTAFSATGALRSYIAGKVSH
YFGWTGPGMMLDTACSASTVAIDLACRAILSGDCSAALAGGTNFYSTPMFFQNLAAGSFLSPTGQCKPFDAKADGYCRGEAIG
AVFLKKLSNAIADGDQILGVISATAINQNQNDTPIFVPNPSSLTNVFQNVVGKAGLEVNDISVVEAHGTGTPVGDPAEYDSIR
QVFGGSVRAGLKPLQLGSVKGLIGHTEGASGVVALIKMLLMMQESRIPPQASFTSMSASIKASPADNMEITKAALPWEDESKV
ALINNYGAAGSNASMVIKQAPKYPSGSEAVGHGSADLTSPTSTFRCPFYISGLDDKAIRAYATRLRQFIKNKVISRDVLGIEN
LSFNVNRQSNWSLSRGFVFGAESITELEEKLASFETFAVPSVRPVILCFGGQVSKSVGLDRGVFDKATVLRKYLDRCDSVCKS
IGAGSIYPGIFQSEPILDPAVLQPLLLSMQYSCALSWIDCGVEPAALVGHSFGELTALCVSGILSLEDALKLVHGRSKIIKES
WGPEKGSMIAVEADRNDVEKLLVASNARLGETERAGHATIACFNGPKSFTIAGSAAAIDAVQQTVSTLDIPIKHKRLDVTNAF
HSTLVEHLRPQLEALGRSLSFGNAHIPLERATEQRETGPISPAYVAEHMRNPVYFDHAVQRLASQYPEAIWLEAGSNSTITTM
TGRALGMPKGSTFQPVSVTGTTQGTRQLADVTMSLWNAGLPCSFWPHSRAQTYGYAPIMLPPYQFEKYRHWLEFKPPPKPVVI
ERLVYENGGVDQEAPAPGLYTFMGYGDKTETDCRFRINTTTKSYVDIVSGYTLGKTVQACPPIFGIDTAIQAITSVRLEVIAA
NELHPHIYNVLNHLPLVMDPTRAVFLEFERSGHAPEGWKFKLTSEADDSSKTVHLSGQLEFHRADDARSNFEFSRLERLVTHE
RCLRALESADDADEVIQGQSIYKVYSDLVSYAPKFRGLQRLVGRPSESAGRAVKRRSRDSWLDFALGETFSQVGSIWVNCLAP
GRNTADDTVYIADGIEQWMRSPSLLRKISEGSYADHQSEWQILATHKRTEGDTFITDIFVFDSASRLLDEALLGVKFSARSMS
ELFTNVVIAAPPVPFPATIAPISSAPTENQYSSMTTSPPARAQVQKRNTKTELWAKLLPVLADISGLEPEEINETDALADIGI
DSLMGMEMAREVETTFNCTLEQSELMSIFDVPGILAFLQSTLGLEGEDDASQSSDAASSSRNTPPSSNDGILATPSPKLEEED
ISRSYIDLGNEVGLPAFAVIEAFRAANEQTDAYLKKWKCAGYLDGASQKQTRLCLVLTSDAFKQLGCDLVAAKPGEVLQPVPF
VPRHHRFHEYLYKMLEETRIIDVDEGIITRTALPLPTQSSQAILDDLMSHHPDDGPSHQLTYNIGSRMADVLSGKADGPQLIF
GDAKNRELVAAFYGELPFNKLYFQLMADFLSRIAESLRLCAQNRGPLKILEMGAGTGGTTKVLVPALAKLGIPVEYTFTDLSP
SLVAQAKKKFKQYPFMKFAVHDIEQPPSDPLLIGSQNIVIASNAVHATHSLQVSTQNIRKFLRPDGFLMLLEMNSTLHWVDVV
WGTLEGWWLFEDGRTHAVVDERQWEKELLDAGYKHVEWTDGKLPEVRVQRVRIALADDVEQNVGRLPPVAKQQVDDHDLSEEE
LNEKKQVADDYVKETIRGFTIPAYSGDLTDSSEYGKAVLVTGTTGSLGSHIIAHLVSLPSVDRVYCLNRPAIGGARAKDATPR
DPLHRQLQSLESKSIALDASQLAKLKVIETDSSKAQLGLDTEEYKHLLCHVTHIIHNAFPVNGLRSLKQNEAQFTIMRNLVDL
AAEISARRKATDFKFAFQFISSLSAVGKYPSVHAGEIQVPEEHLNIDSALPNGYGGAKVICERILHETLGQYPERFRAMTVRL
GQLSGSMETGYWNHMEVLGFLFKSAQTLRSFPAVEGILTWLPLEQASATLADLLLRDAPDCHPVYHVDNPVRKPWAEIVPVLA
QALGIPEKGIVPLDDWLRRVKAFPGEDPWDNPAGKAIDFFEHKFQHMSCGGVTMATNNAVEHSPTLRGVQPVADAVVMKYFQV
WKDTGFLR
>DbaI
MLGHRDFTTLPLSRREFLLFGPLALSFDQAAFEHLRKTIVNSEEHRWALEVLGSLPQYYATIVNAFPGINGRNEVQLEDLKGA
LHSGKPLATSFPLPNTLLIPLVMVLHLTEYSRFLQEISEELESGIDLFDASRHNKETVGFCTGLLSAMAVSSAGSREDFRKYA
AVAVRLGLLVGVVVDSHDISSAQGPSKSISASWNSAQKREDARRIMDEFPQAYISVYYDEDRATITAPASEISDLHRRLRASG
IVTAEIGLNGCFHADCYLDQLDPIIQFCDSQPDFQLPDASKVVIPTRSNATGELIRDGALHQHALRSILVEPPQWFESFTAVR
DACAEDEGAIIFSFGPERCVPPSLLRVLSQKVVTVEDLDVLKRYQYSYSENDIAVVGMSCKVAGANNLEEFWDLLCTGKSQHR
EVPKERFSFETVFRDVDSKRKWFGNFIDGHDQFDHKFFKKSPRESATMDPQQRHLLQIAYQAVEQSGYFHSANPDRQIGCYMG
VCACDYENNIACHAPNAFSATGNLQGFIAGKVSHFFGWTGPGLTIDTACSSSAVAVHQACKAIITGECTAALAGGTHVMTNPL
WFQNLAGASFLSTTGQCKPFDAKADGYCRGEGIATVFLKKLSAAVADGDQILGVITATAVQQNQNCTPIFVPNVPSLSDLFRV
VVKQSRLQPSDVTVVEAHGTGTAVGDPAEYDSIRSVLGGSSREKTLALSSVKGLVGHIECTSGIVSLIKVLLMLQKRMIPPQA
SFTTINPAIKATPADKINIPTTVKTWDAEFCAALINNYGASGSNASIVVTQPPVGTVKPSAETSGLKYPFRFCGMDEQSLRRY
SKIFRQFLNRKSYSAQDLSLRNISFNVNRQSNRQLDRTLLFSVKTLEELEQKLVTFENDNDSITSLALPKSKPVVLCFGGQVS
TFVGLDRTVYERVAILRKHLHTVDAVARSIGLKSIFPRIFETTPVSDTVHLQIMLFASQYACARSWIDSGIQPVAVVGHSFGE
LTSLCVSQSLSLEDAVKMIAARATLIRDAWGPEKGAMLAVEADLEDVQKLLAESSAGCQDVQPATIACYNGPRSFTLAGAVAA
IDAVAEALATPAFSSMKNKRLNVTNAFHCALVDPLLDRLEESARELTFRAPVIPVQRATEYQTEELPTSRFVADHIRSPVFFN
HAIHRLADKYPSCVFLEAGSNSTVTNMASRALGNPSSSHFQAINITSHNGWNNLVDATMNMWKSGLGVHFWAHQPSQTKEYAL
LLLPPYQFEPSRHWIELKNPPKLTAAPAIEEVKKEEAKVPNTLLTFVGYQDSERQQARFRVNTMIPKYDKLIRGHIIAQTAPI
CPATVQLDLVIESIRSIRPELASTEHEPQIHAVENLAPICVNPLRAVWVEVTADDVAQGTSWNFQVYSDDLQNGFSKTIHTTG
RVIFRSISDVSLKYEFARFERHFRHQTCVELMRGGEVDEVLQNRNIYKMFAEIVDYGEDYRGLQKLVSKGNQSAGYVVKKYNP
ESWLDGHLADSFCQVGGIYVNCMTDRVPNDMFIANGIEQWMRSPKMRQQDPRPESYHVLATHHRPSDKAFLTDVFAFDSTTGV
LIEVILGISYVKIPKASMSKLLSRLTVNDSASCPTNMPLLSKSASVNLFDAPENLSTPSLSVAPTQQSAPALSLSKVKKVKND
GPDKGQLTQRIKSILAELSGLEIAEIKDDSELADLGIDSLMGMEMAHEIEKAFTISLPESDLMEVVDVPSLIKCVRKAMSGDA
DSAEYTTEQSTSEAADSDDKSTNYTTPSTPGEEALDMDKSMREFLGKEGTELNLPFETVMKAFNETKNMTDDRIAEYQQTRYV
ESVLPMQSQMCVSLVLEAFDQLNMRIRTAPAGEKFTRISHPKEHTRLVDYLYKMLEDASLINIDGEVITRTAIQVPRPSKEIF
DELVSQHPDQNAADKLTFYTGSHLAEVLKGETDGIKLIFGTQDGRELVSKLYRDWPLNRLFYRQMEDFLERLTSKLDISQGVI
KILEMGAGTGGTTKWLVPLLAKLNIPVEYTFTDIAPSFVAAARKKFSKQYPFMKFRTHDIEKAPADDLIGSQHVIIASNAVHA
THSLSESGKNIRKALRPDGVLLMLEMTGTLHWVDIIFGLFEGWWYFDDGRTHAVTHESRWAKDLQAVGYGHVDWTDGVRPENK
LEKLIIAFASGGRYERLHIPRPLESASADCAARQAVVDRYVQEMTAGFGAATGVSPSAPLAHQEPKGCCVLVTGATGSLGCHL
LAALTSLPTIASVVCLNRRSRQDPLERQHRSLLEKKIFLSEETAARVRVIETDMSKPQLGLLEEEYNYLLNSVTHIVHNAWLM

NAKLPLRRFEPQLQIMRNLLDLAYGISLQRPMEKVSFQFISSIATVGHWPIWTGKSSVPEERMAIESVLPTGYGDAKYICERM
IDETLHKYPDRFRAMVVRPGQVAGSSTSGYWNTMEHFSFLVKSSQTLNALPDFDGVLSWTPVDVVASTLVDLLLLPEDKTPYS
IYHIDNPVRQPWKEMNVVLADALHIPRSNIIPFEKWIQRVKDYPRQVEGAEGDNPAILLVDFLDNNFIRMSCGGLLLETKKSR
EHSKTLANLGPVSAETARLFIKSWIDMGFLSP
>PkiA
MVTPAASQDPPAIPARQNASATAAMAVNAKDTVEQERNVVLLFGCQWLTFTASDFRQLRKAVLDNPELHWMLDVLSELPGYYR
AAAGTSCVPSLRAIRGEEDLRELERWFRCDDLSTAKFPLCYTQLAPLLMMTHFVQYSQWLKMQPNGRNPVVEIVGFCIGLLSS
IAVSATRMGSLKMYGSVAMRLAMLLGAMGDLQQAGEEYTSLAIGWKRPELEDELPAERALTEQSYITVQYDENRATIMAPRRS
VAALQQTLQSAGFSANAVEYNGRYHWPGHEKSLTPLIHLCNTHSGLQLPDASELLHPPRANSTAEPVRSGCLHELVLRAVLAQ
QCLWHKTFSAVYREHLTTPSSIVVEFGPERCVPPTLFRRLPQRIVHFADVELPATISRDHELATRPPAETDIAIVGMACRVAG
ADDLDEFWDLLCSGQSQHREMPRERYANYETPWRPEASHRSWLGNFVRDIDAFDHKFFRKSPREAMSQDPQQRLMLQVAYQAL
ESAGYFSQPSPGKDIGCFIATCTVDYEHNVNCHPASAYAATGLLRSFLAGKLSHHFGWRGPSLCVDTACSGSAVALHHACRAI
LSGDCTAALVGGANAITSPLAYDNLAGASFLSPTGPCKPFDAKADGYCRGEGFAAIYIKKLSHAIADGDQVLATIASTAVEQN
DNCTPIVVPDTASLAGLFKKVTQRAHLHSRDISIVEAHGTGTQAGDPAEYESVRDVLGGPRRVGNLALGSVKGLVGHTEGVSG
IIALCKVVLMILNGQIPPQPGFHSLNPHIRAMPDDHIEIGTRVKPWEVGFRAALINNYGACGSNASMVITQGPQKDEVQERGI
HAENVALPFRVCGLDKARLQAYAARLRRFLSRSERGISFANIAFNLTRKSNPALECQCVFQTRSESELKDILTGLEEGDNKYI
IQVKKPKRPLVLCFGGQVGRSIGLDRTFYNAFPLFKHHLDSCDDILKANGDSSIYPGIFATAPVLDIVQLHTQLFALQYACAR
SWMDCGVEVTAVIGHSFGELTALCISGALSLPDALTLIVRRAVLIRDKWGADPGAMLAVEGDRSTLEKHLESSSANIACFNGP
RSFTVAGPTAVIDFLQEELGADSAFRLKRLEVTNAFHSTLVDPLLPALASAIDGLALNTATIPIERATEHQAADTIPLSIVAD
HLRQPVYFNNAVQRLAARHGPAIWLEAGSNSTITSLARRALGLGVSGNTFHSVNVTSTSALMNLTDVTVGLWSDNVPCTFWGY
HARQTREYAPLLLPPYQFERTRHWMENKPLPLKYNQAQAVMEVSGHTAAKTAPIAPATLLLDYAIELLRSLPNNQRKIPRVFD
VGSDAPLLLDSNREVWIEVSAEDDKRTWALRFQSQTKGGQSDSRLLHCTAHISMHDVRCSRLQTEFTQYARLVSHARCADLLT
DPEVDDILQGRNVYRSFAEIVEYSEQYQGVKRLVGKGRESAGRVVKSYSGKTWADPFLCDSFSQCAGFWVNCMTDRAEDEVYV
ASGIEQWMRTPLYADMATARPDTWHVWARHQQSEGLYTSDVFVFTPDGELVEMFLGLRYSRVAKSLFTRLLRGSTLKVDCRTK
DTANQENNSIKDLVSRVKAVVAEICAVKPSEIQDDSHLADAGVDSLMAMELARELEVAFKCTIALEALVEAETFHDLVQAVQS
ALGETYEDSSVCSGNQCSTTDEATEFPSTSWSITSVSDTADLVLPLDGVLDALDETKGLTDQFLADNKCSGRLLNFTPLMVEM
CIVLTLEALEELGSNIRSARANDRLPRIEFDTQHGPLVEYLYGRLLEAGLIKLDGSTVIRTEICAPTESSSTLLHKIEREYPE
YGGASKLTFYTGSRLASVLRGEQDGLQLIFGTAEGQRLVSWMYGDEPHNVAGYKLMGEFIRRLVDKLPPAAAREGMTLRILEM
GAGTGGGTKWMLPLLAALPVPVEYTFSDISPAFLAQARRKFRDYQFVRYCVHDIEKPPSEDLGKYHIIMASNAVHATSNLQVS
TGNMRQALRPDGVLMLLEMTRPVFAIDLVFGLFRGWWVFNDGRTHAITNEQRWKDDLQAVGYGHVDWTDGESNEVGVQRVIFA
TAGGEQYHPVSPQEDAARLRTVVEYVYQHTAGFTMPALPPRIRAPANHACILVTGATGSLGSHLVARLVQLSNVQAVICLNRV
SRMGPRVRQKEAVAARGLSLESKEETKLMVIETDTANDRMGLSVEQCRYLQENVTHIIHNAWPMNGAAPLSKFEGQFRALRNL
IDLARCIATAQRHPVRFQFISSIGTVNGGGALEERTRIEQVMSNGYNEAKFVCERMIHETLQRYPAVFQATIVRPGQISGSEE
TGYWNTAEHFPAMVKSSQSLGAFPSLAGRLGWTPVDVAARIIAELLLDEGIPEEIYHVDHPTGQNWTTVVDVLAEELEATEVP
FKDWIQRVRNRGGSRENPAGFMADWLETNFERMSCQGPLDTRVARRHSKTLREMGGGGGDEHVRRVVRSWKECGFLTQAQTRQ
GIP
>PksAC
MPHRLPSHGASLGLIFGPQAMNFDSNTFTTLRAKLVKDRHSQWAIDAIAALPAEWSAVSGNVAIFKQYDAGKALQNLNEWLKT
GHVPPADIPFCNVLLAPMVVIDHVISYLEFLQSAFPDLDDDEELPASAKESLETLGLSLGTLSAFAVSSSSTLSEVKKHGATA
IRLAMLVGAVGDAEDLAREPEEGALSFSAFWKSTELHDLLHTSLDAIPEAYISVAVDEKRSTITTSRSKASSHMQDLRSSGLY
IAEVSIRGRFHWDGHESTLQELIQYCDRNLQFQFPDTSRIVLPSHSVTGGEYITQEDGSLHAIALRAILVDLSQWLETITGAY
GSESSKGIKSVVCFGPERCVPSALVRRLGSKLTHVLDVDLPTSALPKQLLQSADVSSTNGVKIPASIKGQSPARQHPIDIDAN
DDKIAVIGMACNVPGGEDMDEFWKILVAGKSQHEELPRGTGRFEFETPWREPYTKTKWYGNFIKDYDVFDHKFFKKGPREMLN
TEPQHRLLLHAAYQTLEQSGYFSKPDYDKHIACFLGPGHVDYASSVNCYAPNAYTATGNLKSMCAGKISHHFGWTGPILTLDT
ACSSSCVAIHYACRSILSGEVSAALAGGSNVLSSVEWYENLSGAQFLSPTGQCKPFDAKADGYCRGDGIGLVFLKKLSTALAD
GDQVYGVIAGSKVYQNVGSTTITVPNADSLATLFKDITKQARIDPAKVSVVEAHGTGTPVGDPAEYEAICRIFGGSQRTDVLS
LSSVKGLFGHTEGASGVCSLLKVLLMMHENAIPPQASFGSMNPGLKATTQDNIEVPTRLTPWKPNTQIALINNYGASGSNSSL
VVTEPPAFETFDHEALQYRAFPFWIPGLDDKSIQRYATRLRAWFQRHHSSSKDLSMRNMSFQLAFQSNRTLPQALVFKAASVS
DLESKLKAFENGTLNSISSAATSQRPVILCFGGQISTYIGLDREVYENVAVLRQFLDQCDETSVSLGFPSVFPHIFQKEPIYD
LIKLQLALFAMQYSCAQAWIACGVEVAAVVGHSFGELTASCVSGTVSLQDAITMIAGRARLIQKKWGTDSGAMIAVDTELSGV
NDLLAKTREGSGADDNPSIACYNGHRSFTLAGTTKSIEQAESILKQDATFSSTRWKRLNVTNAFHSVLVDSLHHDLQSLGKRI
VFNEPKLHFERSTKERMSGKPNSDYIAHHMRNPVYFDHAVQRLAKDFPAAIWLEAGSNSTITSMANRALSSSAASSTSSFHAV
SITTDKSFDLLVDSTLKLWKEQLNVSFWAHHRSQTHQYTPMILPPYQFEKSRHWLETKPPPKPEPIPVEKSTTQAVETSKGFT
SFAGYIDGNQRSLRYRINSNHETFQRHVNGHICAKLAAVWPSSIQIDMVLDALMNLRAEFKDLSYQPQISNIVHHRPLLLDNS
KDYWLELVAKDDKGLTWDWNFSSSSGLGSKSTICTSGVCSFCSATDPNRLAEFQTLERLSSRRRCVELLEARDVENIMQGTAN
IYRAFSEVIEYTDDYRYVKKLVGHNNESAARVVKKHYGKTWLDYLLFDGLGQTAGMYVNLMADKANVSEKGIFMCETINRWLR
SPTIRSHESLPTDWEVYAVHHPISDKKYVSDIFSFDARDGSLVEVVLGASYNKVPLPVMRGILGSQSSTTRLDAITANAEIPS
QAGIGSQQPHLNFKPLSALPALSNGTTGTENPQIKSKTNKVKKVPTRKSGGSDLETPAKTRNILENLTGVEASSINDDSNLID
LGLDSLLSMELIRDVEDIFKVDLDAEQMLDLTDFASLVKYIREIRGVLEEQNVDDSESESEELQQQATPIDSATRQNHEKLTM
NGTGLLTNGESVPEVPLDSTLVLDAFRYIKEASDDFIVKNKFETYCAEFMPRSEEVSIAIFCNAFEELGCPIRTATAGTRLER
VQHLPKHKKVVDYIYKALEKNAGLIEISGEEIIRTSVPCPSEQTEAMLESLLHDRPAQDAEIQLMRITGAAFGKCLAGKADVL
PLLFGSIEGRALLTKLYATSTLSNTILQQLEVFVEKIGSSWPKDGGPLRILEVGAGTGGTTTKIVPVLARLGIPVEYTMTDVS
SFFTATGRTKFKEYPFMKFKTVDIEKEPDAKLLKTQHIVLGSNVIHATRVLSVSLSNIHKMLRPDGLIIYHELTSQLLWADII
FGLVEGWWLFEDGRDHALQSPQHWEKILRSVGYGHVDWTDGTRPEAKIQNLIFAMASDPTYDREPLPTASIMTDVADQVATVN
AYVCQYSSNFQFRRNSASRATGLSSGRCVLITGATGSLGAHLVAYCAERLDVSKVICFNRTSQTAGVARQAKAFKSKGISLEV
NTNPKLEVIETDASKDQLGLSPSEYAALVDSVTDIVHNAWPMSINRGVRSYEGQFRVMRNLVDLARDATMQRPEPFKFGFQFI
SSIGVVGMYPLLTNNFLVPEHRMPVESVVPSGYGYAKLVCERMLDETLHLYPQAFHPSAVRINQIAGSTRSGYWNRNEHLVFL
IKSSQTLNALPDLQGHLTWCPVDTVAATLGELLLDNANSVASAHPIYHIENPSRQSYSEMIRVLADSLFIDHANIIPFYDWVQ
RVRDFEGPVTENPAKQVVDFFDEHFLRMSCGDLVLDTVKSREISATLRARGVITSDLVNKYVEAWRRAGVLR

```
>TropA
MDPLASNFRGETVLLFGSQSLSFDANTFNAIRSSLEKEEYLRWIRHTVADLPSALNTALQHVPHLKGSEEWAFSAVQELNDWL
DSGHQPNSLDPSALPNTILTPLVVILHLSQYMKYLISANDYQDDTLLSKRQQETCETLGLCTGLLSSLAVSSSRTRLQLERYG
SVAIRLAMLIGLIVDARDRSTSHGPSQSTAALWHSEEQKEKLLEILAANPEAYISVYYDQNRATITIPTAQTATIRKDLASAG
LTTTEIGLRGRFHWSGHEAEVDQLIKLCDIDKRFQFTQKTALVLPNRSFDSEPHVHQGPLHAMALWSILVNPPEWQKTVSAVY
ASTLVSTTAKVVSFGQERCVPPTILQNLDSRVFYMGDLEKTSSPRPRGDDIAIVGASIKVAGADDLEEFWEILSKGISQHKEV
PPERFTFDTVYRDRDPKTKWYGNFLNDPDKFDHKFFKKSPREAESMDPQQRLLLQIAYQALEKGGYFHNAGPDQRIGCYMGVC
AVDYENNLACYAPNAFTATSHLRGFIAGKVSHYFGWTGPALTIDTACSSSAVAVHLACQAILKGECTAALAGGTQILTSPLWF
QNLAGASFLSKTGQCKPFDSKADGYCRGEAVGAVFLKKMSAALADGDQILGVISGTAVQQNENCTPIVVPNKPSLSDMFQSVI
EKARLQPDHITVVEAHGTGTAVGDPVEYASVRDTLGGSKRTKKLFLGSAKGLVGHCESASGIISLVKVLLMIQKGMIPPQASF
NTLNPATKATPADGIEISRQLTEWNAPFRAALINNYGASGSNASMVITQAPRATTTIPGGNEMERVPFWFSALNEKSLQAYAA
EFLKYLKANHGQLSLPDLGFNVSRQSNRSLPRRLLFTCQSTNELQQRLEEYVKGDSKTSSSECPATRPLVLCFGGQVSTFIGL
SRQVYEDVALLRGHLNSCNRRCLALGLRGIFPAIFQKGPIEDIVTLQLSLFAMQYSCAKSWIDAGAQPVAVLGHSFGELTALC
VSGVLSLDDALRMIAARAQIIRDSWGSDGGSMMALEADLDVVQKLLATSNANLPENEVAVIACYNGPRSFTIAGPRRAIDALD
TSRQASPEFASIKAKRLDVTNAFHSTLVEPLRNKLTEATKELNFRERGTIHLERSTETRSEKLIDNPGSYVADHMRNPVFFHH
ALQRLDNQFPNAIYLEAGSSSTITNMASRALGGSGGRHFQAMSITTDKGLDNLIDATMSLWNAGLNVRFWKQAFVETENYKPL
LLPPYQFEKSRHWLELKEPPKPEIITMSAGGQRTDKAPDTILSFVGYQDSNKSHARFRLNTENREYQELMKAHLIVYTAPICP
ATVQMDIAIEGLKTLVPGIGSEVQPQIHNVDNQTPICADKSQSIWLDMKLADEPSKISWRFRFFGTSLDNDKMPSNKQADAGV
TFTLGTLVVVPMEDEQTKLDLTRYDLLTGHKRCVELLHSTEAEEILQGRTIYKTFADIVDYGEQYRGLQRLIGHGNNSVGRVV
RAYNPKTWFDAHLSDAYAQVVGIWLNCMTEHDAEELYVARGFEKWIRSPDIQPTSRPDSYDVLAYHKGPSRNSCLSDIFVFHP
KTGQLIEAILGFQFVRIPRKGLAKLLTRLTRDETALATNAQSRAIPPPSTNTTQSSSQQTPIPKAAAPKKEKKRPGNPKLDVL
PKLITILADLVGLEPEEITINSELADIGVDSLMAMEVVTEIERVFSCSVPLDDVADVTTMSQLVRVVESIVGMEGSETSNLSS
DDDDENGTPSTPETDLSDASVDAVVDNAELIAYFAESLGMDASEISANVQLKELGVDSLLSMELGGELVEKFGLNLNESTVLE
DMTINDLRQTAPGAAAPKVAESTITSAPQVTTSKAVPLTNGTSFNIPVETILSAFKETKAAGDSFITATGCRGYVEQVLPEQT
LLCALHTLEAFEKMGSSIRTLKAGDTMTLFTPRPEYVSLIERLTEMLETQIGLIKVIGGPGLTIERTQTPYPTASSTVLMQEM
RQKYPQYQNVNELIFYVGSNLDRALRGETDGIKLIFGCAQGRELVSGLYGDWIMNICYYRQMEDFLIRLIAKLPSNEPLRILE
MGAGTGGTSKWLLPLLARLGCPVEYTFTDLAPSLVAGARKTFKQYASFMKFRAHDIEQEPAEDLLGTQHMVVASNAVHATVSL
VESAKKLRKVLRPNGILMMLEMIEPLYWIDMIFGLFEGWWLFADGRKHALTPPARWKTDLQAAGFGRVDWSDGNLPENSINKV
IIAVACEPEKPEELDRKSVVDEFVHKYTQGVDLLSPAIRVNRKSLGHAVLVTGGTGSVGAHVVAHLAQQPFVTKVICLNRRGK
LDARQRQLESLAQKGLQLSDESLAKIQVYETDLSKPQLGLSPEMYLSLLESTTDIIHNAWPMSIKRQVQGFEAQFRIMRNLIE
FARDISLGGGDPLGFQFISSIATVGHYPLWKQEIRVPEDRLPLDAVLPIGYGDAKYICELMLDKTLHTYPHRFRVSTVRLGQV
GGSKISGYWNPVEHLSFLFKSAQTIQQLPDLHGPLSWTPVDDVAKSLVDLLFTEKPYPVYHIENPITQPWQEMIPILADALGI
PRGNRLSLKDWVARVREFPEDPTDKDKNPATALVDFFEQDFERMSVGGLLLDTTKSREHSPSLRAVGPITPDLVRKFISYWRS
ISFVA
>ATEG_07661
MTHTTGPTKASGESTIFLFGPHVGTFTKQSMNKLVHPLSQSPQRDWILDTIAELPGYWDALAAKMPDVARDIDGSRSLAELDS
WLRHGSANLGEDDSNLPSIIVGPLVVFIQLTQYWRHLELTKAGTQATDLQADLVAMHSNQTGDKVEILGFCAGLLAALAVASS
NNRQEFQKYGAVAVRLAMLIGALIDAQEVWDKASGKGSSSSYAVAWRGPKQEEDMTRIIDDLAANAYIAVRYDQTRATVTASE
TIAPLLLKRFRAAGITVAEVGIKGQIHSPNPDRRAHTNALVDLCNSLPGLQYAAAERLALQTYDNQGDGKPLLPDRGSLTEMV
LRSILVQQCHWYDTFSAVTERHQDPYVVTFGLERCVPPTLMRSLGGRQVFFEDLPKDPSHPSSWMPNAPHGPQQQLQQRQLPV
EVHTKPAFDVSNEAIAIVGMSVKTAGADDLAEFAEMLKTGQSQHIPITRDRLMHDMLFRESADSDPKRKYYGCFFRDGDAFDH
KFFKRSPREAAAMDPQSRIVLQTAYQAIEQSGYFAEDHTGYTPDGRDKAHVGVYLGSCGVDYEHNISCHDPNAFTATGALKSF
ITGRVSHLFGWTGPCMTFDTACSSSAVAIHTACRNLLSGECTAALAGGSNTVTNMNWFQNLAAGSFVSPTGQCKPFDDDADGY
CRAEGAAFVFLKRLSDAVRDGNPILATIASSAVYQNQNCTPLFVPNSPSLSHLFKDVMHQAKITANDVSLVEAHGTGTPVGDP
AEYESIRVALGGPIRKKTLPIGSVKGHIGHTEGASGAIALVKIIMMMREGFIPPQASFKKMNRKIPVRADDNMEVVTKLRPWD
EPHKTALLNNYGACGSNASMIITEPDKALSGPIDGSRYRNTGQRYPFWIPGFDSRAITAYCAKLGSWLRSCRQEPTLADVSFN
VNRQSNRSLTQGFIFNCRSMTELHEKLEQAAAAGKDAAANAGITPVKAERPVVLCFGGQVSRFVGLDRNLFESVAILRQHLDH
VDAVVTSQGLGSIYPEIFEREPVRDTVKLQTMLFALQYACAKSWMDSGLQGKVQAVVGHSFGEITALCIAGVLSLEHTVQLVA
ARAALVRDNWGADPGAMMAIEADENLVNELLLEANRGSDGSASIACYNGPRSFTIAGSTGAIDAVQQTMGSNSKFGSIKSKRL
SVTNAFHSALVDKISDGLERIGKTLTFHRPIIPVERATEMPFDMDNLDGSFVSQHMRQPVYFNHALQRLVKRYPQAIFLEAGS
SSTITIMASRAIAQSQASSSDAHHFQAMSITSDTAFDSLTDATMALWKQGLRVSFWAHHAVQARDYAQLLLPPYQFDTSSRHW
LPMKSPLEEVKKAAAAMVAAGGDVGTGQHQQNDALQDPRLQSLWNFVEFQDGDNKKPRFRINTGSDKYNRFVLSHVIAQTAPI
CPGTLECDIVIEALFSLEPTWKQEGVQPVVRDMINHSPICKDPSRTVYLDLTALNKKRTQWTVRIFSVDSNSSRQASETHAEA
SVEMRAPTDAAHLREFANFERLVSHQQCLDVLRLNLDEEGVEVLQGRNVYRAFNPIVDYGDVYRGVRYVVGRGNECAGSVQLP
KCHRGDTWLDVPLSDSFSQVGGIWVNLLTDLPPSDMYIATGCGLSMRSPTAPPRADTDVWHVYARHSRQGDKAFMTDLFVFDP
ATGQLVELMLGVQYGRVAKASMSMMLARMTKDESVLRTKTPSSSHPAPTVKSVPIEASVAVKASRTTKKKAKASKSKSSVKKD
KAPSGWRDITDEVRNLVATVSGIEASELELDSEMADFGIDSLMGMELGKEVETAFKCTLDQNEQMEATTLRKFVACVSNALFG
PNQGQSSIDEDDEDDEHSEDSSNESSSAASDEDASSGLESPDTGILTPEDEPLPLKAVAIHKAAGLAAIAPPVESHLALSASD
ILESFGEVKMTTDRLMHEYGVHKTEKVMLAGSNRLCAALVVEAFDELGSPLRTAAAGQVIDRVPFLPQHGRLMQWVYEFLERD
ARLIDIDVTSGQITRTHIAPPRKTSHAILQELLASDPDFAVPNRLAYYAGKQLAGVLSGSTDGIRVLFGSPEGRELTAAMYCE
HTFNCMSYAQMREVTKILADRIQSSSGSSGETFKVLEMGAGTGGTTLVMAPLLASLSDMGMAVEYTFTDISPSMVANARRRFS
KLYPFMRFSVHDIEKAPADELKGQHLVLASNAIHATHNLGVSLSNIHQALRPDGFLMMLEMTEVVPFVDLVFGLLEGWWLFDD
GRSHAVVPAEHWERELHAAGFGHVDWTDGSLPENAFQKVIIALASGTQGPRLPKPASVPEPIPELNPKSIETRTAHAEQLTAT
YSKGWATPKLRALDAKSEEGQVKPSGTSRLRKVDLGAVVLVTGATGSLGSHLVQKLADDPNVAQVVCLNRRSNSMPADKRQQE
ALATRGITLSPGGRAKLRILETDTSKAQLGLPPLEYSWLVEHGTDIVHNAWPMSGTRPVSAFEPQLQAMRNLLDLARDMACRD
INPPSRVGFQFVSSIGVVGFVGESRVTERRVPLSATLPSGYGEAKWVCERMLDETLHKYPRLFRPMVVRPGQISGSSTSGFWN
PVEHFAFLVKSAQALRAWPDLDGVLQWIPVNFCAGIIVDLLKIASRADDAYPVYHIDNPVGQPWKAMNPVLASALDIPPHAII
PFKDWISRVRRSPLPLETENPAARLVDFLDDHFERMSCGGLVLDTSKALEHSQTMATVGPVSSDVARLYVASWKKMGYLHS
>CazM
```

MISVADLDYASRKSSIFLFAPHVGTFTKQSMDKLVRPLAASAHRDWILDTVAGLPTYWDALAVKIPNIGNAIPGRRQLTDLDT
WFRHGAGDVTQDDATLPSIVVGPLVVLIQLTQYWRYLELTRPDHLEDSADLQADVVTRQTQPGAKVETLGFCAGLLAAVAVAS
AGNRQEFQKYGAVAVRLAMMAGALIDGQEARDKATRDGGSVSYAIAWRGQKPGEEAARIVKDLNPNAYFAVLYDEARATVTTT
RRTAPSLVNRLRAADVTVAEIGIKGRIHSPDSERKNNTDLLVDLCKSFEDLQYADAASLALPTYNNEAEGRPVSRDRGNMTEM
VIRAILVNQCNWYGTFKGATEGREPFVVTLGLERSVPPTLMRSLGPHQVHYEDLADNGIPPAPQSPPRAVIETQPQPQQQDT
TPPKILENDKDAIAVVGMSIKTAGADDLDEFVEMLKTGRSQHEPVTRERLMHDMLFRAAADSDPNHKYYGCFIRDSDAFDHRF
FKRSPRESAAMDPQSRLAMQAAYQAVEQSGYFTETTAAPEGRDKKHVGVYLGVCGMDYDHNSTCHEPSAFTATGALRSFITGR
VSHYFGWTGPSMTFDTACSSSAVAIHTACRNLLSGECTAALAGGANTITSMLWFQNLSAGSFVSPTGQCKPFDDAADGYCRAE
GMAFVFLKKLSDAVRDGNPVLATIPSTAVYQNQNSTPLFVPNSPSLSMLFSDVMRQAGVAPRDISLVEAHGTGTPVGDPAEYE
SIRTALGGPRVGRTKPLPIGSVKGHIGHTEGASGAIALIKVIMMMRGGFIPPQASFTKMSHNIAVRPDDMMEVVTQLRPWADP
HKIALLNNYGACGSNASLIVAQPPPALRAPTRGAHQHPEGRRYPFWIAGLDARAISAYAAKLIRYLPSSPQDIPTLADISFSM
NRQSNRGLAQGFVFSSRSVAELQEKLAQAAGAAGKEAAALVGIEPVRAERPVVLCFGGQVSLFVGLDRALFDSAAVLRRHLDE
CDAAVTALGLDSIYPGIFEREPVQDTVKLQTMLFAMQYASAKSWMDCGLQGKVAAVVGHSFGEITALCVAGVLSLEDTIKLVA
ARARLVRDSWGPDPGAMMAVEADEGLVRELLQEANRSSDGSAGIACYNGPRSFTLAGSTSAVDAVQATMSADAKFKAIRSKRL
NVTNAFHSALVEKLVDSLGQVGKELTFHPPTIPVERATETPFDAASMDWTFVPRHMREPVFFNHAVQRLAKQHPQAIFLEAGS
NSTITIMASRALAQSQTSPPDAHHFQAVSITTATGFDGLTDATVALWKQGLRVAFWAHHALQTLEYAQLLLPPYQFDTSPSSR
HWLEMKSPQKVIDKAVKALVAAGTPHQLPQNQTIDPKTLPLWTFVGYQDAPTNKTARFRINTESAQYNTYVLSHVIAQTAPIC
PGTLECDMMIEALFSLHADAQENGLQPEIRDLVNHTPICVDPSRTVYLELTALYKKRTQWSAHFLSVANGGGDAHNPQTHAEA
RLRLRAAGDADYLREFAQFERLVSHGRCRELLGLGLDVEGVEVLQGRGVYRAFDPVVEYGEVYRGVRYLVGRGNECAGHVQLP
ARHRGDTWLDVALSDSFSQVGGLWVNLMTDVPPGDMYIATGCEVTMRAPRATARGEGDVWHVYGRHARQSDKAYTTDLFVFDA
ATGSLVEVMLGLQYGRVAKASMSRMLARMTTDETVVRTKASLPQLPATNSSPPATAPAPAMAVVEASRNSKKRSKKTEKKEAK
PKKTQKDSGWRDITEEVRNLVAHVSGIEASEIGLDSEMADFGIDSLMGMELGREVELTFKCKLDQAEQMEATSLRKFVAVVAK
ALFGTDQPAEVEDEASEAEDEDDDDSSSTGEGGTWSEPSQEDGSKESSGLQTPDSSSYTPPPEKAQKAGPVPGNLKPLNAPTA
PPAVSNLVLSPADVLAAFGEVKMATDSLMREYEIDKTERTLLAGSNRLCAALVVEALDELGCPIGTAAAGQPLERVPFLPQHG
RLMQCVYEFLERDARLIDIDVASGQLTRTHVAAPRKTSQAILEDLLTSQPEFAVANRLAYHAGKQLAGVLSGKTDGIRVLFGS
PEGREVTAAMYCEHPFNRMSYRQMSDVVEGLAERIQSRGGTGETFKVLEMGAGTGGTTLVMAPLLASLEARGIMRVEYTFTDL
SPSLVANARRRFGKMYPFMRFAVHDIEKSPAEELRGQHLVLASNAIHATHNLVVSASNVRQALRPDGFVMILEMTEVVPFIDL
VFGLLEGWWLFDDGRNHAVVPAEHWERELHTAGFGHVDWTDGNLPENAVQKVIFAMASEPQGPRLPKAAPEQTMELDRGDVAA
RTAEAERLVTKYSNAWATPRLQALAARRETNQGQPKRNKANLGAVVLVTGATGSLGSHLVQKLAENPEVAQVVCLNRRSSSGL
SAVKKQQEAFASRGITLSPGARAKLRVLDADTSQAQLALPPHEYTWLLQHGTHIVHNAWPMSGTRPISAFEPQLQGMRNLLDL
AREMATRDAVHPFRVGFQFVSSIGVVGYAGEPRILEDRVPLSAVLPSGYGEGKWVCERLLDDTLHKYPSLFRPMVVRPGQISG
SSNSGFWNPVEHFAFLVKSAQTLRAWPDFDGVLQWIPVDHCASVMADLLKMGVADAPDAYPVYHIDNPVGQQWKAMSPVLAAA
LDIPPHAIIPFSSWIKRVRRSPLLAETENPAARLVDFLDSHFERMSCGGLILDTTKAKEHSQTMAKEGPVDPDLARLYVAAWK
KMEFLRS
>AfoE
MTRASASGSGHEASTVFLFGPHVGTFTKASMDKLVRPLSQSPQRDWILRTIADLPTYWDALAAKMPDIARDIDGPTSLSELDR
WLRHSLDTAGLSVSDDESLPSILVGPLVVLIQLTQYWRHLEMIRDGSAPAVDLQAELVQQTQSGSRPTVILGFCAGLLAALSV
ASARNQAGFEEYGAVAVRLAMLIGALIDAQEVWDKASGKGSSASYAVAWRGQKQEDEMNRIIGDLSNDAYVAVRYDQARATVT
ASETIAPLLMKRLRAAGVTVAEVGIKGQIHSPNADRKQHTNALVELCASLPGLQYAEVSKLALQTYDNQGSGIPVSGSGNMTE
MVVRSILVQQCRWFDTFSAVADALPDPYVVTFGLERCVPPTLMRTLGGRQVFYEDLPKDPEKPSFWLTPQSSPPPQPQLQPVL
QLQQQQTTRVEPVMPVSPQSEPIAIVGMSVKTAGADDLDEFVAMLKTGQSQHIPITRDRLMHDMLFRENADADPKRKFYGCFF
RDGDAFDHKFFKRSPRESAAMDPQSRIVLQAAYQAVEQSGYFVEDHNGYTPDGRDKMHVGVYLGSCGVDYEHNISCYDPNAFT
ATGALKSFITGRVSHHFGWTGPCMTFDTACSSSAVAIHTACRNLLSGECTAALAGGSNTVTNMNWFQNLAAGSFVSPTGQCKP
FDDDADGYCRAEGAAFVYLKRLSDALRDGNQVIATIAASAVYQNENCTPLFVPNSPSLSHLFKDVMRQAKVTANDVSLVEAHG
TGTPVGDPAEYESILAALGGPSRKKKLPIGSVKGHIGHTEGASGAIALVKIIMMMREGFIPPQASFKTMNKKIPVKADDNIEV
VTRLRAWEEERKTALLNNYGACGSNASMIVTQPDLRGPHSRSHAVAGARYPFWIPGLDTRAITAYCAKLGPWLRSRAEEPTLA
DISFNLSRQSNRGLPQGFIFNARSLAELHEKIEQAVAAAPSSKDAAASVGIAPVKAERPVILCFGGQISRFVGLDRGLFDAVA
LFRKHLDAVDTVVKAQGLVSIYAAPDIFSREPIEDTVKLQTMLFAMQYACAQTWIDCGLNGKVQALVGHSFGEITALCVAGTL
SLDETVRLVAARAKLVRDSWGADRGAMMALEGDEGLVHQLLSEANGASGSDGSASIACYNGPRSFTIAGSTSAVDQVQQTISR
PEFGSIKGKRLNVTNAFHSSLVDKISDGLDSIGKTLTFNSPLIPVERATEVASARATDASFVSQHMRQPVFFNHAVQRLAKRH
PQAIFLEAGSSSTITVMAGRAIAQGQASSESHYFQAVSITNETALDSLADTTTALWKQGLRVTFWAHDAVQTAEYAHLLLPPY
QFDTSSRHWLPMKSPVEKVKEAALALIAANGGSLAGAGLQGQQAGTPQDPRTLPVWEFVGYQDDETRQARFRVNTSADKYNRY
VLSHVIAQTAPICPGTLECDIVIEALFSLEPGWRQDGVQPVVREMINHSPICKDPSRVVYLDLTATNKRRTNWTVRIFSLDDD
ATKKTPEIHAEATVEMRSSSDQAHVREFANFERLVSHKQCTDLLRLSLDQDTDEGVEVLQGRNVYRAFSSIVDYGEVYRGVKY
VVGKGTECAGRVQLPRSSRGDTWLDVPLSDSFSQVGGVWVNLMTDLPSSDMFIATGCELSMRSPRAPPREDADVWHVYARHSR
QGDKAIMTDLFVFDAVSGQLVEIMLGVQYMRVAKASMSMMLARMTKDDSVLRTKALVPGPTPAAAFQAALKTAPEVRASSEPG
AKVKASKTSKKEKKEKKPVTKAKSKSSKPSGWRDITEEVRNLVATVSGIDASELELDAEMADFGIDSLMGMELGKEVEAAFKC
TLDQNEQMEATSLRKFVQCVSNALFGPNAGPAEAEDDEDEEKSDNSSSESASESDDAGSESSDTGILTPTGEEEQPLPLKAVA
IHKSAGLAAIAPPVESRLALSSSDILASFGQVKMQTDTLMKEYGVDKTEGVMLSGSNRLCTALVVEAMDELGCPLRTASPGQP
LARVAFLPQHGRLMQWVYEFLERDARLINIDPASGQITRTHITAPRKTSQVILQEVLASDPGFAVPNRLAYYAGQQLAGVLSG
STDGIRVLFGSPEGRELTAAMYCEHTFNCMSYAQMREVTNLLAERIGRTGETLKVLEMGAGTGGTTLIMAPFLATLAESGALP
IEYTFTDISPSMVANARRRFSKQYPFMRFAVHDIEKPPADELRNQHLVLASNAIHATHNLGVSLSNIHQALRPDGFLMMLEMT
EVVPFVDLVFGLLEGWWLFDDGRHHAVVPAEHWESELHRAGFGHVDWTDGNLPENTFQKVIIALASGAQGARLPKPGPVQTLI
PELNRENVEARTATAESLVAKYTAGWETPKLRALASRAEKESGKTQAPHAAPGRRAHEAVVIVTGATGSLGSHIVQRLAETPS
VATVVCLNRRSSSTTPEKRQQAALTARGITLSPGARAKLRVLETDTSKPQLGLPPLEYGWLLENATDIIHNAWPMSGTRPVSA
FEPQLQAMRNLLDLARDIAERPFNGSSRVGFQFISSIGVVGFCGQSRVSEDRVPLSAALPSGYGEAKWICERMVDETLHRHPG
LFRAMVVRPGQISGSSTSGFWNPVEHFAFLVKSSQSLRAWPDLQGQMQWIPVDYCAAGVVDLLHLTSRGDEAYPVYHMDNPVG
QNWQAMNHVLASALDIPASNIIPFKTWISRVRRSPLPMETENPAARLVDFLDDHFERMSCGGLVLDTSKAKEHSTTMAGVGPV
GTELARLQYQARSSLLISLEKLQCVYHSVANYSVLVTMGLRRRSSIATPYTPQI

```
>SAT8
MATTLLLFGPQAASMSKQSITQLQVALRDQEWAFDALSNVQPIIQRASTSISGLDQISLDERLADLTRWLKHGPKDQEELAEI
PNIMLAPLTTLSHLVQYRRYIERHYPNESDAHAALLQQKPVATLGFCNGLLAAFATTSSATLNDWERYAAVATRLALLVGAVI
DAADELQPHGPAASYGVSWRDIDGARQLEQILSPFPGDAYVSVWYDRSRATVTVSKHLVRTVLHLVEAAGMAVVPVRLRGRYH
SRQHAEVAEALIRLCDAEPDLLALPDARNLCLPTYSNVGHGEVVREGRLHEIALQAMLVQQCDWYSTLSGITDESQVQVVCLS
EVSTLPPSLTFKLKPQMEYFAPLEEKTAPKDNFSGRADGGSQFSFSMLENSTSPPSPAATSSNSHCEYSVDPRDIAIVGMSVK
VAGADDVVEYESILRGGVSQHQQVRKNRVPFGYNSFRPEEPGHKWYGNFVRDVDAFDHKFFRKSSRESAAMDPQQRLVLQAAY
QAVEQSGYYASGTEPDQHIGVYLGTCATDYEQNANCHAPGAFTVTGLLRGFIAGRISHFFGWTGPAMTYDTACSGSAVAIHSA
VQALVSGECSAALAGGVNTIGNEVWFQNLAGAQFLSPTGQCKPFDDAADGYCRGEGIACVVLKPMAKAIADGNQIFGRIASSA
VHQSVNCTPLFVPNVPSLSRLFGDVMRQARLEPHDISFVEAHGTGTPVGDPVEYESIRAILGGPLRDKPLSLGSAKGLVGHTE
STSGVVSLVKVLLMMQSGFIPPQASYSKLSHRIAPSASAMIQVSTTLQPWTDSYKAALINNYGASGSNAAMVVTQGPAQTARS
PRGEADGAHLPFWIPGFDSARIAAYCARLSAFIEANRSTIHLADIAYNISRQSNRTLSHALLFRCNSIDSLVGQLSSAAAPQT
VQVKPSRPVILCFGGQMSTFVGLNREIYDSSPILRDHLSQCDAAIRALGFGSIFPSIFATIPIEDTVLLQTVLFSFQYACAKS
WIDCGVRPTAVVGHSFGEITALCIAEVLSLDDTIKLVTRRAKVVRDSWGADRGVMMAVEGEVDQVERLLEEANKDLDTHSPAS
IACYNGLRNFTLAGSTLAMERVALALSSSAYASIRGKKLNVTNAFHSALVDPLLQELEQAGSDLTFNKARIKVERATKESTTG
EPCAPKFVGEHMRNPVFFRQAAQRLARDNPSAVWIEAGSATKITAMARRSLDSNAESHFQGITVTGEDGLDKLTEATLSLWKQ
GLNVAFWAHHGPQATRDHQPLLLPPYQFEKSRHWLDVKAPPVMLADTAQGDNGPLFGLLTFVGFQDAEERRARFKINTESERY
KSLVIPHIIARTAPICPATLEYSLAIQALLTLRDHKHFESRDMHPVIRDMRNDAPLCLNSDQSTWLDLEANKTSPRSLVWKVF
TAPVSRQLDSHNDSDETLCAQGKLDLLSSSETTEFAQYEQLATYDACVSLLQDDDGDVSGLQGRSVYRSLADVVDYGVHYQKV
RRVSGRNSESAGIVRGASGGNWLSDLPIIDSFSQVAGVWVNCLADRTPGSDDLFLATGCETIMTSPTFLHADRGGKSWHVWAK
HHRESERSYRTDVLVFDATNGQLADVFLGIAYTRIPRHSMTRLLSKLSEPSALQAQAALPSSTGHEGLTAKTASSQRLGQDTL
KQTVGQIIASLSGVEAAQITDESALADIGIDSLAGMELARDIESVLGCKLDLEELLFTHDTFGAFVRYISKVVNGEDDLGTPS
HSDNDSHVTGTTATPNSSSASSDTHHGNSKLQIAVAQSSQADASSSSPLPPQHVISSFEQVKLSTDQRIREEKADNTDDIIVS
RSNLLCVALVVEAFEQLGCPLRGVPAGEALKRIQHAPQHARLVDWLYRFLEDEARLINTEGTLILRTSNGAPNKTSQAIFQDL
EHANDRWIESHRLANYAGKNLADVVSGKKEGIHVLFGSAEGRELVRGLYSGLPFNCLFYKQVRDTISLIVEKVKDDFQGPLRI
LEMGAGTGGTTQVLAPFLATLDIPVEYTMTDLSPYMVAQAQRSFGTKYPFMHFAVHDIEKPPAESLLGTQHIIVASNAVHATA
NLADSAANIRSTLRPDGILLLVEMTESLPFVDIVFGLLEGWWRFADGREHAIVPAEQWEARLRDAGYGHVDWTDGVFSENRLQ
KVILAMASELPDGLPVSSGVPEPVQPALEVTTTVAREANAEAYVTQYSADFTYDGESSGNIEAHEAQDSRIVVVTGATGSLGS
HMVASFAESPSVTSVVCINRRNSGKATALERQQEAFTSRGITLSPDAFGKLRVFATDTAQPQLGLPLEEYEWLVTHATHIVHN
AWPMSASRPIQAFQPQFKTMSRLLDLAAAIAQQSTSRFVVFQLISSIGVVGSAPMIDTRVPERRVPVSYTLPNGYCEAKWVCE
QLLNETLHQYPERFRAMVVRPGQIAGSSVNGVWNPVEHFPALVRSSQALRAFPALGGTLQWIPVDVAAGTVADLALNQQAGEP
VYHIDNPVGQSWSNMVPILADELNIPGERIIPLGEWVRKVKRSSLLETENPASRLPDFFEQHFERMSCGGLILDVALATKRSG
TLAAQGAVSADTARKYIQTWKDMKFLDRY
>LijE
MGSIEHRDQQESTVLLFGPQALTFNRQFEKLRQSLSGNDVGQHWILDAVAELPRYWTALTEKMPKINGAVEGEKHLADLDSWL
RQGPLEDDRPAGLEQLPNITLTPLVVLTQLTQYWRYLEFNHQLKGDGAKEDLQADLIARAKSGSSNKVQTLGFCTGLLSAFAV
ASSNNQDEFEKYGKVAVRLAMLVGALVDAQEAWSKELGQGPSKSYATAWRNAKQEQELRQIIDNHFPQAYISVLYDESRATVT
TSQRTAPAFLQAARQSGITAAEVGLRGTFHSSDSLHLTNTLVELCDSIAGLRFPDASQLALPTYTNDATDGHSIPASAGSLTE
IALHAILVQQSKWYNTFAAVQSNLNPILVSFGPDRCVPPTLMRRLGPRLMQFADLDDEMPQRLASVLDPEAHQQHSQSQLQID
EDAIAVVGMSVKVAGADDVDEFSQMLRTGQSQHEEITKERLMMDTLFREGDKDPKRKWYGNFIRNSDAFDHKFFKRSPRESLT
MDPQQRLFLQAAYHAVEQSGYFTETTPSTAARDKKHVGVYLGACAGDYEHHAAGHTANAFTATGNLKSFIPGKVSHYFGWTGP
SMTFDTACSASAVAIHTACRNLLSGECTAALAGGVSTITNFLWFQNLAGASFLSPTGQCKPFDQAADGYCRAEGIACVFLKKM
SDAVADGNQILGCIPSTAVYQNQNSTPLFVPNSPSLSQLFTDVVKKAHLSPRDISLVEAHGTGTPVGDPAEYESVRLALGGPI
RSKPLVLGSVKGHIGHSEGASGVISLIKIIMMMQEGYIPPQASFSKMSSYVKTSPTDMMEVSTSLRSWNEDFKAALINNYGAS
GSNASMIVTQSPHSSSGQASAFIRGESVNNASFPFWIAGLDARSINAYCAKLLPLLKSKEKAGISLADISFSVNRQSNRSLAQ
GLMFSSRSISELEEKLSKSSDAIAPLKAERPVVLCFGGQVSTFVGLDRKLYDSVALLRYYLDQCDTAMQSLGLSSIFPDIFSR
SPISDPVKLQTMLFASQYASAKCWMDSGLSGRVVSLVGHSFGELTALCVSGGLSFRDTVKLVAGRAKIVRDSWGSDSGAMMAI
EADEQQVHKLLEEANRLYAGEYPASIACYNGPRSFTLAGASKAIDAVTETIPQFGGSIRSKRLNVTNAFHSTLVEPLVGRLEA
LGKTVTFSAPVIPLEHATENRPVAGSRISSTFAAEHMRKPVFFNHAVHRIAKDHPSAIWLEAGSASTITIMASRALAGETRPD
SHHFQALSLTNTEKGLDGLTDATLALWKQGLPISFWAHHSMQTTEYATLLLPPYQFDKVSHWMELKSPIKAVEEAAAALAQTM
TGPVVVQQQPVDEKFLGLWSFFGYEEKKPRFRINTGSDKYKSYISGHLIAQTAPICPATLQVDMAIEALFSLHPEWTASAKQP
VVLDMSNHSPLCVDPTRSVWLEYEALEDSDGLWGWKIFSTSSSGSSDTHVEAKLHIRSPDDPTYQSEFGRFERLVTHAQCASV
LAMTDDDDVDILQGRNVYRAFAEVVDYGELYRGVRRVVGRTGECAGRVQMKKSGETWLDVPLSDSFSQVGGLYINCMTDRPAN
DMFIATGCEMSMRSPRMAKLDKNEFPENWHVLARHHRQAEKVYTSDVFVFDAATGLLTEVMIGITFARVAKASMSKMLTRLTN
DDSVLKVKPAQTHAQIVQPQPLTNVTLANVNIEAPVAEKTSSKKAKKEKTKENAAKSSRPDVSNDLRNLVANISGVEPEEIGL
DTEMADVGIDSLMGMEVAREVESVFKCTLDQTELMEATTLRKFVTCLNNALYGPGGGDAAPEASEEDEDDDSDSSSSSSQGLS
EYTSADTRATTPGIQEFKPAIVEEPPKPAAAIAIPPAQSNLSLSRSQILQTFGAVKMLTDQEIKHFNLDRIHKVVLAGSNRLS
TALVVEQFEKLGCHLRKAEPGQTLDRVPFLPQHGRLMEFVYNFLERDTRLIDIDVFSGEITRTNIPVPRKTSDVIFAELLQAF
PEFQVANKLTYYAGQHLADVLIGKTDGIRVIFGSLEGRDLVQGLYVNHTFNRMQYGQMRDTIQRLVANLPTNDGPLKILEMGA
GTGGTTYVLAPMLASLGFPVEYTFTDLSPSMVANARRKFGKEYSFMRFAVHDIEKAPVEELQGQHLVVSSNAIHATHNLAASL
TNVRKALRPDGFLMMLEMTESAPFIDIIFGLLEGWWLFDDGRKHAIVPAEHWERDLHAAGFGHVDWTDGSLPENIYQRVMIAL
ASGSQSERLPKPMPEETPAEGERDVVAREAEANSFLAKYVNGFAAPSSSDGSLASFGGVLRDGQDAVVLVTGATGSLGSHAVT
SFAENSAVKTVVCVNRKASTPVKQRQSDAFSSRGIELSADAQGKLRILETDTSKPQLGLPADEYAWLVRNVTHIVHNAWPMSG
TRPVKAFEPQFQGLRNLLDLARDVADYRYSDAGFRVGFQLVSSIGVVGNAGKARVPEDRVPFSATLPVGYCEAKWVCERMLDE
TLAKHPQRFRTMVVRPGQIAGSRISGFWNPIEHFAFLIKSAQTLRAWPDFDGVLQWVPVNDAAGVMADLCHVAELHNAPEPYP
VYHIDNPVGQPWKEMNPVLADALGIPADRIVPFHDWVNMVRRSPLPAETDNPAARLIDFLDENFERMSCGGLILNTTKAKEHS
ATMASMGPVEGAVARKYVASWKKMGYLTRRF
>EupA
```

MQTEEWKMANQDNNKPQRDSERCVFLFGSLSLSFDASAFAQVRKAIANDERNSWLVNAARQLPQDLETILSGLPSLNNSNTRA
RKQLADLHNAIIGGRPLDTPFPLPNTVLIPLVVIEQLSQYADFARQKGVERSGTPDGWPAIEADTKSLGLCTGNLAAFATSSA
RSWGDFQKYGAAAVRLGLLVGLVIDSQDEAFDSRRWRSLSVAWSGSQGGEELQRILPEFDETYVSVYYDACRSTITTPVSSLP
TLVHRLKAAGLGVTEITLYGAFHSAARNSAVLEQLTVFCDSHADFQLSTSAASVAALQANDNTDHKDIIRKQGALHAKALRRI
LVEPAQWFDALAAVLGEEGERARVVSFGSERSVPLSLAMRSNVRVVHATDTHDGNRGTNADGGERMWAESDIAVIGMACKVAG
AEGVEEFWELLVEGHSQHRDISASERFSFDDTAFRTASDSNMQRKWYANLVDGHDQFDHRFFKKSARESAAMDPQQRQLLQVA
YQAAEQAGCFTRTNSVCDGNVGCFVGVCLSDYESNVGCHPANAFTATGNLQGFIAGKVSHFLGWTGPALTIDTACSSSLVAVH
QACASILAGECNSALAGGTHIMTTAGWFQNLAAGAFVSPTGQCKPFDAAADGYCRGEGVGAVVLKKLSQAIADGDRVLGVIGA
TAVQQNQNCTPIFVPNVPSLSALFTTVTAKAHVKPASVSVVEAHGTGTAVGDPAEWASIRQTLGGLNRPPERPLMVSSTKGLV
GHLECTSGIISLIKVLLMVQKRMLPPQASFDTLNPVLNAQPSDHMFVPRRAQPWDAEFRVALINNYGASGSNASMIVMQPPTF
DTVAPIHLAAAVRSPDCRYPFWLSGLDSSSLRRRAKALRRFLSRGLGPASSLAPSLASISYNLAHQGDRALDQRITFTAASVT
ELDQHLAACEDGSDNKPAAPASTAASKQTVVMCFGGQVSTHIGLDPHVYNSVGLLRQHLDNVDTIIQSLGYTTIFPAIFQRVP
LADTVHLQTMLFAMQFACAQAWIDSGLRPTVLVGHSFGELTALCVSGALSLVDAVKMIARRAAIVRDAWGIDRGTMMAVEGDL
YEVEQLLVDVNSSISPAVPISIACYNGPRSFTLAGSTDTINEVDARLCAQPGRSIKSRRLNVTNAFHSALVDPLLHRLEESCE
DLTFRRPVLYLERALDSTSSGPEWSARSVAEHMRNPVYFHHALQRIVQIDPSSSFVFVEAGTNSSITAMTSRALDNKIIKGTS
TFHGLSIANCDDGWNKLTDSIMSLWKAGLNVQHWAHHQRQRRYQADIEPLLLPPYQFDPNARHWMDLKPLPRQLPALIEEATK
TEADKKPEGLLTFVGFQDSSTQMQAQFQINTNTEEYKSLLLGHMTIQTAPICPATMQISFVIEAIVTIRHELQTEQEPQIQDV
QYRSPVCANLSRKTWIEIKDENERGLAWRFEVFSTESHSGPRTIHTTGKIKFTNARDATLQRQLMQFERLFGHHRATDLLKSA
EVDEVLANRSIYLIFSEIVDYGEEYRGLQKFASKGHESAGHVVRRHSHDRKTPRFDAHLADTFCQLGGIWINCMTERSQDDIY
LANSIDQWIRSPHNAGAAGSTVEPSKEYHVYATHHRPSDKLSLTDVFVFDVKAGKLVEVILGIAYVKIPKLSMQKMLTRLTGS
EWLNNTPATSMNQAPVSAPNPATRPSIVDLPSAVTQVPVLLPQGVKPSAEIRPSEPPSRSLLENITERVKAVVADLSGVEVTE
IGDECDLADLGIDSLAGMQMVHEIEGALHVTLPEKEILLVVTMRDLMKVVTGVVEVDFEATDSLSSDNDLPSIATSSEGERVA
TNLTTPAPQSDVDKDEHEAFESNDLRLPAAVVLEAFKETRELTDEHVLGVGQASYVSEALPLQTELSISLTLEAFEALGAGLR
NAGPGEQLFRIVHDEEHARFVDYMYDMLEIETQIIKVDRGIITRTAVPFPERSSTVVYAELRRRFPDQRAADELTYYAGTHLM
QVLSGETTGVKLIFGTTEGRELVSTFYGEWPLNQVMYTQMEDFFTRLASKLPATNDNKPLRILEMGAGTGGTTKRLVPLLARL
QIPVEYTFTDLAPSFVRAARNKWEKLYPWMRFRVHDIEKAPGEDLVNTQHFVLASNAIHATRSIRESTLNVRKFLRSDGYLLM
GEMTRTPYWVDIIFGLFEGWWLFDDGRRHALTHESRWETDLQSAGYGHVYWTEGTRPESEIEKLILAAASLTNLAGSDFVQSE
RNPITKRHQFDNEVSGGCAEREKLIMEYVCDSTQDFAKTFKVPMSSIPRSLHHKSKGKWIVVTGATGGLGAHLVAKAAVRSDV
ERVVCLNRRSKQNALERQMHALRKQGISLDSECLAKLDVHVTELAQPSSLGLPNELYNLLLDNVTHIVHNAWLMNSKWTVKRF
EPQIRIMKHMIHFARDISLRHTESDPVTFIFVSSIATVGFHPLLAKSPIVPEDRVSIDSVLPTGYGEAKYICERMLDTSLHRY
PSRFRAAAVRLGQIAGSSLNGYWNPMEHVSFLIKSSQTLRALPNLPGSLGWTPADAIADSLLDICTQPGDVALHPLYHIDNPV
RQNWDEMLAVLADVLNISQGASGIVPFDEWLRRVRDWPHTEDNASDGKNPAYVLVDFLEDHFVRMSCGGLLLGTRKAREHSET
LACVGPVDAQAIKLYVRSWKDMGFLD
>AzaA
MVTTTRATNPTNTLLLFGPQALSFSTATFADIHARVVQTSENAWIKQTITSLPGLWDALVKEFPQYGALEGKQLLRDLDRWFE
TGTMEHAEPHLPNILLSPMVVITQLTEYVDYLKTMPHAADQQTETVGFCTGLLTALAASLAPDIKGIRQYGAIAIKLAMIIGG
VVDVQDITSPNGPSKSLAVAWDSAETQDRLNQIIDQSPEAYISVEYDHNRATITTAARSISSLQQRLRNAGLIASQIGLRGRF
HCACYEKDIEALSKFCDSVPSLCLPDAAVLALPTRSNDAGSFILSGKLHHCALRSILLDTSHWYQTLEVVRQSFLKSPSSMVV
SFGPERCIPPSILKGLSSIVTTAAEYQPSYLHRDPELCNPNEIAVIGMSCKVAGADDVDEFWDLLCKAESQHQEVPKERFGFE
SAFREVDPTRKWYGNFINEHDCFDHKFFKKSAREIAATDPQQRQMLQVAYQAVEQSGYFTTPKSDKDRKIGCYIGVCAADYEY
NVACHPPNAFMATGNLKSFVAGKISHWFGWTGPGLCIDTACSSSLVAVHQACQAILTGDCTAALAGGANIITHPLWYQNLAAA
SFLSPTGQCKPFDASADGYCRGEGFAAVFLKKMSAAIADGDMIIGSIKATAVNQNQNCTPVFVPNAPTLSDLFRDVLDRSQLT
ANQITVVEAHGTGTQVGDPAEYESIRNVLGGPSRSTPLLFGSVKGLVGHTECTSGAVSLVKTLLMQQHEAIPPQPSFDRLNPE
IPVSESDNMQIATRFSPWTAEYRAALINNYGACGSNASMVVAQAPRTEQKRSATRRTSVVLDYPFRLCGSDDRALRAYSERLV
RFIASGIKDGISVADLAFNVCRQSNPTLDRSLAFACRTTQEVEEKLRAFVAGNQGLISASRSKTPREVILCFGGQISNYVGLD
REVYDNVALLRKHLAICDAACRDLGVDSIFPGIFQKSPISDPVKLQTILFSTQYSSAKAWMDSGVRPVAAVGHSFGELTALCV
TGILSLADAMKMIVGRATVIRDFWGEDKGSMIAVEADENRVQRLLAEAAKQCELIHVRAPTIACVNGPTSYTLAGPVKSIDIV
TEVISRLSDSGPSIRSKRLKVTNAFHSTLVEPLMEELEKVGQQLTFNAPTIQLERAIEHHSDATLTSDYVPDHMRNPVYFNQA
VQRLAQQYPDSVWLEAGSNSTITSMASRALGSPKSLHFQAVNITSDDSWSMLITSTLSLWKQGISTNFWAYHAKQTYEYNPVL
LPPYQFEPSRHWMELKVPSFMSNGNVQCGPRDEEEPPKTLWSLIEASDKVARFQINTAAPKYVELVSGHVIANTAPICPATVE
VDIVVEALRSLRPDFMDSNLQPQVLAVTNQSPICIDPNRSVWLECQAMDSNLVWEWRIVSDSLQEPGTSSSAHVLGKLAFLSG
QDEVKQQESEFMRLERLIGHQRCVDLLNTTEADDIIQGRNIYTTFAGVVDYGEQYRGLKKIVGKGLESAGRVQKKPSEESWLD
AHLGDCFSQVGGIWVNCMTDHNPDEMFIATGFEKWVRSPALRHGQPRPEIWDVLACHHRSSEQTYLTDIFIFDAEQGALTEVI
LGINYHKVAKASMSKILSRLSGTEAAPSSSTRAHPTSSSSPRLPGPFVPEDKSQNETQTAGTNAVAKKKSEKSAQQNVLDKTR
ALLAEISGLEPSEIEAETGLADIGIDSLMGMELARDLEALFKCPLLGDELANVTTFQGLVEYVQSAVGVPANGDEPDNTNADE
VFEEDNLAASPSSSSSSTNLTEDSSLDPTETTTNISSYPELSPAWVLEAFEESKQLTDHFIEQYRCANYVDTILPKQTQLCVA
LTVEAFEKLGCPIRSAVAGQKLERILHIPKHAQLAQYLYRLLSADARLINLTEDGRITRTHMALPKPSDQILQDLLRLYPDHE
WANRLAAFTGARLAEVLKGETDGLGLIFGTDEGRELVAGLYGDSLLNKLSYRQMEDIITRLASRIPRDSGPLKILEMGAGTGG
TTRGMAPLLARLGIPVEYTFTDLSGSFVAAARKKYQKEYPFMKFQVHDIEKPPSDQLRHSQHIVIASNAIHATHSLTDSSRHV
REFLKTDGFLMIVEMTQPVHWVDIIFGLFDGWWLFADGRDHAIASAGWWEKVFQSVGYGQVDWTDGHRPEVQIQRVIIALASG
PRYGRQPLPPAPPPNLVPGSHASRQAAVNEYLDKYTKGFTLPAQTSNTDISNSTSYWEKQCVLITGATGSLGVHLVAAVAALD
DVQTVICLNRRSPMDPDLRQQQAFERRGILLEAASMSKIRVLQTDSSKPQLGLTDEVYSSLVTSTTHIIHNAWPMTGKRPLSG
LEQQFLVMRNLLDLAAQCSSTRPANAPRIVFQFISSIATVGYYPLWSGQTLVPEARMGIESVLANGYGEAKYVCEQMLDRTLH
QYPDRFRAMAVRLGQIAGSRTSGYWNPMEHLSFLFKSAQTLQVFPDFTGDLCWTPVNDVAATLSDLLLRSTHSNSTTDQPIYH
IDNPVRQSWSEMVPVLIDALGIPAQNVIPFADWVCRVRAFPGQVEWDNPSALLIDFLDDHFLRMSCGGLLLDTKRACEHSPTL
AAVGPVTAELARKYIQSWKEMGFLNP
>EpaA
MVTTTRATNPTNTLLLFGPQALSFSTATFADIHARVVQTSENAWIKQTITSLPGLWDALVKEFPQYGALEGKQLLRDLDRWFE
TGTMEHAEPHLPNILLSPMVVITQLTEYVDYLKTMPHAADQQTETVGFCTGLLTALAASLASDIKGIRQYGAIAIKLAMIIGA

VVDVQDITSPNGPSKSLAVAWDSAETQDRLNQIIDQSPEVYISVEYDYNRATITTAARSISSLQQRLRNAGLIASEIGLRGRF
HCACYKNDIEALSKFCDSVPSLCLPDAAVLVLPTRSNDAGSFILSGKLHHCALRSILLDTSHWYQTLEVIRQSCLKSPSSMVV
SFGPERCIPPSILKGLSSIVTTAAEYQPSYLHRDPELCNPNEIAVIGMSCKVAGADDVDEFWDLLCKAESQHQEVPKERFGFE
SAFREVDPTRKWYGNFINEHDCFDHKFFKKSAREIAATDPQQRQMLQVAYQAVEQSGYFTTPKSDKDRKIGCYIGVCAADYEY
NVACHPPNAFMATGNLKSFVAGKISHWFGWTGPGLCIDTACSSSLVAVHQACQAILTGDCTAALAGGANIITHPLWYQNLAAA
SFLSPTGQCKPFDASADGYCRGEGFAAVFLKKMSAAIADGDMIIGSIKATAVNQNQNCTPVFVPNAPTLSDLFRDVLDRSQLT
ANQITVVEAHGTGTQVGDPAEYQSIRNVLGGPSRSTPLLFGSVKGLVGHTECTSGAVSLVKTLLMQQHEAIPPQPSFDRLNPE
IPVSESDNMQIATRFSPWTAEYRAALINNYGACGSNASMVVAQAPRTEQRRSATRRTSVVLDYPFRLCGSDDRALRAYSERLL
RFIASGIKDGISVADLAFNVCRQSNPTLDRSLAFACRTTQEVEEKLRAFVAGNQGLIATSRSKTPREVILCFGGQISNYVGLD
REVYDNVALLRKHLAICDAACRDLGVDSIFPGIFQKSPISDPVKLQTILFSTQYSSAKAWMDSGVRPVAAVGHSFGELTALCA
TGILSLADAMKMIVGRATVIRDFWGEDKGSMIAVEADENRVQRLLAEAAKQCELIHARAPTIACVNGPTSYTLAGPVKSIDIV
TEVISKLSDSGPSIRSKRLKVTNAFHSTLVEPLMEELEKVGQHLTFNTPTMQLERAIKHYSDATLTSDYVYDHMRNPVYFNQA
VQRLAQQYPDSVWLEAGSNSTITSMASRALGSPRSLHFQAVNITSDDSWSMLITSTLSLWKQGISTNFWAYHAEQTYEYNPVL
LPPYQFEPSRHWMELKVPSSMNNGKVQCGARDEEGPPKTLWSLIEASDKVARFQINTAAPKYVELVSGHVIANTAPICPATVE
VDIVVEALRSLRPDFMDSNLQPQVLAVTNQSPICIDPNRSVWLECQAMDSNSVWEWRIVSDSLQEPGTSSSAHVLGKLAFLSG
QDEVKQQESEFMRLERLIGHQRCVDLLNTTEADDIIQGRNIYTTFAGVVDYGEQYRGLKKIVGKGLESAGRVQKKPSEESWLD
AHLGDCFSQVGGIWVNCMTDHNPDDMFIATGFEKWVRSPALRHGQPRSEIWDVLACHHRSSEQTYLTDIFIFDAEQGALTEVI
LGINYHKVAKASMSKILSRLSGTEAAPSSSTRAHPTSSSSPRLPGPSVPEDKSQNETQPAGTNAVAKKKSEKSAQQNVLEKTR
ALLAEISGLEPSEIEAETGLADIGIDSLMGMELARDLEALFKCPLLGDELANVTTFQGLVEYVQSAVGVPTNGDEPDNTNADE
VSEEDNLAPSPSSSSSSTNLTEDSSLDQAETTTNISSYPGQTKTEKPAMPPASSKTLELSPSWVLEAFEESKRLTDHFIEQYR
CANYVDTTLPKQTQLCVALTVEAFEQLGCPIRTAVAGQKLERIIHIPKHAQLAQYLYRLLSADARLIDLTEDGRITRTHMALP
KPSDQILQDLLRLYPDHEWANRLAAFTGARLAEVLKGETDGLGLIFGTDEGRELVAGLYGDSLLNKLSYRQMEDIITRLASRI
PRDSGPLKILEMGAGTGGTTKGMAPLLARLGIPVEYTFTDLSGSFVAAARKKYQKEYPFMKFQVHDIEKPPSDQLRHSQHIVI
ASNAIHATHSLTDSSRHVREFLKTDGFLMIVEMTQPVHWVDIIFGLFDGWWLFADGRDHAIASAGWWEKVFQSVGYGQVDWTD
GHRPEVQIQRVIIAFASGPRYGRQPLPPAPPPNLVPGSHASRQAAVNEYLDKYTKGFTLPAQTSNPDISNSTSYWEKQCVLIT
GATGSLGVHLVAAVAALDDVQTVICLNRRSPMDPDLRQQQAFERRGILLEAASMSKIRVLQTDSSKPQLGLTDEVYSSLVTST
THIIHNAWPMTGKRPLSGLEQQFQVMRNLLDLAAQCSSTRPANVPRIVFQFISSIATVGYYPLWSGQTLVPETCMGIESVLAN
GYGEAKYVCEQMLDRTLHQYPDRFRAMAVRLGQIAGSRTSGYWNPMEHLSFLFKSAQTLQVFPDFTGDLCWTPVNDVAATLSD
LLLLSPHSSSMTDQPIYHIDNPVRQSWSEMVPVLIDALGIPAQNVFPFADWVCRVRAFPGQVEWDNPAALLIDFLDDHFLRMS
CGGLLLDTKRACEHSPTLAAVGPVTAELARKYIQSWKEMGFLNP
>Aspks1
MAAHGQTSKRGNNTLLLFGALVQSHDVSTLRSMRESIVVQHGEHSWLVDSIKALPQDFEAALPHLPFFDQATTTTIHQLLVDA
VSSFLTGSFETLVSPLPAALLIPLAVATQLAHYVEYSRQSPTGLAEGKEALGFCTGILSAFAVASSHDVCDLAKYGAAAMRLG
MLVGLVVDCEDAAAGQGRYRSVSAGWDSEEKHAAMLKIVQSFEEAYVSVHFDKNRATITTSPGTISNLTRQLQKEGLVASDMG
LLGRFHFAGSTKPREVTVDQLVSFCNSPAGALFRLPDADSLRLATRINDRDGGLITQGSLHEHALQSILVKLAAWFETFSSAT
TTQANTGAQNGRARPQIVDFGPQNSVPHSLASTVDINSGNGKTRRVKPADAQSSANSTHTRPWLDTDIAIVGMSCKVPGAENL
EEFWDLLVSGKSQHQEISGQEGGRFDFGDTAFRTAADQRRRWFANLVSNHDQFDHRFFKKSARESASMDPQQRHILQVAYQAV
EGSGYFNKSSSSTPTNANIGCYVGLCLGDYESNVASHPATAFTATGNLQGFVSGKVSHYFGWTGPAVTVNTACSSSLVAVHLA
CQAILSGECEAALAGGSHIMTSATWFQNLAGGSFLSPTGACKPFDSKADGYCRGEGVGAVFLKRMSQAMADGDMVLGVVAATG
VQQNQNCTPIFVPNAPSLENLFSRVMTKARVKPADISVVEGHGTGTAVGDPAEYDAIRKALGGTTHRSADKPLMLSSVKGLVG
HMECTSGVIGMIKLLLMMNKGALPPQASFQSINPALGATPADHMFIPTRPQPWVVPAGGFRAALLNNYGASGSNASAVLVQSP
SMSFRPEITVGSRPAAGIKFPFWLAAFDKKSLSRYVKALRKWLCRLDGDQSLASLSFNLARQSNRTMQANLVLTARSIEALDQ
SLADFENGNDGSFIERTPASSQPTVILCFGGQVSCFVGLDKQVYQDMALVRYYLDRVDAVIQCQGGRSIFPGIFNRSPPSKVD
IVHLHTMLFAMQYASARCWIDSGVKPAALVGHSFGTLTALCISGILSLEDTIKAIMCRAKLLNEAWGPDQGGMIAVEGDIDVI
EELLDEANKNHDDKPATIACYNGPTSFTLAGSTTAMDAVAAQLKNGAKYSKGMKSKRIYVTHAFHSVLVDPLLEELTQRVADS
GVRFRKPIIPVELSTEQHMSESELTSEFMANHMRQPVYFHHAVERLARRYAGGSSPCVFLEAGTNSSVCNMASRALGSTEFVT
KSSSLSFHGVNIANCDAGWNKLTDTTVNLWETGVRVHHWAHHGVQQMHQTDIKPLLVPPYQFDPDSRHWIDLKVPRKALMETD
EADAGGKKQSDAEKLPETILTFHSSDAVGAQKQARFRVNTMLEEYKQLLRGHMTLETAPILSATLQINLVIEAISSTQPEYKS
SKSQPQIQDVVYQSPVCFNSANTLWVEVTNVSGQWMFQVFSTTTQELSPKSTRMVHTKGTVAFKNPGDAEIRRQLMSYERLFS
HGRATDLLQNSNASTAPIDEMLGNQSIYRIFSEIVSYGPEFRGLQKMVSRGNETAGHVVHLKHQDSASTEAEPWFDPHLADTF
CQLGGLWVNCMMPERERGNGHVYLANGIDQWIRGYPAASTDRPEAFNVFAVNKQASEQLTLTDVFVFNAADGALVEVILGIAY
VKIARPSMEKLLARLTEPSWVAGGKTTPQTATKPAAAPVVADHTPRTTESASTVNGVNLDDRKPEGTALPQEMLSDTEELRPK
AQGQELQDMIARVKAVMADISGLDISEIKDDSNLADLGIDSLVGMEMTHEIESTLKVELPESEIMSVVDMEGLLQCVAGALGL
SMTGASSDTLTASSDSGINSAKSSILSGTSTSTSTGTTDTGSDVGQSMKEPSLMLDTVKKAFAQTKEATDARIKAASNQVSYC
STSLPQQNELSVLLTITALEALGAGFSTARPGSQLTRISHAPGHEQFVTHLYKEIETATQIIKIDGHGAQAVITRTAVPLPDV
ESRQVALCEQMLRGDPEQVGTMELIKHAGENLHRVLSGETDGAKVIFGSKTGSKLVSQWYAQWPLNRSLIAQMGDFLTAVVAG
IQADEDMPFSEINPLRIMETGAGTGGTTKQIVPLLARLGLPVVYTFTDLAPSFVAAARKTWGKEYPWMQFRTLDMEKTPPSVE
DGLPLQHFIVSANAVHATKSISATTGNLRKALRTDGFLLMMEMTRTPFWVDLIFGLFEGWWLFEDGRKHALTHEALWDQELSK
VGFGYVDWTEGMTAESEIQKIILASADANTRLERVRLPASHTDYHLNQVGVENEARELMVADYVSTLTKEFNKTMTQYTDAGL
SLSSRTSQTPMSSQKRCILITGGTGGLGAHLVAEAALLPDVNMVICLNRPNRKQEARERQLVSLEKKGLILSPEALAKITVFE
TDLSQPGSLGLSDDKYNLLRGNVTHIIHNAWLMHSKWPVRRFEPQLRIMAHMLNLAADIATCQRTQGQRQPGPPVSFVFVSSI
ATVGYHPVVTNPGNPAVPETRIPISSVLPTGYGEAKYICERMLDATLHQYPAQFRASAVRLGQIAGSEINGHWNSAEHISFLV
KSSQSIGALPALPGPMGWTPADYVARGLVEIATQPDNIELYPIYHIENPVRQPWDEALAVLADEMGISSEALPFQEWVQTVRD
WPRQGDNTAAGANPAYLLVDFLEDHFLRMSCGGLLLGTAKAREHSPSLAGMGPVSDELLRLFVRSWKEVGFLL
>SIPKS4
MDSSTPSFIAFGSLTPWPTPAELTQLRQALLQVQELQPMCEALKELPKLWDTLSNHDSSLQKVEGALAAEQLSQWLLTGQAPT
RDFARNNILSLPLTILSHISDYYVNFHQKESKLDRAKVEGVCAGLLSALAVRSAEGYSQVGSHGALSILLAFSVGTYVDMDAL
QHGQTTCLAVRCKAPATLDTVRDTLKDYDSAYLSVIRNANEATITVPSFCAAQISRRLSADGISVMDTGLTGRYHSQHSLAP
EQISSAFTELGYNLTNDAKKAVHGILVEVSDWSAQFEAANISQNDVVLTIGDAVKCSLPRNTKAIGNDKVKQSTEDLLADYPA

NAIAVVGMSCKLPGADSVEEFWQLLTSGKSMVEQVPEDRWPELAATRGNSKTKKYWGNFLKDIDAFDHRFFKKSAREAASMDP
QQRLLLQAAYEVLESSGYYQPSPNSPSSSTKARDIGCYIGLCAVDYDMNTTCHAPNAFSTLGTLRAFLSGKLSHFFGWSGPSL
TFDTACSSSAVAIHTACRALQAGECSQALAGGVALFTSPYLYENLAAAHFLSPTGATKPFDSKADGYCRGEGLGLVMLKKLSN
AVADGDDVLAVIGGSAINQNDSCVPITVPNAPSQQNLYQNAAKQAGIKPQQVSFVEAHGTGTPVGDPIEMDSIRNVFGGSSRR
NQLVVSSVKGNIGHLEGASGVAGLIKAILQIQHRTAVIQASFQSLNPKIPALAPDNIVIPTTNVPLTDPFLTACVNNYGAAGS
NSTLMVMQPPKTNSIEVSHLSKYPIAIAANSEAAVQEYCRALQDYAFKNPQKMQQMASIAYHLSRRHNQKLPYLITATASNLQ
TAFTQPVAQRKSPISLVLAFGGQVRDSVGLSKEIWNQFAILRLHLDRCDEILRSMGHASLYPAIFQSEPIHELVTLHSAVFAI
QYASAKSWIECGLKVDCVIGHSLGQLTALTVSGILSLQDGLKFVAGRATLMNKYWGPEPGSMILVDANAQSIAKLPHSLETAC
YNGPAAQVMVGEQAAVDSFEDVLTQNGIRFKRLQVANGFHSKFTDPLIEPLTKLAATLRFNKPVIPIETCSLGESWAQVTPEL
LANHTREPVYFHKAAQRISSRGVCTWLEVGSDSGITGMAKRALTTTDDEYLGATLSKSSALDAIIDNTTKLWKSGHATQFWAF
HHSQRSQYENLRLPPYQWEKNRHWLDLLPPKQLELAPANAPVIENEVPQLVKLVSQNSSEAVFRIGVQSEEYQRFVSGHVVAD
SPLCPATVYMEVSARACKMICTWEPAPHLGFSDLKIDSPLGMSASNEITMTLRQLSKDDWEFNVMSSKESNKKVSHATGKIHL
KQDVAAVQRDLSRYARLVRPSMVQALLDDYNSECVRGSMLYKLFSRVVQYSGPYQGLKSAAAKDGCIAGVVTADADDTEGTVL
SQPPTIDSWMQIAGFHSNNFYPCSEDDVYVFTKVDNMQFGPDFDQSNTKSWKIYSNLTPIENNELSNDIFVFDSVSDKLVVLI
LGARFNKVKLNSLSKVLSRLNGTVPTTKKEPVPMKMERQLPLTPSPTEIISKMRCGARETILEGISSVFELVAEVPREEIKGS
ATVDDLGIDSLMMMEVVSELSSYFSIDLPVEDMEGLTDVDSLTGYLLKRGCGQDSAASSTIYSSSSSVSPGTPLTSASSSNEE
QVDQQVEQLAKLLQEHLELDTLPNMCDNLGDLGLDSLLAIELGDDIEKMLSVTIDLHQIDEKSTVGDLVQLAGLNAEANFTSS
IPQVTLSEPMMPVTVAPTESVKSVTPSNTRDIHEAFDEVRLDFDAFAEQEGFTNFWSTVYPAQEELVLSYVADAFRKLGSDIS
TLNSGDSIPSLSVLDKHQHLVKRMHKILMDGGYVSEQDSKYIRTAKRFNLALPQVLLSRIISQFPLHASEHKLLDITGSTLAE
CLTGKADPLTLLFAKKSNRKLLADVYDLAPMCRAATRLLGKFLENAYASNANGQTYHFLEVGGGTGGTTKFVVEYLTRKGVDF
TYTFTDISSALVGAMKKEMSEYDCMRYTTLDVDKPSSELTGQYHAVISTNCVHATGNATAALANLRTLLRPDGIMALVEFTTG
LYWFDLVYGLLDGWWLFSDGRKHALADVPFWEKSMLAAGYQHVNWSDGDTRESRTLRLICGFNSTPSFSSQPTAPIQKRAGVQ
YETVTWKQVGRLDLNADIYYPSERDTNTKRPVAVLFHGGGHVLFTRKDIHIKHVKILLERGFLPISFDYRLCPEVTLSEGPMT
DACDAFQWAREQLSHLRLARPDIQIDENRVVAAGWSAGGQLAMTLGYTAPARGLKAPDAVLAFYCPTNFEDSWWTKPIYPQGI
KEEPGTDYDLLEGVLDKPMTGYKPPTLPGAPMSLQDPRWRIIIHYNWKAQLVPVLVNGLPSKGKSGCHNLKDLPMPPTEKIQA
VSPFAQIVKGKYHTPTFIIHGELDDLIPWQQSRDTIDALKMQGVKAGLATPNAGHAFDLWGDKTDWSSVMEGYDFLQSHS
>AcreC
MDRLPSLLVFGSQSAFPSDDTSGQIRQELIDNPQLSKLLESVKDLPHFWKTLVNFDSRLEKVVGAVSLDQLSQWATGTEPLSL
QGQQPPTVLALPITVLLHITQYTRYLRSSGKFSHSKVLTSLKAGGVQGFCVGFLSAIAVAVSGSEDEVSSWGAVAFRLAVAIG
AYVDLDSIENARQHAAIAIRWRSGNKHDRALVGELIDSVSSAYVSSINDDTSLTITLEGDDVSKLIEAAHGKGLHTKRVPVQG
RFHTTDYSETVENLYSLASQNAELKFPDADDLRIRLRDTKDGSVVTQGSLTRSALENTLLHVADWYSTMKSSIEVLSGTSSTI
AVAGAGGVVPASLSSKSGLQVVQLSTIVEPKTTHTKDFLKAHINGVAGFDGIHEVSENDDLSEYPSHSIAIVGMAGRFPGADS
VDELWDLIQEGKTMVEPAPVDRLALPQTGDHAETKWWGNFLKDPDAFDHKFFKKPSREALAWDPQQRILLEVIYQALESASYF
GPSSTDEPLDYGCYIGAVMNNYYDNLSCHAGTAYATVGTSRCYLSGCMSHYFGWTGPSLTIDTACSSSLVAINTACRAIWSGE
CSRAVAGGTNVISSPFDYQNLAAAGFLSPSGQCKPFDADADGYCRGEAVAVVVLKPLADAMKDNNNILGVVTGSAANQNYNLA
HITQPQSTSQVELYQNVVRLSGVEAEAVSYVEAHGTGTGVGDPIEVRSIRDAFGGPQRDSLLHFSSIKGNIGHTEATAGVAGL
IKVLLMMRHGRITAQASHNSLNPKLPDFQHHQMVIPRKNMDWEAPYFLACVNSYGAAGSNSAVMIRQSPHRAKGTPSTRQLTK
FPIFISAGSQDGLVRYAKKLLEFIETWKGVDESKLLASLAFTLSDRGNHALPSRLSVVVESLGQLQSKLKSVIDGSVSDISQL
SKESRSIVLVFGGQESNFIGLTREVYETSSIFRYHLDKVNNLLIASGQASFFPSIFESDPIDNLVILHSGLFAVQYASAMAWI
ESGLSVSAVVGHSFGQLTALCISGALPLADALKLVSGRASLMQQHWGPEPGSMLFLQADRTTVEDLIHSVQAPYAEVACYNGP
QSHVVVGTSDSITAIQHHVETTPQLRASVRTKKLNVTNGFHSEFTEPLLPHLVALAEELEWRTLTIHLETTEELPHSGKLGSD
FVSDHTRKPVFFQSAIERLASQFPQATWLEAGRGSSVMQLVKLSVPNPQSQVFHSPQLTATTAQSSLADITIDLWKKGQATQF
WPFHKSQKFEYESLSLPPIQFEKTRHWLGFTSREPASEKEPDVVKEVEAAHELLRFVNFRDKAKSEAEFSIDPKADRFQQMLG
GHVMAGQALAPASLYFEVVARAALLLENATEAQRFVPTVNNLLMRSPIGRDTDKRIILVLKKTQGMHSSWSFSIATQDLGANA
AQPFEVSTGEVILKRKDDSKATREFERFETLIGRSRCDDILAHPDAENMQGNHIYRAFNTVVYYGEDFRGIKHVACLRNEAAG
KVRITPPNTDPADQRLCDTPMTDSFMQFAGFLVNYFNNPSMDDVFVCMKIEHIEIGGDFDPDAGEWLIYSTMSEGGETDASSD
AYIFDARTNKMVMAAFGFRFSKMSQNLLGRMLRSVNKSSNPKAAAKEDIPVDESHTRPVETAPASISTRKAEGKRKEVLQILS
NVTDIPLNEIHDESTLDDLGVDSLMGTEVLNDIRAVLGLTIDLSSFLFFPNIQALIAHVNGQLGLETDEESSGTDSGLDDADT
PFNNNSQSGNTTPEEIPLEDSITFKSVERPSIKSASGAFEETRLNYDYLAESTGAAGFWRDAYPHQARLVLAYVVEAFASLGC
DLKRTSAGAQVPQIKALDKHKLLVSQLYRVLEDGKLIEKSHSDSQRFVRTTSQVEPRSAESIFHEVVDLYPQHAVVIKLVRAV
GSEFAACLRGDKEGIQLVFGNRETKKNLEEMYEFWPLLRTPALVLGDFLAKAFTHRASGTGKFRILEIGAGTGGTTRFIVNHL
KSLGIDFEYVFTDLGASLVNAAQKQFKGVDNMTFDVLDLEKPPKPDYQGAFHCVLATNCVHATRDLDLSLKHARSMLRDDGML
TLIEITQNMFWLDIVVGLFEGWWLFNDGREHALVDEKHWERRMKGAGFEEVAWSDGATPESKTVRLIAAFPKKAPEKPAKAGF
ETVVYKTVDDLDIQADIYYPLEGEVLPVEKMPVALMIHGGSHMLFTRKDVRPAQTRLLLSKGFLPVSIDYRLCPEVALMEGPM
VDTVDALEWAQTKLPSIKLPRSDLKIDGSRVVVVGWSSGGQLAMSLAWTAPQRGLRPPEAILTFYSPTDYEDSWWRNPIQPIG
GEYQGQTYDILEGVADKPIANYAMVGAWEEPIADPRSWNDLRCRLIFHINWKAQTLPVILNGLPNRKTATSKFPEVQDWNALP
QPSLETIQAASPRAHVLRGTYKIPTFFIHGTADDLIPWQQTQRTYETMRDSGIETNVVLIKDGPHICDLSSDPNSEGWKAVLK
GYEFLASHAL
